# Dental Manifestations in Children Affected by Hypophosphatemic Rickets: A Systematic Review and Meta-Analysis

**DOI:** 10.3390/children12020144

**Published:** 2025-01-27

**Authors:** Aesha Allam, Silvia Cirio, Francesca Elia, Claudia Salerno, Maria Grazia Cagetti

**Affiliations:** 1 Department of Biomedical, Surgical and Dental Sciences, University of Milan, 20112 Milano, Italy; aesha.allam@unimi.it (A.A.); silvia.cirio@unimi.it (S.C.); francesca.elia1@studenti.unimi.it (F.E.); claudia.salerno@students.unibe.ch (C.S.); 2 Department of Restorative, Preventive and Pediatric Dentistry, School of Dental Medicine, University of Bern, 3012 Bern, Switzerland

**Keywords:** hypophosphatemic rickets, children, dental manifestations, dental abscess

## Abstract

Background: Hypophosphatemic rickets (HR) is a bone disorder affecting phosphate–calcium metabolism, with both skeletal and dental manifestations. This review aims to analyze dental manifestations of HR in children and, where possible, compare them to those in healthy children or affected adults. Methods: The protocol was registered at PROSPERO (CRD42024596022). The study conformed to the PRISMA guidelines. Three databases were searched for studies reporting the prevalence or incidence of any dental manifestation in children with HR. Risk of bias was assessed using JBI, RoB 2.0, and ROBINS-E tools, and Stata/SE 18.0 was used for meta-analysis. Meta-regression was used to examine the effects of therapy duration and mean age on dental manifestations’ prevalence. The study received no funding. Results: A total of 1308 records were identified, with 660 screened after removing duplicates. Forty-six studies were eligible for full-text evaluation; sixteen were included in the qualitative analysis and twelve in the meta-analysis. The dental manifestations observed included dental abscesses, developmental defects of enamel and dentin, dental caries, taurodontism, and large pulp chambers. Dental abscesses were the most common manifestation, with a pooled prevalence of 0.39. Meta-regression showed no association between therapy duration and abscess occurrence but revealed a negative association between mean age and abscess prevalence. Conclusions: Dental abscesses were the most frequent manifestation in children with HR. The role of therapy in improving oral health remains unclear due to insufficient data, indicating a need for further studies on the impact of HR on children’s oral health.

## 1. Introduction

Rickets is a metabolic bone disorder that includes a heterogeneous group of conditions characterized by disturbances in calcium and/or phosphate metabolism, resulting in impaired mineralization of growing hard tissues [1,2]. The global prevalence of rickets varies significantly and is shaped by a range of socioeconomic, cultural, and environmental factors. In industrialized countries, the prevalence of nutritional rickets has markedly declined over the past century, largely due to the implementation of vitamin D fortification programs in food [3]. Current estimates indicate case rates of nutritional rickets ranging from 2.9 to 27 per 100,000 individuals in the United States and Europe [4]. However, recent reports have highlighted a resurgence of rickets cases in various regions, including industrialized countries, over the last decade [3]. This re-emergence is primarily attributed to factors such as increased use of sunscreen, which reduces sun exposure, and rising immigration rates from areas with a higher burden of rickets [3]. Rickets disease primarily affects children during growth and is typically caused by deficiencies in key nutrients, including vitamin D, calcium, or phosphate, which are essential for proper bone metabolism [1]. Inadequate sun exposure, malnutrition, genetic disorders, and certain medical conditions can also contribute to the development of rickets [2].

Rickets can be classified into two major categories according to the underlying mineral deficiency: calcipenic and phosphopenic rickets [1]. Calcipenic rickets includes a broad category of rickets caused by insufficient calcium, primarily due to inadequate availability or defective functioning of vitamin D [1].

The other major category of rickets is phosphopenic or hypophosphatemic rickets (HR), which includes a broad category of disorders caused by excessive renal phosphate excretion [1,5,6]. The historical nomenclature for this group of disorders includes terms such as vitamin D-resistant rickets (VDRR) and familial HR [7]. Several genetic mutations have been identified as an underlying cause of HR, each associated with a distinct form of the disorder. These forms include autosomal recessive hypophosphatemic rickets (ARHR), autosomal dominant hypophosphatemic rickets (ADHR), and X-linked hypophosphatemic rickets (XLHR) [1,6,8,9]. XLHR is caused by mutations in the PHEX gene, and it is the most common form of HR [9,10,11,12], with an estimated prevalence of 1:20,000 [11].

Diagnosis of HR in children is based on typical signs and symptoms supported by laboratory, radiological, and genetic findings [13,14]. Clinical manifestations encompass both skeletal and extra-skeletal manifestations [15,16,17,18,19].

Among the extra-skeletal manifestations of HR, a variety of oral manifestations have been reported, including a wide range of dental problems, mainly affecting the development of mineralized dental tissues. Enamel and dentin are particularly susceptible to the interplay of systemic and local factors, such as disruptions in calcium and phosphate metabolism, as well as loss-of-function mutations, such as PHEX gene mutations in XLHR [12]. These disruptions, along with hypocalcemia, negatively affect dental cell function and mineralization processes, leading to severe inhibition of mineralization [20]. Numerous case reports have documented mineralization defects in both enamel and dentin associated with HR [21,22,23]. Scanning electron microscopy (SEM) has revealed alterations in circumpulpal dentin in contrast to the often normal mantle dentin. These alterations include widened dentinal tubules and large interglobular spaces between calcospherites, which fail to merge during the mineralization process [21,24]. Moreover, fissures extending from the enamel surface to the dentino–enamel junction may also be observed, potentially facilitating the entry of oral bacteria. Dental defects can lead to spontaneous dental abscesses in children, which are not associated with prior carious lesions or trauma. These abscesses are considered a hallmark of rickets disease [20,23,25]. Several investigations reported an association between poorly mineralized dentin and large pulp chambers, providing an easy pathway for oral bacteria to cause pulpal infections [23]. These abscesses are reportedly more frequent in the deciduous dentition, and often present in a recurrent pattern. Other dental manifestations include dental developmental defects such as taurodontism and root malformations. Gingivitis and periodontitis in older subjects are also reported [26].

The treatment of HR has evolved significantly over the years as the understanding of its pathophysiology has advanced. For many years, conventional therapy has primarily involved the administration of vitamin D supplements and analogs, in addition to which phosphate supplementation was often required [17,27]. In recent years, a more targeted approach for the treatment of XLHR has been introduced with the use of burosumab [28].

Although oral manifestations in rare bone diseases, including HR, have been explored in a previous review [29], to the best of the authors’ knowledge, currently no systematic reviews have specifically examined the relationship between HR and its dental manifestations in children. Accordingly, this systematic review aims to provide a comprehensive assessment of the oral health status of children with HR. Comparison with the dental status of affected adults will also be presented. By examining the progression of dental health over time, this review seeks to provide a detailed overview of how disease impacts oral health across the lifespan. Furthermore, the anticipated findings could provide dentists with a deeper understanding of the oral problems affecting children, facilitating earlier diagnosis and prompt intervention when needed.

## 2. Materials and Methods

### 2.1. Prospero Registration

The systematic review protocol was registered at the International Prospective Register of Systematic Reviews (PROSPERO), registration number: CRD42024596022 (https://www.crd.york.ac.uk/prospero/display_record.php?ID=CRD42024596022) accessed on 11 October 2024. The registered protocol underwent minor modifications during the literature search phase, as detailed in Supplementary Table S1.

The review adhered to the methodologies outlined in the Cochrane Handbook of Systematic Reviews. It conformed to the guidelines set forth by the Preferred Reporting Items for Systematic Reviews and Meta-Analysis (PRISMA) [30]. The PRISMA checklist is presented in Supplementary Table S1.

### 2.2. PECOs Question

The primary question addressed in this review was “What are the dental manifestations in children with hypophosphatemic rickets?”. A secondary question explored was “Are there differences in dental manifestations between children and adults?”. The elements of the PECOs model used were as follows:

- P (Participants): children of any age or sex;
- E (Exposure): HR for which patients received any form of treatment;
- C (Comparison): healthy children or adults affected by HR. Studies with no comparison group were also considered;
- O (Outcome): any dental manifestations, including dental abscess, dental caries, periapical radiolucency, previous endodontic treatment, dental developmental defects, and large pulp chamber, and gingivitis and periodontitis;
- S (Studies): observational (case–control, cohort and cross-sectional), randomized controlled trials, case series studies, and surveys.

### 2.3. Information Sources and Search Strategy

Three databases (PubMed, Embase, and Scopus) were searched up to 30 November 2024. The search strategy was initially developed for PubMed using keywords and MeSH terms and adapted to the other databases. Search strings used for each database are displayed in Supplementary Table S2. Cross-referencing was also performed using the reference lists of full-text papers.

### 2.4. Study Selection, Eligibility Criteria, and Data Extraction

At all stages, reviewers were trained and, before screening began, a pilot test was conducted to verify proper adherence to the eligibility criteria. After removing duplicates, records were assessed based on title and abstract by two authors (AA, FE). Disagreement was solved by discussion, and when it was not possible, a third author (SC) was consulted. Subsequently, the same two authors proceeded to full-text analysis; disagreements were resolved by debate or involvement, where needed, with the same third author (SC).

Studies were evaluated based on the following inclusion criteria: availability of the full text in English, publication year after 1980, and a focus on any form of HR. Surveys or questionnaires from patients or caregivers reporting dental status were deemed eligible. Exclusion criteria included studies focusing on forms of rickets other than HR or bone diseases with manifestations resembling rickets. Additionally, case reports and case series involving fewer than five subjects were excluded. Studies that reported only the overall prevalence of dental problems without a detailed breakdown of specific prevalence rates for each dental problem or those that lacked separate data for children and adults were also excluded.

Data extraction was performed independently by the two reviewers (AA, FE). The following data were collected and inserted in an Excel^®^ extraction form sheet: bibliographic information (authors, publication year, country), type of study, type of HR, therapy implemented, study population (mean age in years, number of participants, male-female ratio), dental outcome, method of dental evaluation (clinical examination, clinical records consultation, dental history taking, or questionnaire), type of dental manifestations observed in rickets patients and healthy controls (dental abscess, periapical radiolucency, endodontic treatment, dental developmental anomalies, large pulp chamber, developmental defects of enamel (DDEs), dentin defects, gingivitis and periodontitis), and funding source. The data extraction form is available in Supplementary Table S3.

### 2.5. Risk of Bias Assessment

The risk of bias assessment was carried out by three reviewers independently (AA, SC, and CS), using different tools according to the type of study evaluated. Accordingly, the Joanna Brigg’s Institute (JBI) critical appraisal tools were used for case–control [31], cross-sectional [31], and case series studies [32]. Each tool is composed of questions, to which reviewers had to answer with yes, no, unclear, or not applicable. The overall risk of bias was considered “low” when all criteria were met, or no more than one criterion was judged unclear; “moderate” if 2 criteria were judged unclear, or one criterion was not met, and the others were met; or “high” if three or more criteria were judged unclear, or two criteria were not met, and the others were met. Randomized controlled trials were assessed using the Cochrane Collaboration’s RoB 2.0 [33] and ROBINS-E tools for exposure studies involving only one group of participants [34]. Both tools offer a series of questions. Upon completion, an embedded algorithm within each tool assesses the responses and assigns a risk of bias score to each domain. Subsequently, an overall risk of bias score is calculated. For all tools, confounding factors considered included the following: age at therapy initiation, age at diagnosis, and the type of therapy administered.

### 2.6. Statistical Analysis

Stata/SE 18.0 for Mac (Intel 64-bit) was used for the meta-analysis that was performed if three or more studies included comparable findings. The sample size, together with the number of subjects with the various dental manifestations, were extracted or calculated for each study. Whenever a study provided a detailed breakdown of the number of subjects receiving a specific treatment or no treatment, those values were prioritized and used for the analysis. However, if only the total number of subjects was available without a breakdown, the total was used.

Using the I2 statistic, the proportion of variation in the effect estimates due to heterogeneity rather than chance was determined. The meta-analysis was conducted using a random effects model, due to the high level of methodological heterogeneity of the included studies. The random effects model was selected to account for potential variability between studies, assuming that the outcome might differ due to variations in study populations or methodologies. This approach allowed a more conservative and generalized estimate, since heterogeneity across studies was anticipated. The results of each meta-analysis were graphically presented by Effect Size of Forest plots. To explain the high heterogeneity, subgroup meta-analysis (based on type of data collection, type of rickets, type of therapy) or meta-regression (based on age and years of therapy) were performed when possible.

Publication bias was evaluated using a funnel plot approach, and asymmetry was identified through Begg’s and Egger’s correlation tests. Where meta-analysis was deemed inappropriate, findings were not pooled; instead, a qualitative description was provided.

## 3. Results

### 3.1. Search Results

**Figure 1:**
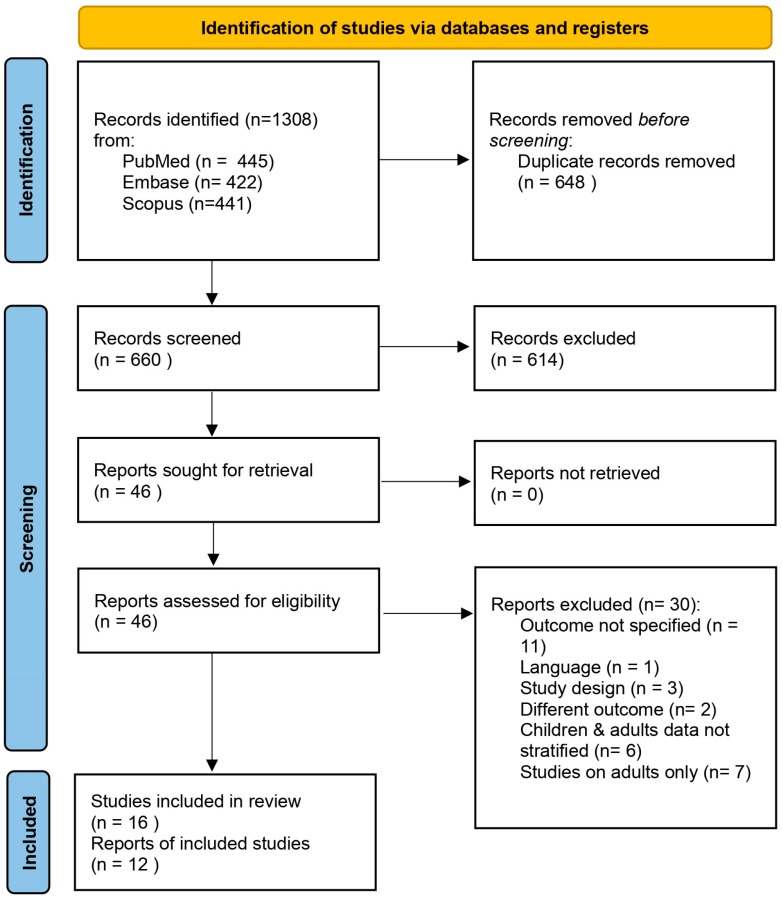
PRISMA flowchart.

The database search results are presented in the flowchart shown in Figure 1. The search yielded a total of 1308 records. After removal of duplicates, 660 records were screened based on title and abstract, and 614 were excluded (Supplementary Table S4). Consequently, 46 records were deemed eligible and progressed to full-text evaluation (Supplementary Table S5). Although six studies partially met the inclusion criteria, they were excluded because they reported data on both children and adults without stratification [35,36,37,38,39,40]. Additionally, seven studies were excluded, as they only reported data on adult subjects [41,42,43,44,45,46,47]. Consequently, sixteen studies were included in the qualitative analysis and twelve in the meta-analysis [26,48,49,50,51,52,53,54,55,56,57,58,59,60,61,62]. Cohen’s Kappa value for inter-reviewers’ agreement was 0.61 at the title and abstract screening and 0.82 at full-text screening.

### 3.2. Studies’ and Samples’ Characteristics

#### 3.2.1. Study Types and Geographic Distribution

Characteristics of the included studies are shown in Table 1. Studies were conducted in Italy [48,49,51], USA [58,59], UK [53,60], France [52], Israel [50], Peru [54], Japan and South Korea [55], Japan [57], Chile [56], and Iran [62]. Two studies were multicentric [26,61]. Regarding the study types, two studies were cross-sectional [56,62], and one was a cross-sectional survey [55]. Three studies were case series [49,53,58], three were case–control [48,51,57], one randomized controlled trial [61], four retrospective [26,52,54,60], one prospective [50], and one controlled longitudinal study [59]. Studies were published between 1986 [58] and 2024 [57], with eleven studies published in the last decade.

#### 3.2.2. Samples’ Sizes and Age Groups

Sample sizes of the included studies ranged from 9 to 579 subjects, with the latter being a multicentric study [26,49]. Eleven studies considered only pediatric patients [48,49,50,51,52,54,57,59,60,61,62], with the mean ages of children ranging from 5.80 to 10.70 years [48,59]. The remaining five studies included children and adults and provided separate data for each age group [26,53,55,56,58].

#### 3.2.3. Types of Rickets and Therapy Implemented

The types of rickets diagnosed among the different study populations are shown in Table 2. Five studies considered patients affected by HR in general [53,54,56,60,62]. The majority of the studies, totaling eleven, focused on subjects affected by XLHR [26,48,49,50,51,52,55,57,59,60,61]. Only one of the included studies, the oldest among them, used the outdated nomenclature, reporting the inclusion of subjects affected by VDRR [58].

The most implemented therapy regimen across studies was conventional treatment, which involved vitamin D supplementation and its analogs, often combined with phosphate supplementation [48,49,51,56,58,60]. Reports on the use of burosumab therapy for the treatment of XLHR have exclusively appeared in studies published from 2022 onward [26,50,52,55,57,61]. In most studies, children began treatment promptly after receiving a diagnosis of rickets. In five studies, patients received either conventional or burosumab therapy [26,52,55,57,61]. Specifically, in one study, subjects were followed during their transition from conventional therapy to burosumab [50]. In four studies, the therapy implemented was not specified [53,54,59,62].

The mean duration of therapy in the pediatric population ranged from 3.50 to 8.01 years [48,51].

### 3.3. Dental Manifestations and Studies’ Outcomes

#### 3.3.1. Dental Infections

Dental infections, including dental abscesses, fistulas, and periapical radiolucencies, were the most frequently evaluated outcomes across the included studies, as shown in Table 2. Dental abscesses were assessed through various methods, including dental examinations conducted during the study, reviews of clinical records, dental history interviews, or questionnaire administration.

The prevalence of dental abscesses in children, as determined through dental examinations, ranged from 10.00% to 33.00% [51,56]. Data collected through dental history interviews reported higher prevalences, ranging from 37.50% to 66.66% [48,49,58]. Clinical records consultation revealed prevalences ranging from 10.00% to 58.80% [54,57]. One study reported a prevalence of 14.30% among children, based on a questionnaire administered to caregivers [55]. Four studies specifically identified the nature of dental abscesses in children as being spontaneous, not associated with carious lesions or previous trauma [48,49,53,56]. One study conducted a three-year follow-up of children initially receiving conventional therapy [50]. At baseline, 30.00% of the examined children presented dental abscesses. After transitioning to burosumab therapy, the prevalence of dental abscesses decreased to 10.00% after one year and remained steady after three years. Another study compared the monthly incidence of dental abscesses between two groups receiving different therapies over a 41.5-month follow-up period. [52]. The findings showed that the number of dental abscesses per month during the follow-up period was significantly lower in the burosumab group (0.01 ± 0.03) compared to the conventional therapy group (0.04 ± 0.05). However, another study reported a lower incidence of dental abscesses only in children under 5 years of age who received burosumab therapy, compared to those on conventional therapy [61]. In this age group, the overall incidence was 11.53%, with 0% in the burosumab group and 25.00% in the conventional therapy group. Conversely, among children aged 5 years and older, the overall incidence was higher at 22.85%, with 53.33% in the burosumab group and 0% in the conventional therapy group.

In the adult population, the prevalence of dental abscesses ranged from 37.27% to 89.00% [26,56]. In one study, a questionnaire administered to adults revealed a prevalence of 21.90% [55].

One study reported the prevalence of periapical radiolucency in children to be 28.57% compared to 25.00% in healthy children [51]. Endodontic treatment in the pediatric population was analyzed in two studies, with reported prevalences of 1.25% and 16.66% [26,49]. In the adult population, the prevalence of endodontic treatment was higher, ranging from 10.00% to 73.68% [26,56].

#### 3.3.2. Dental Caries

Five studies reported the prevalence of dental caries in children [26,49,54,56,62], with rates ranging from 12.55% to 90.00% [26,54]. One study compared the percentage of caries-free children with XLHR with healthy controls, reporting that 70.00% of children with XLHR were caries-free, compared to 66.66% of healthy subjects [51]. Additionally, a questionnaire administered to caregivers of children in one study revealed that 28.60% of the children were affected by caries [55].

Two studies reported the incidence of dental caries in children receiving treatment with conventional and burosumab therapy. Specifically, one study reported higher caries incidence in the burosumab group compared to the conventional therapy group in both children aged < and ≥5 years after a follow-up period of 64 weeks [61]. In contrast, the second study reported no difference in caries incidence between the two treatment groups after a mean follow-up period of 41.50 months [52].

One study reported a prevalence of caries in the adult population of 13.63% [26]. In one study, a questionnaire administered to adult patients revealed that 37.50% were affected by caries [55].

#### 3.3.3. Pulp Chamber Size

The prevalence of large pulp chambers was reported in four studies based on radiographic examination [51,53,56,58]. In children, the prevalences ranged from 28.57% to 62.25% [51,53]. Among adults, two studies reported prevalences of 60.00% and 94.73% [56,58]. The pulp chamber/tooth area ratio was assessed in three studies [48,50,57]; a statistically significant difference (*p* < 0.0001) between children affected by HR and healthy controls was reported [48].

The effect of burosumab therapy on pulp dimension was assessed by comparing pulpal coronal height and width ratios at baseline (before therapy initiation) and after 1 year and 3 years [50]. In children with rickets, higher baseline values were found, with a pulpal coronal height ratio of 0.32 ± 0.07 and a pulpal coronal width ratio of 0.48 ± 0.11. In contrast, healthy participants had baseline values of 0.22 ± 0.05 for the pulpal coronal height ratio and 0.38 ± 0.11 for the pulpal coronal width ratio. A slight improvement of these values was observed over the 3 years of follow-up, particularly in the pulpal coronal width ratio, which decreased to 0.40 ± 0.11 after 3 years of burosumab therapy.

The difference in pulp/tooth ratio among children with XLHR across different age groups was assessed [57], comparing those with and without dental abscesses. No statistically significant differences were observed, except in patients aged 5–7 years (*p* = 0.0180). Additionally, no difference was found between conventional therapy and burosumab therapy.

#### 3.3.4. Developmental Defects of Enamel and Dentin

DDEs were assessed in five studies. Two studies reported only the prevalence of quantitative defects of enamel, referred to as enamel hypoplasia [54,62], with prevalences of 20.00% and 42.10% [54,62]. Only one study made a distinction between qualitative and quantitative defects [49], referring to them as dischromic alterations and enamel hypoplasia, with prevalences of 22.22% and 11.11%, respectively. Another study reported an overall prevalence of 35.29% of enamel defects, but did not specify whether the defects were quantitative or qualitative [53].

One study investigated structural alterations of enamel by examining tooth replicas with electron microscopy [51]. All children affected by XLHR exhibited some form of enamel structural alterations, in contrast to healthy children who exhibited no enamel alterations.

Three studies reported data regarding dentin defects, evaluated through X-ray examination and described as radiolucent, hypomineralized, or thin [26,53,54]. One study reported no dentin defects in children but reported a prevalence of 1.81% in adults [26]. In contrast, the remaining two studies reported prevalences of dentin defects of 12.50% and 20.00% in children [53,54].

#### 3.3.5. Other Dental Manifestations

Other dental developmental anomalies in the included studies include taurodontism, malformed roots, ectopic eruption of permanent canines, and dental agenesis.

Taurodontism prevalence in children was analyzed in five studies [26,53,54,58,62], with prevalences ranging from 0.80% to 43.75% [26,53]. One study reported the number of teeth affected, highlighting that the condition exclusively affected male children, with no cases observed in females [59]. The study also compared these findings with a group of healthy subjects, in whom taurodontism was absent. Additionally, one study reported that short malformed roots were observed in 10.00% of the children evaluated [54].

Ectopic eruption of permanent canines was reported in one study [59], revealing a higher prevalence of 21.00% in children with XLHR compared to 5.20% in healthy controls.

Periodontal health was assessed in terms of the prevalence of gingivitis and periodontitis. The prevalence of gingivitis in children was reported in three studies [26,54,62], with rates ranging from 1.67% to 70.00% [26,54]. Two studies reported prevalences in the adult population of 0.90% and 25.00% [26,56]. Only one study assessed periodontitis in the pediatric population, reporting a prevalence of 20.00% [54]; two studies assessed the adult population, and the prevalences were 2.58% and 70.00% [26,56].

### 3.4. Risk of Bias Assessment Results 

**Figure 2:**
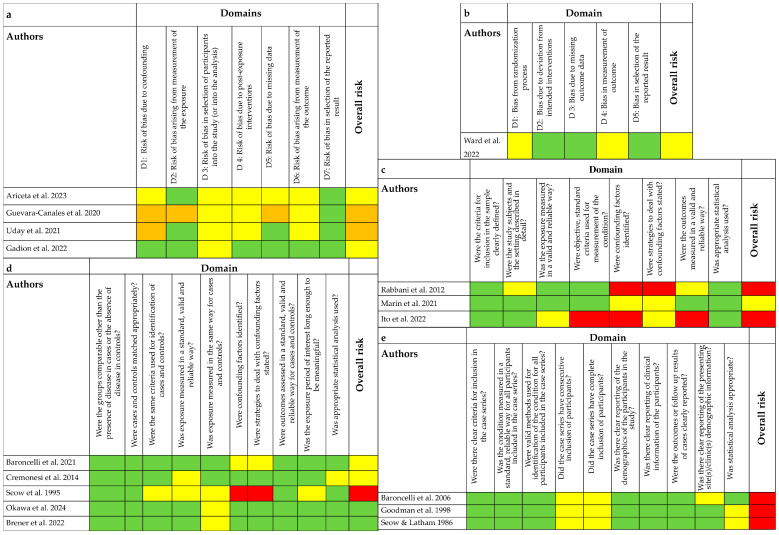
Risk of bias assessment. (**a**) Studies involving only one group [26,52,54,60], green = low, yellow = some concerns, orange = high. (**b**) RCT [61], green = low, yellow = some concerns. (**c**) Cross-sectional studies [55,56,62], green = yes, yellow = unclear, red = no, overall risk: green = low, yellow = moderate, red = high. (**d**) Case–control studies [48,50,51,57,59], green = yes, yellow = unclear, red = no, overall risk: green = low, yellow = moderate, red = high. (**e**) Case series [49,53,58], green = yes, yellow = unclear, overall risk: red = high.

Four studies were assessed using the ROBINS-E tool, with the results presented in Figure 2a [26,52,54,60]. Two studies were found to have a high risk of bias [54,60], while the remaining two were categorized as having some concerns [26,52]. The domain most frequently assessed as high risk of bias was related to confounding factors, as studies often failed to address variables such as age at therapy initiation, type of therapy received, and the mean duration of treatment. One study [61], as shown in Figure 2b, was assessed with the RoB 2.0 tool and was judged to have some concerns. Three studies were assessed using the JBI critical appraisal tool for cross-sectional studies [55,56,62], with results shown in Figure 2c. Two studies were found to have a high risk of bias [55,62], and one study was assessed as having a moderate risk of bias [56]. Questions related to confounding factors and methods to deal with them received the lowest judgment across studies. Five studies were assessed using the JBI critical appraisal tool for case–control studies; results are shown in Figure 2d [48,50,51,57,59]. Two studies were judged to have a moderate risk of bias [49,51], two studies had a low risk of bias [50,57], and the remaining study had an overall high risk of bias [59]. Again, questions about confounding factors and methods to handle them received the lowest ratings across all studies. Three studies were assessed using the JBI critical appraisal tool for case series, with results presented in Figure 2e; all were judged to have a high risk of bias [49,53,58].

### 3.5. Meta-Analysis

For each dental manifestation, a meta-analysis was performed.

**Figure 3:**
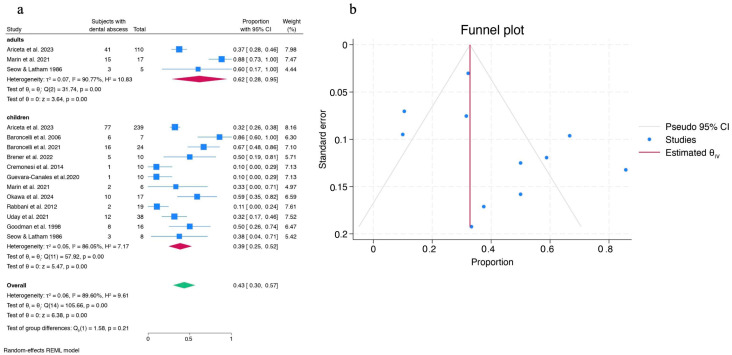
Forest plot of children and adults with dental abscesses (**a**) and funnel plot for the forest plot of children (**b**) [26,48,49,50,51,53,54,56,57,58,60,62].

Regarding abscesses in children with HR, the pooled prevalence from twelve studies was 0.39 [0.25, 0.52], with high heterogeneity (I^2^ = 86.05%). Among adults, the pooled prevalence was higher at 0.62 [0.28, 0.95], although the test for group differences was not significant (Figure 3a). The funnel plot for the forest plot in the pediatric population showed slight asymmetry for studies with larger sample sizes, but reasonable symmetry for smaller studies (Figure 3b). The regression-based Egger test for small-study effects (β_1_ = 1.20; z = 0.87; *p* = 0.38) indicated no significant evidence of small-study effects in this meta-analysis.

Among children, the high heterogeneity was further explored through two meta-regressions. The first examined whether the number of years of therapy influenced the prevalence of abscesses, but no significant association was found (z = *−*1.57; *p* = 0.116). The second meta-regression revealed a clear negative association between the mean age of the study sample and the prevalence of abscesses in children with rickets. For each additional year in the sample’s mean age, the prevalence of abscesses decreased by 0.1447 in proportion terms (z = *−*3.74; *p* < 0.0001). The high percentage of variance explained (R^2^ = 77.46%) suggests that mean age is a highly relevant predictor.

**Figure 4:**
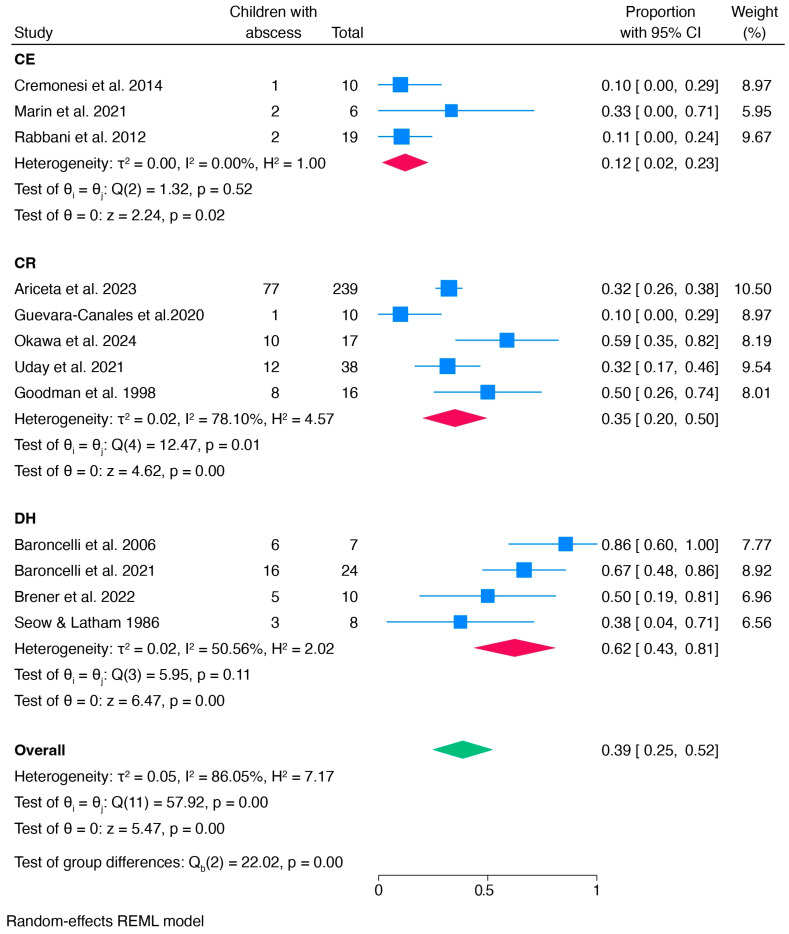
Subgroup meta-analysis: forest plot of children with dental abscesses divided by different methods of data collection (CE: clinical examination; CR: clinical records; DH: dental history) [26,48,49,50,51,53,54,56,57,58,60,62].

To further investigate heterogeneity, a subgroup meta-analysis was performed based on the method of data collection (Figure 4). Stratification by data collection method reduced I^2^, and prevalence differed significantly across the three subgroups (clinical examination, clinical records, and dental history) with strong evidence (*p* < 0.0001). Prevalence was lower in studies using clinical examination (0.12) and higher in those relying on dental history (0.62).

**Figure 5:**
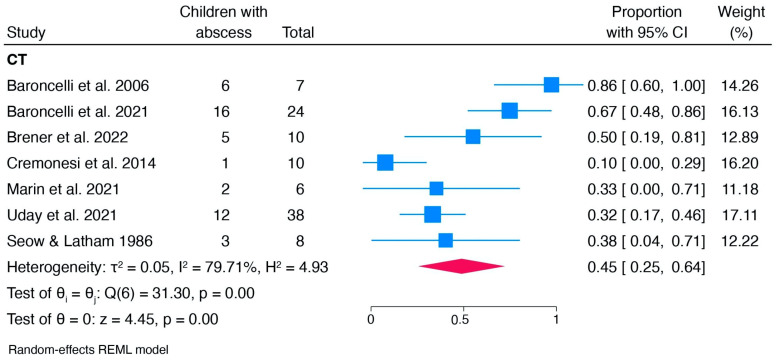
Subgroup meta-analysis: forest plot of children with dental abscesses who received conventional therapy (CT) [48,49,50,51,56,58,60].

An additional subgroup meta-analysis examined the type of therapy the children received. In the group receiving conventional therapy, the pooled prevalence was 0.45 [0.25, 0.64]. No test of group differences was performed due to the limited number of studies in the other groups (burosumab and no therapy) (Figure 5).

Lastly, analysis was conducted to investigate whether the risk of bias influenced the prevalence of abscesses: the test of group differences (*p* = 0.14) showed no differences in pooled prevalence among the subgroups (high, low, moderate, and some concerns), suggesting no influence of the risk of bias on the observed heterogeneity (Supplementary Figure S1). Another subgroup analysis was performed according to rickets type, but the results were not significant and are presented in Supplementary Figure S2.

**Figure 6:**
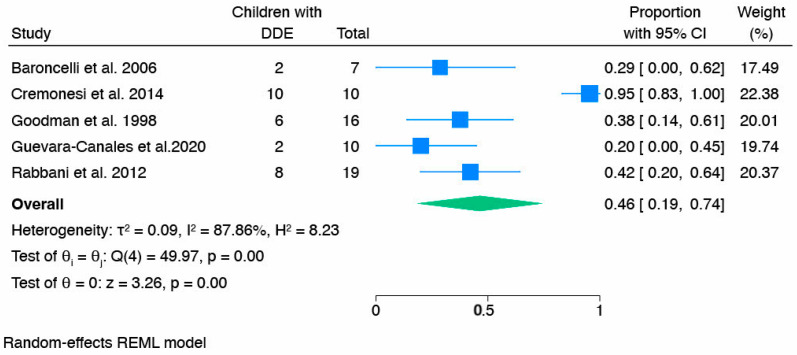
Forest plot of children with developmental defects of enamel (DDEs) [49,51,53,54,62].

The second type of dental manifestation analyzed DDEs. The pooled prevalence of DDEs in children with HR was 0.46 [0.19, 0.74] (Figure 6). However, no further analyses were conducted to explain the heterogeneity due to the small number of included studies.

Meta-analyses of the pooled prevalence of caries, endodontic treatment, gingivitis, taurodontism, and large pulp chambers were performed and are reported in the Supplementary Materials due to the limited number of studies (Supplementary Figure S3a–e).

## 4. Discussion

### 4.1. Main Findings

The present review included sixteen studies that examined dental manifestations in children with HR.

Dental abscesses were the most frequently reported outcome across all studies, often occurring spontaneously without any association with carious lesions or prior trauma.

The causes of this spontaneous onset were explored in literature and often attributed to defects of mineralization of dentin and enamel, as well as abnormally large pulp chambers. The included studies demonstrated altered pulp/tooth ratios in both dentitions, primary and permanent, with high pulp horns potentially facilitating bacterial penetration and increasing the risk of dental abscesses. However, findings regarding the relationship between pulp chamber size and dental abscesses suggested a more nuanced reality, as one study reported that one-third of children with large pulp chambers did not develop dental abscesses [48]. No difference in pulp/tooth ratios was observed between children with and without dental abscesses [57]. These results suggest that the quality of mineralized tissues, rather than the size of pulp chambers, plays a critical role in bacterial penetration and the development of dental abscesses.

The second most reported dental manifestation in the included studies are DDEs, particularly quantitative defects, termed enamel hypoplasia. Tooth replicas of children with XLHR, analyzed on a microscopic level, showed some signs of irregularities in enamel structure, including crater-shaped depressions and clefts [51]. Dentin defects, by contrast, were identified on X-rays as radiolucencies indicating abnormal mineralization. In the studies that analyzed these defects [26,53,54], dental abscesses were frequently observed and were often attributed to either enamel defects or, in cases of attrition, to the poorly mineralized enamel of primary teeth. The underlying dentin structure, also poorly mineralized, often became exposed, providing an easy entry point for bacteria and leading to the formation of abscesses.

The efficacy of therapeutic interventions in addressing and preventing mineralization defects in dental hard tissues remains uncertain, particularly when compared to their well-established effects on skeletal tissues. Among the included studies, conventional treatment was most commonly employed, primarily consisting of vitamin D and phosphate supplementation [48,49,51,56,59,60]. Therapy was often initiated promptly following the diagnosis of HR in all children, making it impossible to compare outcomes, specifically dental abscesses, between those who received treatment and those who did not. Burosumab therapy has emerged as a recent treatment for XLHR in children [14], showing promising results. Burosumab has been shown to decrease the monthly incidence of dental abscesses, particularly in younger children, compared to conventional therapy. This suggests a role of the drug in restoring mineralization of dental tissues [61]. Moreover, while not statistically significant, burosumab therapy demonstrated some improvement in restoring the pulp/tooth ratio, influencing the dimensions of the pulp chamber. However, the limited duration of follow-up in some studies investigating its role in preventing dental complications raises questions about its long-term effectiveness, leaving uncertainty about its lasting impact on dental tissues.

Meta-analysis revealed that the pooled prevalence of dental abscesses is higher in adults than in children, likely due to differences in dental evaluation methods. In adults, evaluations relied solely on dental history and clinical records, capturing longer periods of experience with dental abscesses.

Meta-regression analysis showed no association between therapy duration and the prevalence of dental abscesses in children. However, meta-regression for mean age at examination revealed a negative association, indicating a decrease in dental abscess prevalence with increasing age. This trend may be explained by the higher prevalence in younger children, as deciduous teeth are more susceptible [40]. As children transition to mixed and permanent dentition, the likelihood of abscesses affecting permanent teeth decreases.

One noteworthy observation that emerges from this review is the inconsistency in the classification of rickets. A significant problem that should be addressed is the use of multiple classifications HR. For example, one study referred to HR using the obsolete term “vitamin D-resistant rickets” [53], while other studies simply used HR without specifying the subtypes analyzed. Most studies, however, specifically included patients with XLHR.

Most studies indicated either a high or moderate risk of bias, primarily due to the lack of definition of potential confounding factors, such as age at therapy initiation, duration of therapy, and, in some cases, the type of treatment administered. Therefore, the results and conclusions from the present review should be interpreted with caution. However, the funnel plot and Egger test support the reliability of the meta-analysis findings on the prevalence of abscesses in children, as there is no evidence to suggest systematic bias introduced by smaller studies.

Recognizing the dental complications and the high risk of developing dental abscesses in children with HR is paramount for dentists to provide effective and early treatment. Treatment options of dental abscesses include prophylactic endodontic treatment and stainless steel crowns [63]. Pulpotomy of the deciduous teeth with formocresol solution followed by stainless steel crowns has been suggested with moderate success rates, which tend to be higher when treatment is started immediately after eruption [64]. For permanent dentition, apexification with calcium hydroxide of teeth that present necrosis without overt clinical signs of dental abscess was reported [65].

Moreover, this awareness can guide the implementation of treatment plans and tailored follow-up protocols, which may involve increased monitoring frequency.

Additionally, recognizing the heightened susceptibility of children to dental abscesses underscores the need for practitioners to provide rigorous oral hygiene instructions, emphasizing preventive measures and fostering better oral care practices.

### 4.2. Strengths and Limitations

To the best of the authors’ knowledge, this review is the first in the literature to examine the dental manifestations in children with HR. A comparative analysis of the prevalence of various dental outcomes was conducted in children and, when possible, compared to healthy children or adult populations with HR. Additionally, a meta-regression was performed to investigate the potential influence of age and duration of therapy on the prevalence of these dental manifestations.

However, several limitations were encountered. First, the included studies exhib-ited substantial heterogeneity in the method of dental evaluation and outcome report-ing, which may limit the generalizability of the findings. Second, the risk of bias was frequently high or unclear, particularly regarding confounding factors such as age at therapy initiation and duration of treatment. Third, the majority of the included stud-ies focused on XLHR, with limited data available for other forms of HR, as most studies on children with HR did not specify the subtype assessed. Regarding therapy, the effects of burosumab are relatively understudied, given its recent approval [16], and long-term data on its impact on dental health are still lacking. Finally, comparative analyses between primary and permanent dentition were not possible due to insufficient data.

## 5. Conclusions

This review offers an overview of dental manifestations in children with HR, highlighting the high prevalence of dental abscesses, DDEs, and dentin defects. Although abscesses represent the most frequent dental manifestation, especially among younger subjects, the different methods used in different studies to evaluate them make it difficult to assume an actual prevalence.

Dental practitioners may benefit from the reported results in managing the dental manifestations of the disease. However, more studies are needed to establish guidelines for the dental management of children with HR. The findings underscore the importance of a multidisciplinary team for the effective management of these patients.

Moreover, rigorous standardized studies are needed to better understand the impact of mineralization defects on dental health and to evaluate the long-term efficacy of emerging therapies such as burosumab.

## Figures and Tables

**Table 1:** Main characteristics of the included studies.

Authors	Year	Journal	Country	Type of Study	Funding Source
Ariceta et al. [26]	2023	Orphanet Journal of Rare Diseases	Multicentric	Retrospective	For-profit
Baroncelli et al. [49]	2006	European Journal of Paediatric Dentistry	Italy	Case series	Not reported
Baroncelli et al. [48]	2021	Journal of Bone and Mineral Metabolism	Italy	Case–control	No funding received
Brener et al. [50]	2022	Frontiers in Endocrinology (Lausanne)	Israel	Prospective	No funding received
Cremonesi et al. [51]	2014	Scanning	Italy	Case–control	Government
Gadion et al. [52]	2022	JBMR Plus	France	Retrospective	Not reported
Goodman et al. [53]	1998	International Journal of Paediatric Dentistry	UK	Case series	Not reported
Guevara-Canales et al. [54]	2020	Journal of Oral Research	Peru	Retrospective	Self-funded
Ito et al. [55]	2022	Endocrine Journal	Japan and South Korea	Cross-sectional survey	For-profit
Marin et al. [56]	2021	Calcified Tissue International	Chile	Cross-sectional	For-profit
Okawa et al. [57]	2024	PLoS ONE	Japan	Case–control	Government
Rabbani et al. [62]	2012	Iranian Journal of Pediatrics	Iran	Cross-sectional	Not reported
Seow and Latham [58]	1986	Pediatric Dentistry	USA	Case series	Not reported
Seow et al. [59]	1995	Pediatric Dentistry	USA	Controlled longitudinal	Not reported
Uday et al. [60]	2021	Bone	UK	Retrospective	For-profit
Ward et al. [61]	2022	Journal of Clinical Endocrinology and Metabolism	Multicentric	Randomized controlled trial	Government and for-profit

**Table 2:** Main findings of the included studies.

Authors, Year	Population	Sample	Rickets Type	Therapy (%)	Type of Dental Evaluation	Finding	
**Ariceta et al., 2023 [26]**	Children and Adults	N subjects: 579	XLHR	CT (36.79); Burosumab (30.74); No therapy (1.39); N.A. (31.08)	CR; DH	**Dental abscess/fistula (%)**	33.81
		(349 assessed for dental evaluation)				Children	22.06
						Adults	11.75
		Ch/Ad: 239/110				**Dental caries (%)**	12.89
						Children	8.59
		Mean age:				Adults	4.30
		21.70 ± 4.50 yy				**Dental anomalies (%)**	
						Taurodontism	0.85
		M/F: 205/374				Children	0.57
						Adults	0.28
						Radiolucent dentin/dentino-enamel junction	1.81
						Children	0.00
						Adults	1.81
						**Periodontal status**	
						Gingivitis (%)	1.43
						Children	1.14
						Adults	0.29
						Periodontitis (%)	2.58
						Children	0.00
						Adults	2.58
						**Endodontic status (%)**	
						Root canal surgery	4.01
						Children	0.86
						Adults	3.15
**Baroncelli et al., 2006 [49]**	Children	N subjects: 9	XLHR	CT (77.78); no therapy (22.22)	CE; DH (for spontaneous abscess or fistula); XR (only for 6 patients)	**Dental abscess/fistula (%)**	66.66 (all received CT)

		Mean age: 7.20 ± 3.30 yy					
		Age range: 2.00–13.30 yy				**Caries assessment**	
						Dental caries (%)	66.66 (all received CT)

		M/F: 3/6					
						Mean dmft/DMFT	2.66
						**Dental anomalies (%)**	
						DDEs	22.22
						Subjects received CT	28.57
						Enamel hypoplasia	11.11
						Subjects received CT	14.28
						Enamel dyschromic alterations	22.22
						Subjects received CT	28.57
**Baroncelli et al., 2021 [48]**	Children	N subjects: 47	XLHR	CT	CE; DH (for spon- taneous abscess or fistula); XR	**Dental abscess/fistula (%)**	66.66
		Exp/Cmp: 23/24				Incisors	78.03
						Canines	16.67
		Mean age:				First molars	5.30
		5.80 ± 1.60 yy				**Dental anomalies (%)**	
						Pulp chamber size Exp vs. Cmp (PCA/TA 81-63-75-36; PCH/PCW 75-36; FPAD/FTCD 75-36)	*p* < 0.01
		M/F: 20/27					
**Brener et al., 2022 [50]**	Children	N subjects: 20	XLHR	CT (up to baseline) + Burosumab (from baseline for 3 years)	DH (for abscess); XR for pulp ratio analysis	**Dental abscess/fistula (%)**	
		Exp/Cmp: 10/10				Exp	
						Up to baseline	50.00
		Mean age:				Baseline	30.00
		8.80 yy				1 yy follow-up	10.00
		Age range:				3 yy follow-up	10.00
		4.30–15.00 yy				Cmp	n.a.
						**Dental anomalies**	
		M/F (Exp): 4/6				Pulpal coronal height ratio	
						Exp	
						Baseline	0.32 (±0.07)
						1 yy follow-up	0.33 (±0.08)
						3 yy follow-up	0.29 (±0.05)
						Cmp	0.22
						Pulpal coronal width ratio	
						Exp	
						Baseline	0.48 (±0.11)
						1 yy follow-up	0.45 (±0.11)
						3 yy follow-up	0.40 (±0.11)
						Cmp	0.38
**Cremonesi et al., 2014 [51]**	Children	N subjects: 16	XLHR	CT	CE; XR (for 7 subjects); analysis of replicas by SEM	**Dental abscess/fistula** (%)	
		Exp/Cmp: 10/6				Exp	10.00
						Cmp	n.a.
		Mean age: 9.00 yy				**Dental anomalies**	
						Enamel structural alterations	
		Age range: 4.00–18.00 yy				Exp	100.00
						Cmp	0.00
						Large pulp chamber	
		M/F (Exp): 2/8				Exp	28.57
						Cmp	n.a.
						**Endodontic status**	
						Periapical radiolucency	
						Exp (7 subjects)	28.57
						Cmp (4 subjects)	25.00
**Gadion et al., 2022 [52]**	Children	N subjects: 71	XLHR	CT (53.52); Burosumab (46.48)	CR	**Dental abscess/fistula**	
						Incidence (%)	40.80
		Mean age: 7.86 ± 3.76 yy				N abscess/month	0.03 ± 0.04
						CT	0.04 ± 0.05
						Burosumab	0.01 ± 0.03
		M/F: 30/41				**Caries assessment**	
						Incidence (%)	18.30
						CT	18.40
						Burosumab	18.20
**Goodman et al., 1998 [53]**	Children	N subjects: 16	HR	N.A.	CE; XR	**Dental anomalies**	
		Ch/Ad:16				DDEs (%)	43.75
						Enlarged pulp chamber and high pulp horns (%)	
		Mean age: 6.83 yy					62.25
						Primary teeth (n)	6
		Age range: 2.08–15.7 yy				Permanent teeth (n)	5
						Hypomineralized dentin (%)	12.50
						Primary teeth (n)	2
		M/F: 14/3				Permanent teeth (n)	1
						Taurodontism (%)	43.75
**Guevara-Canales et al., 2020 [54]**	Children	N subjects: 10	HR	N.A.	CR	**Dental abscess/fistula** (%)	
						Dental abscess with fistula	10.00
		Age: n.a.				Irreversible pulpitis	40.00
						Reversible pulpitis	20.00
		M/F: 6/4				**Caries assessment**	
						Dental caries (%)	90.00
						**Dental anomalies** (%)	
						Enamel hypoplasia	20.00
						Thin dentin	20.00
						Enlarged pulp chamber	10.00
						Short malformed roots	10.00
						**Periodontal status** (%)	
						Gingivitis	70.00
						Periodontitis	20.00
**Ito et al., 2022 [55]**	Children and Adults	N subjects: 46	XLHR	CT (84.78); Burosumab (10.87); No therapy (4.35)	QST	**Dental abscess/fistula** (%)	19.57
		Ch/Ad: 14/32				Children	14.30
						Adults	21.90
		Age range: 3.00–71.00 yy				**Caries assessment**	34.78
						Dental caries (%)	
						Children	28.60
		M/F: 19/27				Adults	37.50
**Marin et al., 2021 [56]**	Children and Adults	N subjects: 26	HR	CT (92.30); No therapy (7.70)	CE; XR; DH	**Dental abscess/fistula** (%)	
		Ch/Ad: 6/20				DH	
						Adults (19 subjects)	89.00
		Age range: 5.00–64.00 yy				Children	n.a.
						CE	
						Adults (19 subjects)	35.00
		M/F: 8/18				Children (6 subjects)	33.00
						Spontaneous in children	16.67
						**Caries assessment**	
						Dental caries (%)	
						Adults	n.a.
						Children	33.33
						Mean DMFT	
						Adults	17.5 ± 9.3
						Children	2.6± 2.5
						Mean dmft (children)	2.8 ± 3.0
						**Dental anomalies**	
						Enlargement of pulp chamber	
						Adults (19 subjects)	94.73
						Children	n.a.
						**Periodontal status**	
						Gingivitis	
						Adults (19 subjects)	25.00
						Children	n.a.
						Periodontitis	
						Adults (19 subjects)	70.00
						Children	n.a.
						**Endodontic status**	
						Periapical radiolucency	
						Adults (19 subjects)	73.68
						Children	n.a.
						Endodontic treatment DH	
						Adults (19 subjects)	73.68
						Children	n.a.
**Okawa et al., 2024 [57]**	Children	N subjects: 217	XLHR	CT (35.29); Burosumab (11.76); CT + Burosumab (52.95)	CR; XR	**Dental abscess/fistula** (%)	
		Exp/Cmp: 17/200				Exp	58.80
						Cmp	n.a.
		Age: n.a.				**Dental anomalies**	
						Pulp/tooth ratio Exp vs. Cmp	
		M/F (Exp): 8/9				2–4 y	*p* = 0.63
						5–6 y	*p* = 0.02
						8–10 y	*p* = 0.56
						11–13 y	*p* = 0.10
**Rabbani et al., 2012 [62]**	Children	N subjects: 19	HR	N.A.	CE	**Dental anomalies** (%)	
						Enamel hypoplasia	42.10
		Mean age: 10.00 ± 4.23 yy				Taurodontism	15.80
						Delayed eruption	47.00
		Age range: 3.00–17.00 yy				**Periodontal status** (%)	
						Gingivitis	10.50
							
		M/F: 8/11					
**Seow & Latham, 1986 [58]**	Children and Adults	N subjects: 13	VDRR	CT	CE; DH; XR	**Dental abscess/fistula** (%)	46.15
		Children: 8/5				Children	37.50
						Adults	60.00
		Mean age: 17.00 yy				**Dental anomalies** (%)	
						Increased size of pulp chamber (12 subjects)	
		Age range: 2.00–35.00 yy					58.33
						Children (7 subjects)	42.86
						Adults (5 subjects)	60.00
		M/F: 4/9				Hypoplasia	46.15
						Children	50.00
						Adults	40.00
**Seow et al., 1995 [59]**	Children	N subjects: 13	XLHR	N.A.	CE; XR	**Dental anomalies**	
		Exp/Cmp: 19/38				Ectopic eruption of permanent canines (%)	
						
						Exp	21.00
						Cmp	5.20
		M/F (Exp): 6/13				Taurodontism (n of teeth)	
						Exp	12
						Cmp	0
**Uday et al., 2021 [60]**	Children	N subjects: 38	XLHR	CT	CR	**Dental abscess/fistula** (%)	31.50
						Recurrent	15.75
		Mean age: 9.00 yy					
						
		Age range: 3.30–18.90 yy					
						
							
		M/F: 12/26					
**Ward et al., 2022 [61]**	Children	N subjects: 61	XLHR	CT (52.46); Burosumab (47.54)	N.A	**Dental abscess/fistula** (Incidence)	
		<5 yrs: 26 (Burosumab: 14; CT: 12)				<5 yy (Incidence)	11.53
						Burosumab	0.00
						CT	25.00
		≥5 yrs: 35 (Burosumab: 15; CT: 20)				≥5 yy (Incidence)	22.85
						Burosumab	53.33
						CT	0.00
						**Caries assessment**	
		Age range: 1.00–12.00 yy				<5 yy (Incidence)	19.23
						Burosumab	35.71
						CT	0.00
		M/F: 27/34				≥5 yy (Incidence)	17.14
						Burosumab	26.66
						CT	10.00

N: number; Ch: children; Ad: adults; M: male; F: female; yy: years; Exp: exposure; Cmp: comparison; XLHR: X-linked hypophosphatemic rickets; HR: hypophosphatemic rickets; VDRR: vitamin D-resistant rickets; CT: conventional therapy; DH: dental history; CR: clinical records; CE: clinical examination; XR: X-ray; QST: questionnaire; DDEs: developmental defects of enamel; dmft: decayed, missing, filled primary teeth; DMFT: decayed, missing, filled permanent teeth; PCA: pulp chamber area; TA: tooth area; PCH: pulp chamber height; PCW: pulp chamber width; FPAD: floor to pulp apex distance; FTCD: floor to tooth crown distance; SD: standard deviation.

## Data Availability

All data extracted, generated, or analyzed during this study are included in this article and/or displayed in the Supplementary Material files. Further information can be available upon request to the corresponding author.

## Supplementary Materials

The following supporting information can be downloaded at: https://www.mdpi.com/article/10.3390/children12020144/s1, Table S1: PRISMA checklist and PRISMA checklist for abstract; Table S2: Search strings; Table S3: Extraction from, Table S4: Records excluded at title/abstract screening, Table S5: Full text selection with reasons for exclusion, Figure S1: Forest plot of analysis based on risk of bias, Figure S2: Forest plot of analysis based on type pf rickets, Figure S3: (a) Forest plot of children with caries, (b): Forest plot of children with endodontic treatment, (c): Forest plot of children with gingivitis, (d): Forest plot of children with taurodontism, and (e): Forest plot of children with large pulp chamber.
