# Supplementary material for: Dental Manifestations in Children Affected by Hypophosphatemic Rickets: A Systematic Review and Meta-Analysis
Source: Children (Basel). 2025 Jan 27;12(2):144. doi: 10.3390/children12020144 (PMC11854695; doi:10.3390/children12020144)
Supplement: Supplementary file 1 [file children-12-00144-s001.zip › Table S5.pdf]

Table S5: Full text selection and reasons for exclusion

Title	Year	Journal	Authors	Decision	Reason for exclusion
Periapical and endodontic status of permanent teeth in patients with hypophosphatemic rickets	2012	Journal of Oral Rehabilitation	Andersen, M.G. and Beck-Nielsen, S.S. and Haubek, D. and Hintze, H. and Gjørup, H. and Poulsen, S.	Excluded	Children and adults data not stratified
The International X-Linked Hypophosphatemia (XLH) Registry: first interim analysis of baseline demographic, genetic and clinical data	2023	Orphanet J. Rare Dis.	Ariceta, G. and Beck-Nielsen, S.S. and Boot, A.M. and Brandi, M.L. and Briot, K. and de Lucas Collantes, C. and Emma, F. and Giannini, S. and Haffner, D. and Keen, R. and Levtchenko, E. and Mkitie, O. and Mughal, M.Z. and Nilsson, O. and Schnabel, D. and Tripto-Shkolnik, L. and Liu, J. and Williams, A. and Wood, S. and Zillikens, M.C.	Included	
Prevalence and pathogenesis of dental and periodontal lesions in children with X-linked hypophosphatemic rickets.	2006	European journal of paediatric dentistry.	Baroncelli, G.I. and Angiolini, M. and Ninni, E. and Galli, V. and Saggese, R. and Giuca, M.R.	Included	
Pulp chamber features, prevalence of abscesses, disease severity, and PHEX mutation in X-linked hypophosphatemic rickets	2021	J. Bone Miner. Metab.	Baroncelli, G.I. and Zampollo, E. and Manca, M. and Toschi, B. and Bertelloni, S. and Michelucci, A. and Isola, A. and Bulleri, A. and Peroni, D. and Giuca, M.R.	Included	
Phenotype presentation of hypophosphatemic rickets in adults	2010	Calcified Tissue International	Beck-Nielsen, S.S. and Brusgaard, K. and Rasmussen, L.M. and Brixen, K. and Brock-Jacobsen, B. and Poulsen, M.R. and Vestergaard, P. and Ralston, S.H. and Albagha, O.M.E. and Poulsen, S. and Haubek, D. and Gjoørup, H. and Hintze, H. and Andersen, M.G. and Heickendorff, L. and Hjelmborg, J. and Gram, J.	Excluded	Only adult subjects

Dental observations in vitamin D-resistant rickets with special reference to periapical lesions	1985	Journal of Endodontics	Bender, I.B. and Naidorf, I.J.	Excluded	Study design
Hypophosphataemic rickets/osteomalacia: A descriptive analysis	2010	Indian Journal of Medical Research	Bhadada, S.K. and Bhansali, A. and Upreti, V. and Dutta, P. and Santosh, R. and Das, S. and Nahar, U.	Excluded	Outcome not specified
Dental health of pediatric patients with X-linked hypophosphatemia (XLH) after three years of burosumab therapy	2022	Frontiers in Endocrinology	Brener, R. and Zeitlin, L. and Lebenthal, Y. and Brener, A.	Included	
X-linked hypophosphatemic osteomalacia (XLH): Study of 5 adult patients	2023	Medicina Clinica	Chacur, C. and Gonzalez, E. and Peris, P.	Excluded	Outcome not specified
Dental abnormalities in patients with familial hypophosphatemic vitamin D-resistant rickets: Prevention by early treatment with 1-hydroxyvitamin D	2003	Journal of Pediatrics	Chaussain-Miller, C. and Sinding, C. and Wolikow, M. and Lasfargues, J.-J. and Godeau, G. and Garabédian, M.	Excluded	Children and adults data not stratified
Outcome of adult patients with X-linked hypophosphatemia caused by PHEX gene mutations	2018	Journal of Inherited Metabolic Disease	Chesher, D. and Oddy, M. and Darbar, U. and Sayal, P. and Casey, A. and Ryan, A. and Sechi, A. and Simister, C. and Waters, A. and Wedatilake, Y. and Lachmann, R.H. and Murphy, E.	Excluded	Only adult subjects
Patient-Reported Complications, Symptoms, and Experiences of Living with X-Linked Hypophosphatemia across the Life-Course	2021	J. Endocr. Soc.	Cheung, M. and Rylands, A.J. and Williams, A. and Bailey, K. and Bubbear, J.	Excluded	Outcome not specified
Conventional therapy in adults with x-linked hypophosphatemia: Effects on enthesopathy and dental disease	2015	Journal of Clinical Endocrinology and Metabolism	Connor, J. and Olear, E.A. and Insogna, K.L. and Katz, L. and Baker, S. and Kaur, R. and Simpson, C.A. and Sterpka, J. and Dubrow, R. and Zhang, J.H. and Carpenter, T.O.	Excluded	Only adult subjects
X-linked hypophosphatemic rickets: Enamel abnormalities and oral clinical findings	2014	Scanning	Cremonesi, I. and Nucci, C. and D'Alessandro, G. and Alkhamis, N. and Marchionni, S. and Piana, G.	Included	

X-Linked Hypophosphatemia Caused by the Prevailing North American PHEX Variant c.*231A>G; Exon 13–15 Duplication Is Often Misdiagnosed as Ankylosing Spondylitis and Manifests in Both Men and Women	2022	JBMR Plus	Dahir, K.M. and Black, M. and Gottesman, G.S. and Imel, E.A. and Mumm, S. and Nichols, C.M. and Whyte, M.P.	Excluded	Outcome not specified
Rare Diseases That Impersonate One Another: X-Linked Hypophosphatemia and Tumor-Induced Osteomalacia, a Retrospective Analysis of Discriminating Features	2022	JBMR Plus	DeCorte, J. and Randazzo, E. and Black, M. and Hendrickson, C. and Dahir, K.	Excluded	Outcome not specified
X-linked hypophosphatemic rickets: An Italian experts' opinion survey	2019	Italian Journal of Pediatrics	Emma, F. and Cappa, M. and Antoniazzi, F. and Bianchi, M.L. and Chiodini, I. and Eller Vainicher, C. and Di Iorgi, N. and Maghnie, M. and Cassio, A. and Balsamo, A. and Baronio, F. and De Sanctis, L. and Tessaris, D. and Baroncelli, G.I. and Mora, S. and Brandi, M.L. and Weber, G. and D'Ausilio, A. and Lanati, E.P.	Excluded	Children and adults data not stratified
Burosumab and Dental Abscesses in Children With X-Linked Hypophosphatemia	2022	JBMR Plus	Gadion, M. and Hervé, A. and Herrou, J. and Rothenbuhler, A. and Smail-Faugeron, V. and Courson, F. and Linglart, A. and Chaussain, C. and Biosse Duplan, M.	Included	
Dental problems associated with hypophosphataemic vitamin D resistant rickets	1998	International Journal of Paediatric Dentistry	Goodman, J.R. and Gelbier, M.J. and Bennett, J.H. and Winter, G.B.	Included	
Prevalence of oral manifestations of hypophosphatemic rickets in patients treated in a Peruvian pediatric hospital	2020	Journal of Oral Research	Guevara-Canales, J.O. and Cardenas-Zuñiga, N. and Chavez-Pastor, M. and Trubnykova, M. and Morales-Vadillo, R.	Included	
Development of Spinal Enthesopathies in Adults With X-linked Hypophosphatemia	2023	J. Clin. Endocrinol. Metab.	Herrou, J. and Fechtenbaum, J. and Rothenbuhler, A. and Kamenický, P. and Roux, C. and	Excluded	Outcome not specified

			Linglart, A. and Briot, K.		
Improved Oral Health in Adults with X-Linked Hypophosphatemia Treated with Burosumab	2024	J Clin Endocrinol Metab	Hervé, A. and Gadion, M. and Herrou, J. and Izart, M. and Linglart, A. and Cohen-Solal, M. and Lecoq, A.-L. and Kamenicky, P. and Briot, K. and Chaussain, C. and Biosse Duplan, M.	Excluded	Only adult subjects
Burden of disease of X-linked hypophosphatemia in Japanese and Korean patients: a cross-sectional survey	2022	Endocr. J.	Ito, N. and Kang, H.G. and Nishida, Y. and Evins, A. and Skrinar, A. and Cheong, H.I.	Included	
Early Discrimination Between Tumor-Induced Rickets/Osteomalacia and X-Linked Hypophosphatemia in Chinese Children and Adolescents: A Retrospective Case–Control Study	2021	Journal of Bone and Mineral Research	Jiajue, R. and Ni, X. and Jin, C. and Huo, L. and Wu, H. and Liu, Y. and Jin, J. and Yu, W. and Lv, W. and Zhou, L. and Xia, Y. and Chi, Y. and Cui, L. and Pang, Q. and Li, X. and Jiang, Y. and Wang, O. and Li, M. and Xing, X. and Meng, X. and Xia, W.	Excluded	Outcome not specified
Effect of Conventional Treatment on Dental Complications and Ectopic Ossifications Among 30 Adults With XLH	2023	Journal of Clinical Endocrinology and Metabolism	Kato, H. and Okawa, R. and Ogasawara, T. and Hoshino, Y. and Hidaka, N. and Koga, M. and Kinoshita, Y. and Kobayashi, H. and Taniguchi, Y. and Fukumoto, S. and Nangaku, M. and Makita, N. and Hoshi, K. and Nakano, K. and Ito, N.	Excluded	Outcome not specified
Frequency and clinical profile of hypophosphatemic rickets among rachitic children	2018	Medical Forum Monthly	Laghari, T.M. and Ashfaq, M. and Ali, S.	Excluded	Outcome not specified
Dental health of patients with X-linked hypophosphatemia: A controlled study	2023	Frontiers in Oral Health	Larsson, A. and Regnstrand, T. and Skott, P. and Mäkitie, O. and Björnsdottir, S. and Garming-Legert, K.	Excluded	Only adult subjects
Analysis of mineral density of calcified tissues in children with x-linked hypophosphatemic rickets	2021	International Journal of Biomedicine	Lezhnev, D.A. and Vislobokova, E.V. and Kiselnikova, L.P. and Sholokhova,	Excluded	Different outcome

and hypophosphatasia using cone beam computed tomography data			N.A. and Smyslenova, M.V. and Truten, V.P.		
Characterization of Oral Health Status in Chilean Patients with X-Linked Hypophosphatemia	2021	Calcif. Tissue Int.	Marin, A. and Morales, P. and Jiménez, M. and Borja, E. and Ivanovic-Zuvic, D. and Collins, M.T. and Florenzano, P.	Included	
Prevalence of dental abscess in a population of children with vitamin D-resistant rickets.	1991	Pediatric dentistry	McWhorter, A.G. and Seale, N.S.	Excluded	Children and adults data not stratified
Evaluation of dental manifestations in X-linked hypophosphatemia using orthopantomography	2024	PLoS ONE	Okawa, R. and Takagi, M. and Nakamoto, T. and Kakimoto, N. and Nakano, K.	Included	
X-linked hypophosphatemia in Polish patients. 2. Analysis of clinical features and genotype-phenotype correlation	2001	Journal of Applied Genetics	Popowska, E. and Pronicka, E. and Sułek, A. and Jurkiewicz, D. and Rowińska, E. and Sykut-Cegielska, J. and Rump, Z. and Arasimowicz, E. and Krajewska-Walasek, M.	Excluded	Outcome not specified
Dental problems in calcium metabolism disorders	2011	Tehran University Medical Journal	Rabbani, A. and Rahmani, P. and Qoddosi, S. and Ziyaee, V.	Excluded	Full text not available in English
Dental problems in hypophosphatemic rickets, a cross sectional study	2012	Iranian Journal of Pediatrics	Rabbani, A. and Rahmani, P. and Ziaee, V. and Ghodoosi, S.	Included	
X-linked hypophosphatemia: a clinical, biochemical, and histopathologic assessment of morbidity in adults.	1989	Medicine	Reid IR and Hardy DC and Murphy WA and Teitelbaum SL and Bergfeld MA and Whyte MP	Excluded	Only adult subjects
Molecular Diagnoses of X-Linked and Other Genetic Hypophosphatemias: Results From a Sponsored Genetic Testing Program	2022	J. Bone Miner. Res.	Rush, E.T. and Johnson, B. and Aradhya, S. and Beltran, D. and Bristow, S.L. and Eisenbeis, S. and Guerra, N.E. and Krolczyk, S. and Miller, N. and Morales, A. and Ramesan, P. and Sarafrazi, S. and Truty, R. and Dahir, K.	Excluded	Outcome not specified

Prevalence and characteristics of paediatric X-linked hypophosphataemia in Australia and New Zealand: Results from the Australian and the New Zealand Paediatric Surveillance Units survey	2023	Bone	Sandy, J.L. and Nunez, C. and Wheeler, B.J. and Jefferies, C. and Morris, A. and Siafarikas, A. and Rodda, C.P. and Simm, P. and Biggin, A. and Aum, S. and Elliot, E.J. and Munns, C.F.	Excluded	Study design
Oral findings in patients with autosomal dominant hypophosphatemic bone disease and X-linked hypophosphatemia: Further evidence that they are different diseases	1988	Oral Surgery, Oral Medicine, Oral Pathology	Schwartz, S. and Scriver, C.R. and Reade, T.M. and Shields, E.D.	Excluded	Children and adults data not stratified
The effect of medical therapy on dentin formation in vitamin D-resistant rickets.	1991	Pediatric dentistry	Seow, W.K.	Excluded	Study design
The spectrum of dental manifestations in vitamin D-resistant rickets: implications for management.	1986	Pediatric dentistry	Seow, W.K. and Latham, S.C.	Included	
Effect of familial hypophosphatemic rickets on dental development: a controlled, longitudinal study.	1995	Pediatric dentistry	Seow, W.K. and Needleman, H.L. and Holm, I.A.	Included	
Evaluation of aggressive pulp therapy in a population of vitamin D-resistant rickets patients: a follow-up of 4 cases.	2002	Pediatric dentistry	Shroff, D.V. and McWhorter, A.G. and Seale, N.S.	Excluded	Different outcome
Dental abnormalities and oral health in patients with hypophosphatemic rickets	2010	Clinics	Souza, M.A. and Soares, L.A.V. and Santos, M.A.D. and Vaisbich, M.H.	Excluded	Children and adults data not stratified
Monitoring response to conventional treatment in children with XLH: Value of ALP and Rickets Severity Score (RSS) in a real world setting	2021	Bone	Uday, S. and Shaw, N.J. and Mughal, M.Z. and Randell, T. and Högler, W. and Santos, R. and Padidela, R.	Included	
Effect of Burosumab Compared With Conventional Therapy on Younger vs Older Children With X-linked Hypophosphatemia	2022	Journal of Clinical Endocrinology and Metabolism	Ward, L.M. and Glorieux, F.H. and Whyte, M.P. and Munns, C.F. and Portale, A.A. and Hogler, W. and Simmons, J.H. and Gottesman, G.S. and Padidela, R. and Namba, N. and Cheong, H.I. and Nilsson, O. and Mao, M. and Chen, A. and	Included	

			Skrinar, A. and Roberts, M.S. and Imel, E.A.		
Periodontal status of patients with hypophosphatemic rickets: A case series	2011	Journal of Periodontology	Ye, L. and Liu, R. and White, N. and Alon, U.S. and Cobb, C.M.	Excluded	Only adult subjects
