# Supplementary material for: Dental Manifestations in Children Affected by Hypophosphatemic Rickets: A Systematic Review and Meta-Analysis
Source: Children (Basel). 2025 Jan 27;12(2):144. doi: 10.3390/children12020144 (PMC11854695; doi:10.3390/children12020144)
Supplement: Supplementary file 1 [file children-12-00144-s001.zip › Figure S3 (a-e).pdf]

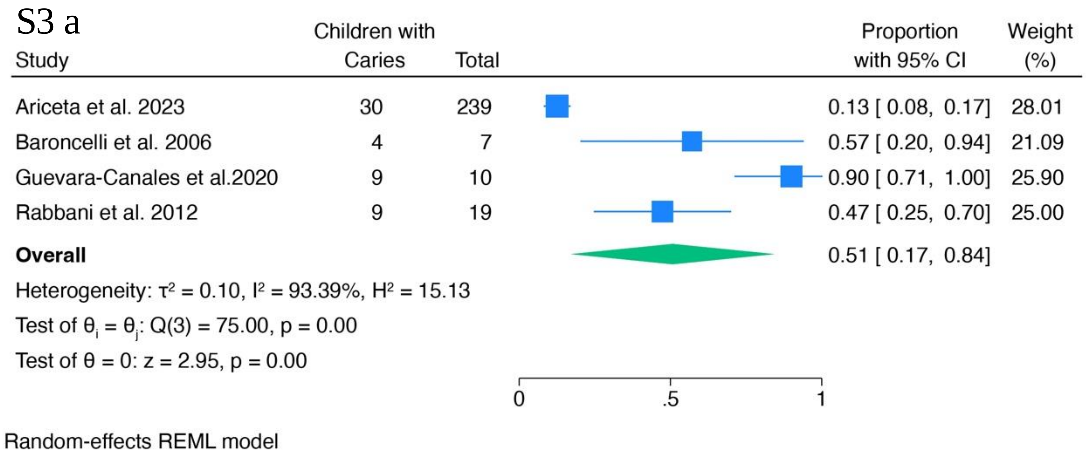

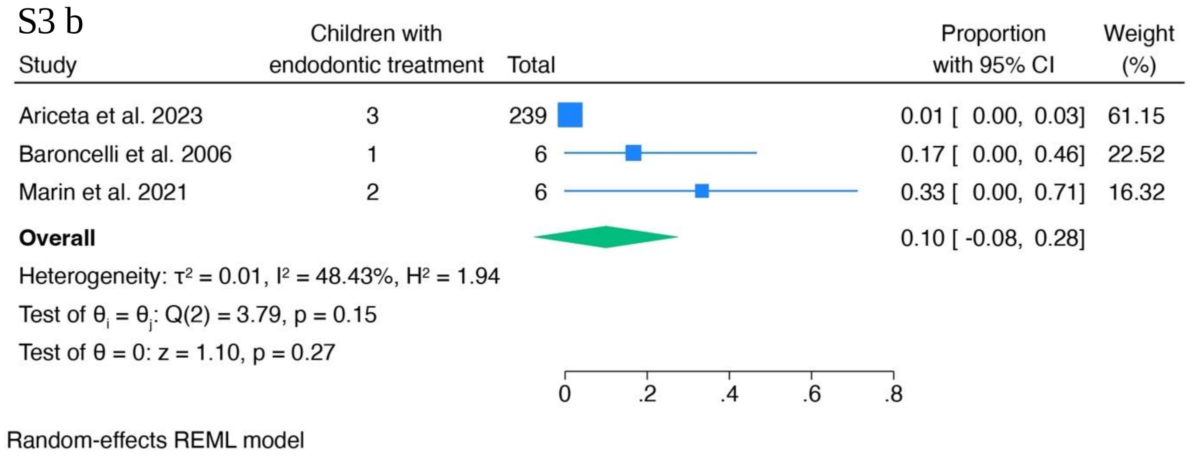

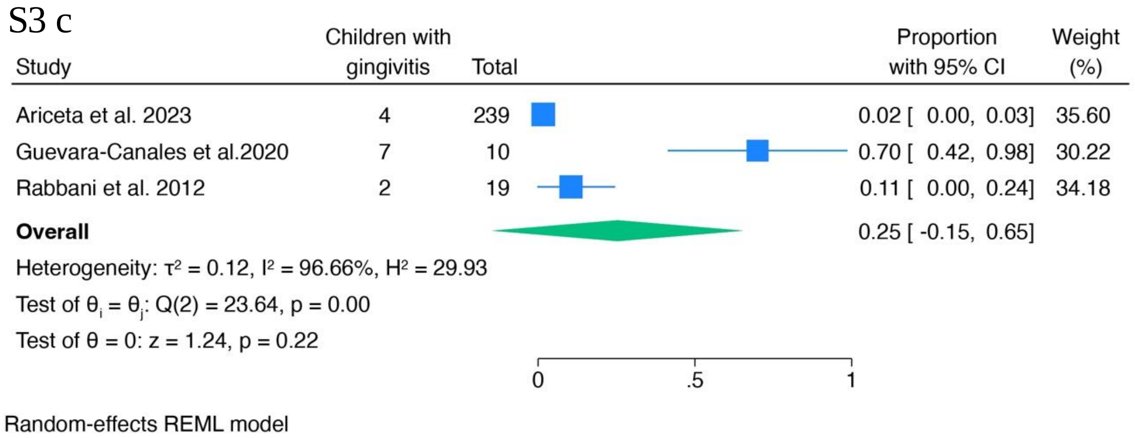

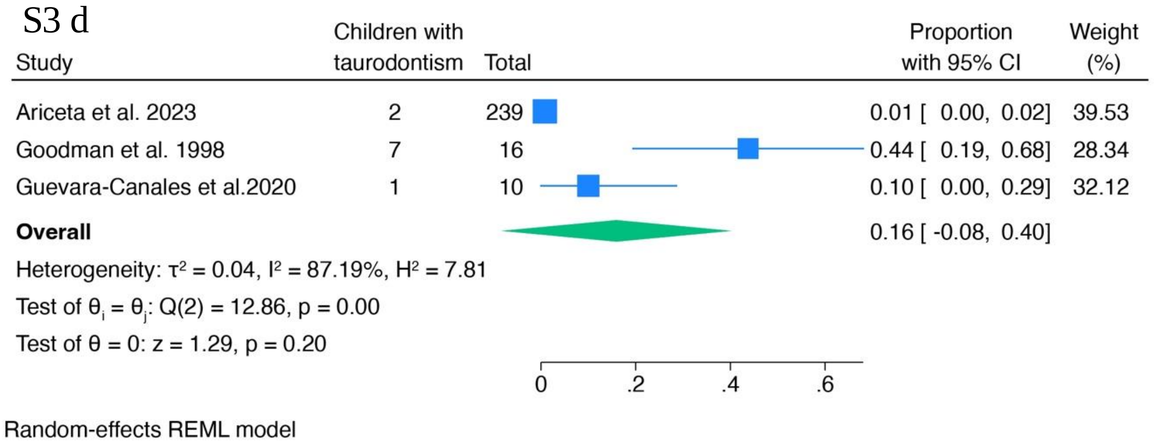

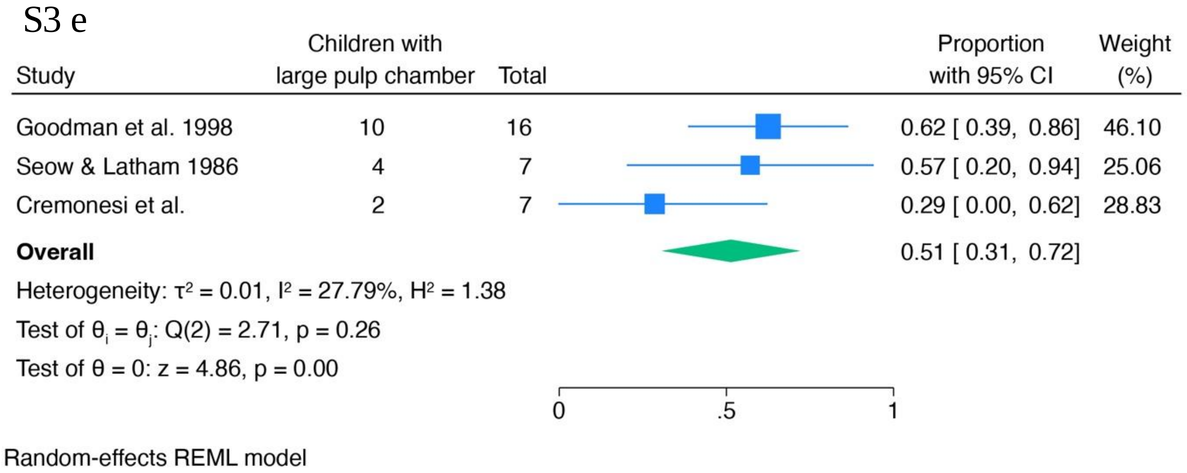

Figure S3. (S3 a): Forest plot of children with caries, (S3b): Forest plot of children with endodontic treatment, (S3 c): Forest plot of children with gingivitis, (S3 d): Forest plot of children with taurodontism, (S3 e): Forest plot of children with large pulp chamber
