# Supplementary material for: Dental Manifestations in Children Affected by Hypophosphatemic Rickets: A Systematic Review and Meta-Analysis
Source: Children (Basel). 2025 Jan 27;12(2):144. doi: 10.3390/children12020144 (PMC11854695; doi:10.3390/children12020144)
Supplement: Supplementary file 1 [file children-12-00144-s001.zip › Table S2.pdf]

Table S2: Search strings used for each database

PubMed	*((″Vitamin D-Resistant Rickets″) OR (″Familial Hypophosphatemic Rickets″) OR (″Hereditary hypophosphatemia″) OR (″Hereditary hypophosphatemic rickets″) OR (″X-linked hypophosphatemia″) OR (“Hypophosphatemic Rickets”) OR (″Familial Hypophosphatemic Rickets″[MeSH Terms])) AND ((Dent*) OR (tooth) OR (teeth) OR (Dental defect) OR (Tooth Abnormalities[MeSH Terms])) NOT ((review[Title]) OR (″systematic review″[Title]) OR (″meta-analysis″[Title]))*
Embase	*′Vitamin D-Resistant Rickets′/exp OR ′Familial Hypophosphatemic Rickets′/exp OR ′Hereditary hypophosphatemia′/exp OR ′Hereditary hypophosphatemic rickets′/exp OR ′X-linked hypophosphatemia′/exp OR ′Hypophosphatemic Rickets′/exp AND ′Dent*′/exp OR ′tooth′/exp OR ′teeth′/exp OR ′Dental defect*′/exp OR ′Tooth Abnormalities′/exp NOT ′review′:ab,ti OR ′systematic review′:ab,ti OR ′meta-analysis′:ab,ti*
Scopus	*TITLE-ABS-KEY(“vitamin d-resistant rickets”) OR TITLE-ABS-KEY(“familial hypophosphatemic rickets”) OR TITLE-ABS-KEY(“hereditary hypophosphatemia”) OR TITLE-ABS-KEY(“hereditary hypophosphatemic rickets”) OR TITLE-ABS-KEY(“′X-linked hypophosphatemia”) OR TITLE-ABS-KEY(“Hypophosphatemic Rickets”) AND TITLE-ABS-KEY(“Dent*”) OR TITLE-ABS-KEY(“tooth”) OR ALL(“teeth”) OR TITLE-ABS-KEY(“Dental defect*”) OR TITLE-ABS-KEY(“′Tooth Abnormalities”) NOT TITLE(“review”) OR TITLE(“systematic review”) OR TITLE(“meta-analysis”)*
