# Supplementary material for: Dental Manifestations in Children Affected by Hypophosphatemic Rickets: A Systematic Review and Meta-Analysis
Source: Children (Basel). 2025 Jan 27;12(2):144. doi: 10.3390/children12020144 (PMC11854695; doi:10.3390/children12020144)
Supplement: Supplementary file 1 [file children-12-00144-s001.zip › Table S3.pdf]

Table S3: Data extraction form

Population	Mean age in years	Total N subjects	N subjects REF	M/F	N subjects CTRL	N subjects dental evaluation	Type of dental evaluation	Dental abscess REF % (N)	Dental abscess CTRL % (N)	Dental caries REF % (N)	Dental caries CTRL % (N)	Mean dmft/DMFT REF	Mean dmft/DMFT CTRL	Dental anomalies REF % (N)	Dental anomalies CTRL % (N)	Periodontal problems REF % (N)	Periodontal problems CTRL % (N)	Endoodntic ttt/periapical radiolucency REF % (N)	Endoodntic ttt/periapical radiolucency CTRL % (N)	Funding
