# Supplementary material for: Dental Manifestations in Children Affected by Hypophosphatemic Rickets: A Systematic Review and Meta-Analysis
Source: Children (Basel). 2025 Jan 27;12(2):144. doi: 10.3390/children12020144 (PMC11854695; doi:10.3390/children12020144)
Supplement: Supplementary file 1 [file children-12-00144-s001.zip › Table S4.pdf]

Table S4: Excluded records at title/ abstract screening

Title	Year	Journal	Authors
Abnormal vitamin D metabolism in the X-linked hypophosphatemic mouse	1980	ENDOCRINOLOGY	Meyer Jr., R.A. and Cray, R.W. and Meyer, M.H.
Osteomalacia and weakness from excessive antacid ingestion	1980	J. AM. MED. ASSOC.	Insogna, K.L. and Bordley, D.R. and Caro, J.F. and Lockwood, D.H.
Craniometric measurements of craniofacial malformations in mice with X-linked, dominant hypophosphatemia (vitamin D-resistant rickets)	1980	Teratology	Iorio, R.J. and Murray, G. and Meyer, R.A.
Increased urinary excretion of cyclic nucleotides in X-linked hypophosphatemic (Hyp) mice	1981	EXPERIENTIA	Kiebzak, G.M. and Meyer Jr., R.A. and Mish, P.M.
Hypocalcemic, hypophosphatemic rickets in rat pups suckling vitamin D-deprived mothers	1981	ENDOCRINOLOGY	Boass, A. and Ramp, W.K. and Toverud, S.U.
The etiology of enamel hypoplasia: A unifying concept	1981	The Journal of Pediatrics	Nikiforuk, G. and Fraser, D.
In vivo response of X-linked hypophosphatemic mice to calcitonin	1982	HORM. METAB. RES.	Kiebzak, G.M. and Meyer Jr., R.A.
X-linked hypophosphatemic mice are not hypersensitive to parathyroid hormone	1982	ENDOCRINOLOGY	Kiebzak, G.M. and Meyer Jr., R.A.
Naturally-occurring exposure of the dental pulp in mice with inherited hypophosphataemia	1982	Archives of Oral Biology	Sofaer, J.A. and Southam, J.C.
A case of vitamin D resistant rickets	1982	Tandlaegebladet	Holst, E. and Pedersen, S.S.
Management of the primary dentition in vitamin D-resistant rickets	1982	Oral Surgery, Oral Medicine, Oral Pathology	Rakocz, M. and Keating III, J. and Johnson, R.
Normal milk composition in lactating X-linked hypophosphatemic mice despite continued hypophosphatemia	1983	CALCIF. TISSUE INT.	Delzer, P.R. and Meyer Jr., R.A.
Structural organization of calcospherites in normal and rachitic human dentine	1983	Archives of Oral Biology	Shellis, R.P.
Dental management of familial hypophosphatemic vitamin D-resistant rickets: report of case.	1983	ASDC journal of dentistry for children	Yasufuku, Y. and Kohno, N. and Tsutsumi, N. and Ooshima, T. and Sobue, S. and Murakami, Y. and Ikari, H.
Metabolites of vitamin D in normal and X-linked hypophosphatemic mice	1984	CALCIF. TISSUE INT.	Meyer Jr., R.A. and Meyer, M.H. and Gray, R.W.
X-linked hypophosphataemic vitamin D-resistant rickets	1984	Australian Dental Journal	Seow, W.K.
Disturbances in dental calcification in untreated and treated vitamin D-resistant rickets	1984	Minerva Pediatrica	Berio, A. and Moro, G.L.
The mechanism of end-organ resistance to 1α,25-dihydroxycholecalciferol in the common marmoset	1985	BIOCHEM. J.	Takahashi, N. and Suda, S. and Shinki, T.
A case of hypophosphatemic vitamin D-resistant rickets in which spontaneous dental abscesses were the first evidence	1985	Shoni shikagaku zasshi. The Japanese journal of pedodontics	Yamazaki, H. and Otake, Y. and Tomizawa, M. and Noda, T. and Suzuki, M.
The concurrence of hypoparathyroidism provides new insights to the pathophysiology of X-linked hypophosphatemic rickets	1985	Journal of Clinical Endocrinology and Metabolism	Lyles, K.W. and Burkes, E.J. and McNamara, C.R. and Harrelson, J.M. and Pickett, J.P. and Drezner, M.K.
Increased metabolic rate in X-linked hypophosphatemic mice	1986	ENDOCRINOLOGY	Vaughn, L.K. and Meyer Jr., R.A. and Meyer, M.H.

Clinical features of hereditary resistance to 1,25-dihydroxyvitamin D (hereditary hypocalcemic vitamin D resistant rickets type II).	1986	Advances in experimental medicine and biology	Liberman UA and Eil C and Marx SJ
Prophylactic dental treatment for a patient with vitamin D-resistant rickets: report of case.	1986	ASDC journal of dentistry for children	Breen, G.H.
Hereditary hypophosphatemia rickets: an important awareness for dentists.	1986	ASDC journal of dentistry for children	Herbert, F.L.
Rearing and step climbing behavior of X-linked hypophosphatemic (Hyp) mice	1987	PHYSIOL. BEHAV.	Vaughn, L.K. and Quock, R.M. and Meyer Jr., R.A.
Mineral uptake by the femora of older female X-linked hypophosphatemic (HYP) mice but not older male HYP mice	1987	CLIN. ORTHOP. RELAT. RES.	Brault, B.A. and Meyer, M.H. and Meyer, R.A. and Iorio, R.J.
Evidence that low plasma 1,25-dihydroxyvitamin D causes intestinal malabsorption of calcium and phosphate in juvenile X-linked hypophosphatemic mice.	1987	Journal of bone and mineral research : the official journal of the American Society for Bone and Mineral Research	Meyer RA Jr and Meyer MH and Gray RW and Bruns ME
Malabsorption of phosphate by the intestines of young X-linked hypophosphatemic mice.	1988	Calcified tissue international	Brault BA and Meyer MH and Meyer RA Jr
Structural deformities of deciduous teeth in patients with hypophosphatemic vitamin D-resistant rickets	1988	Oral Surgery, Oral Medicine, Oral Pathology	Abe, K. and Ooshima, T. and Lily, T.S.M. and Yasufuku, Y. and Sobue, S.
Parabiosis suggests a humoral factor is involved in X-linked hypophosphatemia in mice.	1989	Journal of bone and mineral research : the official journal of the American Society for Bone and Mineral Research	Meyer RA Jr and Meyer MH and Gray RW
The renal phosphate transport defect in normal mice parabiosed to X-linked hypophosphatemic mice persists after parathyroidectomy.	1989	Journal of bone and mineral research : the official journal of the American Society for Bone and Mineral Research	Meyer RA Jr and Tenenhouse HS and Meyer MH and Klugerman AH
Micromorphologic features of dentin in vitamin D-resistant rickets: correlation with clinical grading of severity.	1989	Pediatric dentistry	Seow, W.K. and Romaniuk, K. and Sclavos, S.
The crystallinity of human deciduous teeth in hypophosphataemic vitamin D-resistant rickets	1989	Archives of Oral Biology	Abe, K. and Ooshima, T. and Sobue, S. and Moriwaki, Y.
X-linked hypophosphatemia: the mutant gene is expressed in teeth as well as in kidney	1990	American Journal of Human Genetics	Shields, E.D. and Scriver, C.R. and Reade, T. and Fujiwara, T.M. and Morgan, K. and Ciampi, A. and Schwartz, S.
Dental hypersensitivity due to hypophosphataemia?	1990	Dentomaxillofacial Radiology	Hintze, H. and Wenzel, A. and Kruhöffer, F.
Craniofacial abnormalities in mice with X-linked hypophosphatemic genes (Hyp or Gy).	1991	Teratology	Shetty NS and Meyer RA Jr
Osteomalacia in hereditary hypophosphatemic rickets with hypercalciuria: a correlative clinical-histomorphometric study.	1991	The Journal of clinical endocrinology and metabolism	Gazit D and Tieder M and Liberman UA and Passi-Even L and Bab IA
[Rathbun syndrome (hypophosphatasia). Clinical aspects: dwarfism and Bechterew symptoms].	1991	Zeitschrift fur Rheumatologie	Krohn-Grimberghe B and Ludwig B and Furkert D
Rathbun-syndrome (Hypophosphatasia). Clinical: Growth retardation and symptoms of ankylosing spondylitis (Bechterew's disease)	1991	Zeitschrift fur Rheumatologie	Krohn-Grimberghe, B. and Ludwig, B. and Furkert, D.
Mineral content of different areas of human dentin in hypophosphataemic vitamin D-resistant rickets.	1991	Journal de biologie buccale	Hietala, E.L. and Larmas, M.A.

X-Ray diffraction analysis and transmission electron microscopic examination of globular dentin	1991	Calcified Tissue International	Abe, K. and Masatomi, Y. and Moriwaki, Y. and Ooshima, T.
X-linked hypophosphatemia: An appreciation of a classic paper and a survey of progress since 1958	1991	Medicine (United States)	Scriver, C.R. and Tenenhouse, H.S. and Glorielx, F.H.
Scanning electron microscopic analysis of dentin in vitamin D-resistant rickets--assessment of mineralization and correlation with clinical findings.	1991	Pediatric dentistry	Seeto, E. and Seow, W.K.
The Occurrence of Interglobular Dentin in Incisors of Hypophosphatemic Mice Fed a High-calcium and High-phosphate Diet	1992	Journal of Dental Research	Abe, K. and Masatomi, Y. and Nakajima, Y. and Shintani, S. and Moriwaki, Y. and Sobue, S. and Ooshima, T.
An unusual giant cell lesion in a child with vitamin D-resistant rickets	1992	International Journal of Paediatric Dentistry	CHADWICK, B.L. and ALDRED, M.J.
X-linked hypophosphataemia: A homologous phenotype in humans and mice with unusual organ-specific gene dosage	1992	Journal of Inherited Metabolic Disease	Scriver, C.R. and Tenenhouse, H.S.
Genetic mapping in the Xp11.2 region of a new form of X-linked hypophosphatemic rickets.	1993	European journal of human genetics : EJHG	Bolino, A. and Devoto, M. and Enia, G. and Zoccali, C. and Weissenbach, J. and Romeo, G.
Parental origin of mutant allele does not explain absence of gene dose in X-linked Hyp mice	1993	Genetical Research	Qiu, Z.Q. and Tenenhouse, H.S. and Scriver, C.R.
X-linked hypophosphatemia: molecular biology and treatment controversies.	1994	Zhonghua Minguo xiao er ke yi xue hui za zhi [Journal]. Zhonghua Minguo xiao er ke yi xue hui	Thabet MA and Truchina O and Chan JC
Phosphate transport in osteoblasts from normal and X-linked hypophosphatemic mice	1994	Calcified Tissue International	Rifas, L. and Dawson, L.L. and Halstead, L.R. and Roberts, M. and Avioli, L.V.
X-Linked hypophosphatemic rickets: a disease often unknown to affected patients	1994	Bone and Mineral	Econs, M.J. and Samsa, G.P. and Monger, M. and Drezner, M.K. and Feussner, J.R.
Recurring oral giant cell lesion in a child with X-linked hypophosphatemic rickets: Clinical manifestation of occult parathyroidism?	1995	The Journal of Pediatrics	Stratakis, C.A. and Mitsiades, N.S. and Sun, D. and Chrousos, G.P. and O'Connell, A.
A common molecular basis for three inherited kidney stone diseases	1996	Nature	Lloyd, S.E. and Pearce, S.H.S. and Fisher, S.E. and Steinmeyer, K. and Schwappach, B. and Scheinman, S.J. and Harding, B. and Bolino, A. and Devoto, M. and Goodyer, P. and Rigden, S.P.A. and Wrong, O. and Jentsch, T.J. and Craig, I.W. and Thakker, R.V.
cDNA cloning of the murine Pex gene implicated in X-linked hypophosphatemia and evidence for expression in bone	1996	Genomics	Du, L. and Desbarats, M. and Viel, J. and Glorieux, F.H. and Cawthorn, C. and Ecarot, B.

X chromosome inactivation pattern in female carriers of X linked hypophosphataemic rickets	1996	Journal of Medical Genetics	Ørstavik, K.H. and Ørstavik, R.E. and Halse, J. and Knudtzon, J.
Effects of serum phosphate level on formation of incisor dentine in hypophosphatemic mice	1996	Journal of Oral Pathology and Medicine	Masatomi, Y. and Nakagawa, Y. and Kanamoto, Y. and Sobue, S. and Ooshima, T.
Clinical course of hypophosphatemic rickets in 23 adults	1996	Clinical Nephrology	Berndt, M. and Ehrich, J.H.H. and Lazovic, D. and Zimmermann, J. and Hillmann, G. and Kayser, C. and Prokop, M. and Schirg, E. and Siegert, B. and Wolff, G. and Brodehl, J.
X-linked hypophosphatemia: A search for gender, race, anticipation, or parent of origin effects on disease expression in children	1996	Journal of Clinical Endocrinology and Metabolism	Whyte, M.P. and Schranck, F.W. and Armamento-Villareal, R.
Diagnosis of X-linked hypophosphatemic vitamin D resistant rickets.	1997	Acta paediatrica Japonica : Overseas edition	Yamamoto T
Orthodontic treatment of a patient with, hypophosphatemic vitamin D-resistant rickets	1997	Journal of Dentistry for Children	Kawakami, M. and Takano-Yamamoto, T.
Diagnosis of X-linked hypophosphatemia vitamin D resistant rickets	1997	Pediatrics International	Yamamoto, T.
New perspectives on the biology and treatment of X-linked hypophosphatemic rickets	1997	Pediatric Clinics of North America	Carpenter, T.O.
Genomic organization of the human PEX gene mutated in X-linked dominant hypophosphatemic rickets	1997	Genome Research	Francis, F. and Strom, T.M. and Hennig, S. and Böddrich, A. and Lorenz, B. and Brandau, O. and Mohnike, K.L. and Cagnoli, M. and Steffens, C. and Klages, S. and Borzym, K. and Pohl, T. and Oudet, C. and Econs, M.J. and Rowe, P.S.N. and Reinhardt, R. and Meitinger, T. and Lehrach, H.
A second family with XLRH displays the mutation S244L in the CLCN5 gene	1997	Human Genetics	Oudet, C. and Martin-Coignard, D. and Pannetier, S. and Praud, E. and Champion, G. and Hanauer, A.
Targeted inactivation of Npt2 in mice leads to severe renal phosphate wasting, hypercalciuria, and skeletal abnormalities	1998	Proc. Natl. Acad. Sci. U. S. A.	Beck, L. and Karaplis, A.C. and Amizuka, N. and Hewson, A.S. and Ozawa, H. and Tenenhouse, H.S.
Intrinsic mineralization defect in Hyp mouse osteoblasts	1998	Am. J. Physiol. Endocrinol. Metab.	Xiao, Z.S. and Crenshaw, M. and Guo, R. and Nesbitt, T. and Drezner, M.K. and Quarles, L.D.
Implant placement and guided tissue regeneration in a patient with congenital vitamin D-resistant rickets.	1998	The Journal of oral implantology	Resnick, D.
Mutations in CLCN5 chloride channel in Japanese patients with low molecular weight proteinuria	1998	Journal of the American Society of Nephrology	Morimoto, T. and Uchida, S. and Sakamoto, H. and Kondo, Y. and Hanamizu, H. and Fukui, M. and

			Tomino, Y. and Nagano, N. and Sasaki, S. and Marumo, F.
X-linked hypercalciuric nephrolithiasis: Clinical syndromes and chloride channel mutations	1998	Kidney International	Scheinman, S.J.
Pex mRNA is localized in developing mouse osteoblasts and odontoblasts	1998	Journal of Histochemistry and Cytochemistry	Ruchon, A.F. and Marcinkiewicz, M. and Siegfried, G. and Tenenhouse, H.S. and DesGroseillers, L. and Crine, P. and Boileau, G.
Evidence for Phex haploinsufficiency in murine X-linked hypophosphatemia	1999	Mammalian Genome	Wang, L. and Du, L. and Ecarot, B.
Pathogenesis of Dent's disease and related syndromes of X-linked nephrolithiasis.	2000	Kidney international	Thakker RV
Developmental expression and tissue distribution of Phex protein: Effect of the Hyp mutation and relationship to bone markers	2000	Journal of Bone and Mineral Research	Ruchon, A.F. and Tenenhouse, H.S. and Marcinkiewicz, M. and Siegfried, G. and Aubin, J.E. and Desgroseillers, L. and Crine, P. and Boileau, G.
Autosomal dominant hypophosphataemic rickets is associated with mutations in FGF23	2000	Nature Genetics	White, K.E. and Evans, W.E. and O'Riordan, J.L.H. and Speer, M.C. and Econs, M.J. and Lorenz-Depiereux, B. and Grabowski, M. and Meitinger, T. and Strom, T.M.
Familial hypophosphatemic vitamin d-resistant rickets: Dental findings and histologic study of teeth	2000	Oral Surgery, Oral Medicine, Oral Pathology, Oral Radiology, and Endodontics	Murayama, T. and Iwatsubo, R. and Akiyama, S. and Amano, A. and Morisaki, I.
PHEXdb, a locus-specific database for mutations causing X-linked hypophosphatemia	2000	Human Mutation	Sabbagh, Y. and Jones, A.O. and Tenenhouse, H.S.
[Familial hypophosphatemic rickets].	2001	Orvosi hetilap	Reusz G
[Hypophosphatemia and rickets/osteomalacia].	2001	Clinical calcium	Tanaka H
MEPE, the gene encoding a tumor-secreted protein in oncogenic hypophosphatemic osteomalacia, is expressed in bone	2001	Genomics	Argiro, L. and Desbarats, M. and Glorieux, F.H. and Ecarot, B.
Mutational analysis and genotype-phenotype correlation of the PHEX gene in X-linked hypophosphatemic rickets	2001	Journal of Clinical Endocrinology and Metabolism	Holm, I.A. and Nelson, A.E. and Robinson, B.G. and Mason, R.S. and Marsh, D.J. and Cowell, C.T. and Carpenter, T.O.
A new approach to mRNA in proximal tubule cells of patients with CLCN5 channelopathy	2001	Pediatric Nephrology	Morimoto, T. and Chiba, A. and Kondo, Y. and Takahashi, S. and Igarashi, T. and Inoue, C.N. and Iinuma, K.
Effect of growth hormone treatment on final height, phosphate metabolism, and bone mineral density in children with X-linked hypophosphatemic rickets	2001	Journal of Pediatrics	Baroncelli, G.I. and Bertelloni, S. and Ceccarelli, C. and Saggese, G.

Endodontic management of a patient with X-linked hypophosphataemic rickets	2001	Australian Endodontic Journal	Alexander, S. and Moloney, L. and Kilpatrick, N.
Ontogeny of Phex/PHEX protein expression in mouse embryo and subcellular localization in osteoblasts	2002	J. Bone Miner. Res.	Thompson, D.L. and Sabbagh, Y. and Tenenhouse, H.S. and Roche, P.C. and Drezner, M.K. and Salisbury, J.L. and Grande, J.P. and Poeschla, E.M. and Kumar, R.
Partial rescue of the Hyp phenotype by osteoblast-targeted PHEX (phosphate-regulating gene with homologies to endopeptidases on the X chromosome) expression	2002	Molecular Endocrinology	Bai, X. and Miao, D. and Panda, D. and Grady, S. and McKee, M.D. and Goltzman, D. and Karaplis, A.C.
The dentino-enamel junction revisited	2002	Connective Tissue Research	Goldberg, M. and Septier, D. and Bourd, K. and Hall, R. and Jeanny, J.-C. and Jonet, L. and Colin, S. and Tager, F. and Chaussain-Miller, C. and Garabédian, M. and George, A. and Goldberg, H. and Menashi, S.
Rickets and child abuse: The case of a two year old girl from the 4th century in Lisieux (Normandy)	2002	International Journal of Osteoarchaeology	Blondiaux, G. and Blondiaux, J. and Secousse, F. and Cotten, A. and Danze, P.-M. and Flipo, R.-M.
FGF-23 in fibrous dysplasia of bone and its relationship to renal phosphate wasting	2003	J. Clin. Invest.	Riminucci, M. and Collins, M.T. and Fedarko, N.S. and Cherman, N. and Corsi, A. and White, K.E. and Waguespack, S. and Gupta, A. and Hannon, T. and Econs, M.J. and Bianco, P. and Robey, P.G.
Diagnosis and management of unusual dental abscesses in children.	2003	Australian dental journal	Seow WK
FGF-23 is elevated by chronic hyperphosphatemia	2004	J. Clin. Endocrinol. Metab.	Gupta, A. and Winer, K. and Econs, M.J. and Marx, S.J. and Collins, M.T.
Expression of dentin matrix protein 1 in tumors causing oncogenic osteomalacia	2004	Mod. Pathol.	Toyosawa, S. and Tomita, Y. and Kishino, M. and Hashimoto, J. and Ueda, T. and Tsujimura, T. and Aozasa, K. and Ijuhin, N. and Komori, T.
Cartilage abnormalities are associated with abnormal Phex expression and with altered matrix protein and MMP-9 localization in Hyp mice	2004	Bone	Miao, D. and Bai, X. and Panda, D.K. and Karaplis, A.C. and Goltzman, D. and McKee, M.D.
Inorganic phosphate homeostasis and the role of dietary phosphorus.	2004	Journal of cellular and molecular medicine	Takeda E and Yamamoto H and Nashiki K and Sato T and Arai H and Taketani Y
The wrickkened pathways of FGF23, MEPE and PHEX.	2004	Critical reviews in oral biology and medicine : an official publication of the	Rowe PS

		American Association of Oral Biologists	
Post-translational modifications of sibling proteins and their roles in osteogenesis and dentinogenesis.	2004	Critical reviews in oral biology and medicine : an official publication of the American Association of Oral Biologists	Qin C and Baba O and Butler WT
Homozygous ablation of fibroblast growth factor-23 results in hyperphosphatemia and impaired skeletogenesis, and reverses hypophosphatemia in Phex-deficient mice.	2004	Matrix biology : journal of the International Society for Matrix Biology	Sitara D and Razzaque MS and Hesse M and Yoganathan S and Taguchi T and Erben RG and Jüppner H and Lanske B
A synthetic peptide fragment of human MEPE stimulates new bone formation in vitro and in vivo.	2004	Journal of bone and mineral research : the official journal of the American Society for Bone and Mineral Research	Hayashibara T and Hiraga T and Yi B and Nomizu M and Kumagai Y and Nishimura R and Yoneda T
Differential regulation of PHEX expression in bone and parathyroid gland by chronic renal insufficiency and 1,25-dihydroxyvitamin D3	2004	American Journal of Physiology - Renal Physiology	Brewer, A.J. and Canaff, L. and Hendy, G.N. and Tenenhouse, H.S.
Dent's disease: Identification of a novel mutation in the renal chloride channel CLCN5	2004	Clinical Nephrology	Brakemeier, S. and Si, H. and Gollasch, M. and Höffler, D. and Buhl, M. and Köhler, R. and Hoyer, J. and Eichler, I.
Effect of gene dose and parental origin on bone histomorphometry in X-linked Hyp mice	2004	Bone	Qiu, Z.Q. and Travers, R. and Rauch, F. and Glorieux, F.H. and Scriver, C.R. and Tenenhouse, H.S.
Identification of a novel splice site mutation of CLCN5 gene and characterization of a new alternative 5′ UTR end of ClC-5 mRNA in human renal tissue and leukocytes	2004	Journal of Human Genetics	Forino, M. and Graziotto, R. and Tosetto, E. and Gambaro, G. and D'Angelo, A. and Anglani, F.
[Hereditary hypophosphatemia in adults].	2005	Presse medicale (Paris, France : 1983)	Vélayoudom-Céphise FL and Vantyghem MC and Wémeau JL
[Autosomal dominant hypophosphatemic rickets/osteomalacia].	2005	Nihon rinsho. Japanese journal of clinical medicine	Tanaka H
Comparison of prescriptions by pediatricians and general practitioners: A population-based study in Franche-Comté from the database of Regional Health Insurance Fund	2005	Archives de Pediatrie	Bocquet, A. and Chalumeau, M. and Bollotte, D. and Escano, G. and Langue, J. and Virey, B.
Phenotype and genotype of Dent's disease in three Korean boys	2005	Pediatric Nephrology	Cheong, H.I. and Lee, J.W. and Zheng, S.H. and Lee, J.H. and Kang, J.H. and Kang, H.G. and Ha, I.S. and Lee, S.J. and Choi, Y.
Hyper-expression of osteocalcin mRNA in odontoblasts of Hyp mice	2005	Journal of Dental Research	Onishi, T. and Ogawa, T. and Hayashibara, T. and Hoshino, T. and Okawa, R. and Ooshima, T.
Role of matrix extracellular phosphoglycoprotein in the pathogenesis of x-linked hypophosphatemia	2005	Journal of the American Society of Nephrology	Liu, S. and Brown, T.A. and Zhou, J. and Xiao, Z.-S. and Awad, H. and Guilak, F. and Quarles, L.D.

Incidental finding of vitamin-D deficient rickets in an otherwise healthy infant - A reappraisal of current vitamin-D supplementation guidelines	2005	Journal of the National Medical Association	Alouf, B. and Grigalonis, M.
Role of prostaglandins in the pathogenesis of X-linked hypophosphatemia.	2006	Pediatric nephrology (Berlin, Germany)	Baum M and Syal A and Quigley R and Seikaly M
Molecular bases of diseases characterized by hypophosphatemia and phosphaturia: new understanding.	2006	Clinical pediatric endocrinology : case reports and clinical investigations : official journal of the Japanese Society for Pediatric Endocrinology	Ozono K and Michigami T and Namba N and Nakajima S and Yamamoto T
X-linked hypophosphatemia: dental and histologic findings.	2006	Journal (Canadian Dental Association)	Batra P and Tejani Z and Mars M
[X-linked hypophosphatemic vitamin D resistant rickets: Pathogenesis, pathophysiology, and therapy].	2006	Nihon rinsho. Japanese journal of clinical medicine	Tanaka H
Osteocytes as multifunctional cells	2006	Journal of Musculoskeletal Neuronal Interactions	Bonewald, L.F.
Hypophosphatemic rickets: Results of a long-term follow-up	2006	Pediatric Nephrology	Vaisbich, M.H. and Koch, V.H.
Dentinal defects in Hyp mice not caused by hypophosphatemia alone	2006	Archives of Oral Biology	Ogawa, T. and Onishi, T. and Hayashibara, T. and Sakashita, S. and Okawa, R. and Ooshima, T.
Loss of DMP1 causes rickets and osteomalacia and identifies a role for osteocytes in mineral metabolism	2006	Nature Genetics	Feng, J.Q. and Ward, L.M. and Liu, S. and Lu, Y. and Xie, Y. and Yuan, B. and Yu, X. and Rauch, F. and Davis, S.I. and Zhang, S. and Rios, H. and Drezner, M.K. and Quarles, L.D. and Bonewald, L.F. and White, K.E.
Dentin alteration of deciduous teeth in human hypophosphatemic rickets	2006	Calcified Tissue International	Boukpessi, T. and Septier, D. and Bagga, S. and Garabedian, M. and Goldberg, M. and Chaussain-Miller, C.
Early lethality in Hyp mice with targeted deletion of pth gene	2007	Endocrinology	Bai, X. and Miao, D. and Goltzman, D. and Karaplis, A.C.
Fibrous dysplasia, phosphate wasting and fibroblast growth factor 23	2007	Pediatr. Endocrinol. Rev.	Imel, E.A. and Econs, M.J.
Clinical vignette: Cinacalcet in the management of tumor-induced osteomalacia	2007	J. Bone Miner. Res.	Geller, J.L. and Khosravi, A. and Kelly, M.H. and Riminucci, M. and Adams, J.S. and Collins, M.T.
Determination of the elimination half-life of fibroblast growth factor-23	2007	J. Clin. Endocrinol. Metab.	Khosravi, A. and Cutler, C.M. and Kelly, M.H. and Chang, R. and Royal, R.E. and Sherry, R.M. and Wodajo, F.M. and Fedarko, N.S. and Collins, M.T.
Novel regulators of phosphate homeostasis and bone metabolism.	2007	Therapeutic apheresis and dialysis : official peer-reviewed journal of the International Society for Apheresis, the Japanese Society for Apheresis,	Jüppner H

		the Japanese Society for Dialysis Therapy	
Hereditary hypophosphatemias: new genes in the bone-kidney axis.	2007	Nephrology (Carlton, Vic.)	Negri AL
Dentin matrix protein 1 (DMP1): new and important roles for biomineralization and phosphate homeostasis.	2007	Journal of dental research	Qin C and D'Souza R and Feng JQ
Learn to recognize rickets.	2007	The New York state dental journal	Archard HO
Update: Update in osteoporosis and metabolic bone disorders	2007	Journal of Clinical Endocrinology and Metabolism	Shoback, D.
Phosphorylated acidic serine-aspartate-rich MEPE-associated motif peptide from matrix extracellular phosphoglycoprotein inhibits phosphate regulating gene with homologies to endopeptidases on the X-chromosome enzyme activity	2007	Journal of Endocrinology	Liu, S. and Rowe, P.S.N. and Vierthaler, L. and Zhou, J. and Quarles, L.D.
Rickets	2007	Paediatrics and Child Health	Dimitri, P. and Bishop, N.
Oral findings of hypophosphatemic vitamin D-resistant rickets: Report of two cases	2007	Chinese Medical Journal	Su, J.-M. and Li, Y. and Ye, X.-W. and Wu, Z.-F.
Dentin structure in familial hypophosphatemic rickets: Benefits of vitamin D and phosphate treatment	2007	Oral Diseases	Chaussain-Miller, C. and Sinding, C. and Septier, D. and Wolikow, M. and Goldberg, M. and Garabedian, M.
Phex mutation causes the reduction of Npt2b mRNA in teeth	2007	Journal of Dental Research	Onishi, T. and Okawa, R. and Ogawa, T. and Shintani, S. and Ooshima, T.
Clinical usefulness of measurement of fibroblast growth factor 23 (FGF23) in hypophosphatemic patients. Proposal of diagnostic criteria using FGF23 measurement	2008	Bone	Endo, I. and Fukumoto, S. and Ozono, K. and Namba, N. and Tanaka, H. and Inoue, D. and Minagawa, M. and Sugimoto, T. and Yamauchi, M. and Michigami, T. and Matsumoto, T.
Detection of the primary tumor site in tumor-induced osteomalacia by indium-111 octreotide scintigraphy: A case report	2008	Clin. Neurol.	Takahashi, M. and Toru, S. and Ota, K. and Izumiyama, H. and Yokota, T. and Mizusawa, H.
Inherited hypophosphatemic disorders in children and the evolving mechanisms of phosphate regulation.	2008	Reviews in endocrine & metabolic disorders	Bastepe M and Jüppner H
[Inherited monogenic kidney stone diseases: recent diagnostic and therapeutic advances].	2008	Nephrologie & therapeutique	Jungers P and Joly D and Blanchard A and Courbebaisse M and Knebelmann B and Daudon M
Renal phenotype in Lowe syndrome: A selective proximal tubular dysfunction	2008	Clinical Journal of the American Society of Nephrology	Bockenhauer, D. and Bokenkamp, A. and van't Hoff, W. and Levtchenko, E. and Kist-van Holthe, J.E. and Tasic, V. and Ludwig, M.
Vitamin-D-resistant rickets: Report of a case	2008	New Zealand Dental Journal	Foster Page, L.A.

Degradation of MEPE, DMP1, and release of SIBLING ASARM-peptides (minhibins): ASARM-Peptide(s) are directly responsible for defective mineralization in HYP	2008	Endocrinology	Martin, A. and David, V. and Laurence, J.S. and Schwarz, P.M. and Lafer, E.M. and Hedge, A.-M. and Rowe, P.S.N.
Periodontal breakdown in the Dmp1 null mouse model of hypophosphatemic rickets	2008	Journal of Dental Research	Ye, L. and Zhang, S. and Ke, H. and Bonewald, L.F. and Feng, J.Q.
Phex mutation causes overexpression of FGF23 in teeth	2008	Archives of Oral Biology	Onishi, T. and Umemura, S. and Shintani, S. and Ooshima, T.
MEPE-ASARM peptides control extracellular matrix mineralization by binding to hydroxyapatite: An inhibition regulated by PHEX cleavage of ASARM	2008	Journal of Bone and Mineral Research	Addison, W.N. and Nakano, Y. and Loisel, T. and Crine, P. and McKee, M.D.
Hereditary dentine disorders: Dentinogenesis imperfecta and dentine dysplasia	2008	Orphanet Journal of Rare Diseases	Barron, M.J. and McDonnell, S.T. and MacKie, I. and Dixon, M.J.
Generation of a conditional null allele for Dmp1 in mouse	2008	Genesis	Feng, J.Q. and Scott, G. and Guo, D. and Jiang, B. and Harris, M. and Ward, T. and Ray, M. and Bonewald, L.F. and Harris, S.E. and Mishina, Y.
Pathogenic role of Fgf23 in Dmp1-null mice	2008	American Journal of Physiology - Endocrinology and Metabolism	Liu, S. and Zhou, J. and Tang, W. and Menard, R. and Feng, J.Q. and Quarles, L.D.
PHEX analysis in 118 pedigrees reveals new genetic clues in hypophosphatemic rickets	2009	Hum. Genet.	Gaucher, C. and Walrant-Debray, O. and Nguyen, T.-M. and Esterle, L. and Garabédian, M. and Jehan, F.
Recombinant MEPE can increase hydroxyapatite in vitro	2009	Afr. J. Biotechnol.	Ai, L. and Wang, H. and Xiao, M. and Ni, L. and Wang, H.
Npt2a and Npt2c in mice play distinct and synergistic roles in inorganic phosphate metabolism and skeletal development	2009	Am. J. Physiol. Renal Physiol.	Segawa, H. and Onitsuka, A. and Furutani, J. and Kaneko, I. and Aranami, F. and Matsumoto, N. and Tomoe, Y. and Kuwahata, M. and Ito, M. and Matsumoto, M. and Li, M. and Amizuka, N. and Miyamoto, K.-I.
Molecular pathology of the fibroblast growth factor family.	2009	Human mutation	Krejci P and Prochazkova J and Bryja V and Kozubik A and Wilcox WR
Dentin noncollagenous matrix proteins in familial hypophosphatemic rickets.	2009	Cells, tissues, organs	Gaucher C and Boukpessi T and Septier D and Jehan F and Rowe PS and Garabédian M and Goldberg M and Chaussain-Miller C
Studies of the DMP1 57-kDa functional domain both in vivo and in vitro.	2009	Cells, tissues, organs	Lu Y and Qin C and Xie Y and Bonewald LF and Feng JQ
Incidence and prevalence of nutritional and hereditary rickets in southern Denmark	2009	European Journal of Endocrinology	Beck-Nielsen, S.S. and Brock-Jacobsen, B. and

			Gram, J. and Brixen, K. and Jensen, T.K.
Vitamin A responsive night blindness in Dent's disease	2009	Pediatric Nephrology	Sethi, S.K. and Ludwig, M. and Kabra, M. and Hari, P. and Bagga, A.
Familial hypophosphatemic rickets caused by a large deletion in PHEX gene	2009	European Journal of Endocrinology	Saito, T. and Nishii, Y. and Yasuda, T. and Ito, N. and Suzuki, H. and Igarashi, T. and Fukumoto, S. and Fujita, T.
Bone health as a primary target in the pediatric age	2009	European Review for Medical and Pharmacological Sciences	Caradonna, P. and Rigante, D.
Discordance for X-Linked hypophosphataemic rickets in identical twin girls	2009	Hormone Research	Owen, C.J. and Habeb, A. and Pearce, S.H.S. and Wright, M. and Ichikawa, S. and Sorenson, A.H. and Econs, M.J. and Cheetham, T.D.
Familial hypophosphatemic vitamin D-resistant rickets-prevention of spontaneous dental abscesses on primary teeth: A case report	2009	Oral Surgery, Oral Medicine, Oral Pathology, Oral Radiology and Endodontology	Douyere, D. and Joseph, C. and Gaucher, C. and Chaussain, C. and Courson, F.
The PHEX transgene corrects mineralization defects in 9-month-old hypophosphatemic mice	2009	Calcified Tissue International	Boskey, A. and Frank, A. and Fujimoto, Y. and Spevak, L. and Verdelis, K. and Ellis, B. and Troiano, N. and Philbrick, W. and Carpenter, T.
Molecular analysis of DMP1 mutants causing autosomal recessive hypophosphatemic rickets	2009	Bone	Farrow, E.G. and Davis, S.I. and Ward, L.M. and Summers, L.J. and Bubbear, J.S. and Keen, R. and Stamp, T.C.B. and Baker, L.R.I. and Bonewald, L.F. and White, K.E.
Novel regulators of Fgf23 expression and mineralization in Hyp bone	2009	Molecular Endocrinology	Liu, S. and Tang, W. and Fang, J. and Ren, J. and Li, H. and Xiao, Z. and Quarles, L.D.
Craniofacial morphology in patients with hypophosphataemic vitamin-D-resistant rickets: A cephalometric study	2009	Journal of Oral Rehabilitation	Al-Jundi, S.H. and Dabous, I.M. and Al-Jamal, G.A.
Case histories	2009	Endocrine Development	Howard, S. and Lyder, G. and Allgrove, J. and Shaw, N.
Emerging Topics in Pediatric Bone and Mineral Disorders 2008	2009	Seminars in Nephrology	McKay, C.P. and Portale, A.
Aberrant cementum phenotype associated with the hypophosphatemic Hyp mouse	2009	Journal of Periodontology	Fong, H. and Chu, E.Y. and Tompkins, K.A. and Foster, B.L. and Sitara, D. and Lanske, B. and Somerman, M.J.
Dental Development in Patients with Hypophosphatemia Rickets.	2010	International journal of clinical pediatric dentistry	Sh AJ and Am H
Regulation of phosphate transport by fibroblast growth factor 23 (FGF23): implications for disorders of phosphate metabolism.	2010	Pediatric nephrology (Berlin, Germany)	Gattineni J and Baum M

[Genetic basis for skeletal disease. Dental management of patients with bone diseases].	2010	Clinical calcium	Shintani S and Ooshima T
Phosphorylation-dependent inhibition of mineralization by osteopontin ASARM peptides is regulated by PHEX cleavage	2010	Journal of Bone and Mineral Research	Addison, W.N. and Masica, D.L. and Gray, J.J. and McKee, M.D.
A novel nonsense mutation in the DMP1 gene in a Japanese family with autosomal recessive hypophosphatemic rickets	2010	Journal of Bone and Mineral Metabolism	Koshida, R. and Yamaguchi, H. and Yamasaki, K. and Tsuchimochi, W. and Yonekawa, T. and Nakazato, M.
A 7-year-old girl with ongoing weight loss	2010	Pediatric Annals	Zipkin, R.
DMP1 C-terminal mutant mice recapture the human ARHR tooth phenotype	2010	Journal of Bone and Mineral Research	Jiang, B. and Cao, Z. and Lu, Y. and Janik, C. and Lauziere, S. and Xie, Y. and Poliard, A. and Qin, C. and Ward, L.M. and Feng, J.Q.
Familial hypophosphatemic rickets	2010	Journal of Indian Society of Pedodontics and Preventive Dentistry	Sattur, A. and Naikmasur, V.G. and Shrivastava, R. and Babshet, M.
Multiple nonvital teeth. Vitamin D-resistant rickets.	2010	General dentistry	Damm, D.D.
Dental arch dimensions in children with hypophosphataemic Vitamin D resistant rickets.	2010	European archives of paediatric dentistry : official journal of the European Academy of Paediatric Dentistry	Al-Jundi, S.H. and Al-Naimy, Y.F. and Alsweedan, S.
Refractory rickets in the tropics	2010	Journal of Pediatric Endocrinology and Metabolism	Sahay, M. and Sahay, R.K.
Abnormal presence of the matrix extracellular phosphoglycoprotein-derived acidic serine- and aspartate-rich motif peptide in human hypophosphatemic dentin	2010	American Journal of Pathology	Boukpessi, T. and Gaucher, C. and Léger, T. and Salmon, B. and Le Faouder, J. and Willig, C. and Rowe, P.S. and Garabédian, M. and Meilhac, O. and Chaussain, C.
Expression and distribution of SIBLING proteins in the predentin/dentin and mandible of hyp mice	2010	Oral Diseases	Zhang, B. and Sun, Y. and Chen, L. and Guan, C. and Guo, L. and Qin, C.
Long-term clinical outcome and carrier phenotype in autosomal recessive hypophosphatemia caused by a novel DMP1 mutation	2010	Journal of Bone and Mineral Research	Mäkitie, O. and Pereira, R.C. and Kaitila, I. and Turan, S. and Bastepe, M. and Laine, T. and Kröger, H. and Cole, W.G. and Jüppner, H.
Loss-of-Function ENPP1 Mutations Cause Both Generalized Arterial Calcification of Infancy and Autosomal-Recessive Hypophosphatemic Rickets	2010	American Journal of Human Genetics	Lorenz-Depiereux, B. and Schnabel, D. and Tiosano, D. and Häusler, G. and Strom, T.M.
Ablation of systemic phosphate-regulating gene fibroblast growth factor 23 (Fgf23) compromises the dentoalveolar complex	2010	Anatomical Record	Chu, E.Y. and Fong, H. and Blethen, F.A. and Tompkins, K.A. and Foster, B.L. and Yeh, K.D. and Nagatomo, K.J. and Matsa-Dunn, D. and Sitara, D. and Lanske, B. and Rutherford, R.B. and Somerman, M.J.

The importance of whole body imaging in tumor-induced osteomalacia	2011	J. Clin. Endocrinol. Metab.	Chong, W.H. and Yavuz, S. and Patel, S.M. and Chen, C.C. and Collins, M.T.
Craniofacial morphology in patients with hypophosphatemic rickets: A cephalometric study focusing on differences between bone of cartilaginous and intramembranous origin	2011	Am. J. Med. Genet. Part A	Gjørup, H. and Kjær, I. and Sonnesen, L. and Haubek, D. and Beck-Nielsen, S.S. and Hintze, H. and Poulsen, S.
Case 33-2011: A 56-year-old man with hypophosphatemia	2011	New Engl. J. Med.	Bergwitz, C. and Collins, M.T. and Kamath, R.S. and Rosenberg, A.E.
Papilledema in the setting of X-linked hypophosphatemic rickets with craniosynostosis	2011	Case Rep. Ophthalmol.	Dagi Glass, L.R. and Forcht Dagi, T. and Dagi, L.R.
Selective venous catheterization for the localization of phosphaturic mesenchymal tumors	2011	J. Bone Miner. Res.	Andreopoulou, P. and Dumitrescu, C.E. and Kelly, M.H. and Brillante, B.A. and Cutler Peck, C.M. and Wodajo, F.M. and Chang, R. and Collins, M.T.
FGF23 in skeletal modeling and remodeling.	2011	Current osteoporosis reports	Lu Y and Feng JQ
Cellular ATP synthesis mediated by type III sodium-dependent phosphate transporter Pit-1 is critical to chondrogenesis.	2011	The Journal of biological chemistry	Sugita A and Kawai S and Hayashibara T and Amano A and Ooshima T and Michigami T and Yoshikawa H and Yoneda T
Asarm peptides: PHEX-dependent and -independent regulation of serum phosphate	2011	American Journal of Physiology - Renal Physiology	David, V. and Martin, A. and Hedge, A.-M. and Drezner, M.K. and Rowe, P.S.N.
Unique roles of phosphorus in endochondral bone formation and osteocyte maturation	2011	Journal of Bone and Mineral Research	Zhang, R. and Lu, Y. and Ye, L. and Yuan, B. and Yu, S. and Qin, C. and Xie, Y. and Gao, T. and Drezner, M.K. and Bonewald, L.F. and Feng, J.Q.
Kbus/Idr, a mutant mouse strain with skeletal abnormalities and hypophosphatemia: Identification as an allele of 'Hyp'	2011	Journal of Biomedical Science	Moriyama, K. and Hanai, A. and Mekada, K. and Yoshiki, A. and Ogiwara, K. and Kimura, A. and Takahashi, T.
Effect of growth hormone replacement therapy in a boy with Dent's disease: A case report	2011	Journal of Medical Case Reports	Samardzic, M. and Pavicevic, S. and Ludwig, M. and Bogdanovic, R.
An atypical degenerative osteoarthropathy in Hyp mice is characterized by a loss in the mineralized zone of articular cartilage	2011	Calcified Tissue International	Liang, G. and Vanhouten, J. and MacIca, C.M.
Mutational analysis of PHEX, FGF23 and DMP1 in a cohort of patients with hypophosphatemic rickets	2011	Clinical Endocrinology	Ruppe, M.D. and Brosnan, P.G. and Au, K.S. and Tran, P.X. and Dominguez, B.W. and Northrup, H.
PHEX neutralizing agent inhibits dentin formation in mouse tooth germ	2011	Tissue and Cell	Lv, H. and Fu, S. and Wu, G. and Yan, F.

A novel nonsense mutation in the DMP1 gene identified by a Genome-Wide association study is responsible for Inherited rickets in Corriedale sheep	2011	PLoS ONE	Zhao, X. and Dittmer, K.E. and Blair, H.T. and Thompson, K.G. and Rothschild, M.F. and Garrick, D.J.
Growth hormone treatment in a child with X-linked hypophosphataemic rickets	2011	Medical Journal Armed Forces India	Prasad, A.N. and Holla, R.G.
A patient with hypophosphatemic rickets and ossification of posterior longitudinal ligament caused by a novel homozygous mutation in ENPP1 gene	2011	Bone	Saito, T. and Shimizu, Y. and Hori, M. and Taguchi, M. and Igarashi, T. and Fukumoto, S. and Fujitab, T.
The biological function of DMP-1 in osteocyte maturation is mediated by its 57-kDa c-terminal fragment	2011	Journal of Bone and Mineral Research	Lu, Y. and Yuan, B. and Qin, C. and Cao, Z. and Xie, Y. and Dallas, S.L. and McKee, M.D. and Drezner, M.K. and Bonewald, L.F. and Feng, J.Q.
Bone proteins PHEX and DMP1 regulate fibroblastic growth factor Fgf23 expression in osteocytes through a common pathway involving FGF receptor (FGFR) signaling	2011	FASEB Journal	Martin, A. and Liu, S. and David, V. and Li, H. and Karydis, A. and Feng, J.Q. and Quarles, L.D.
Hypophosphatemic rickets is associated with disruption of mineral orientation at the nanoscale in the flat scapula bones of rachitic mice with development	2012	Bone	Karunaratne, A. and Davis, G.R. and Hiller, J. and Esapa, C.T. and Terrill, N.J. and Brown, S.D.M. and Cox, R.D. and Thakker, R.V. and Gupta, H.S.
Significant deterioration in nanomechanical quality occurs through incomplete extrafibrillar mineralization in rachitic bone: Evidence from in-situ synchrotron X-ray scattering and backscattered electron imaging	2012	J. Bone Miner. Res.	Karunaratne, A. and Esapa, C.R. and Hiller, J. and Boyde, A. and Head, R. and Bassett, J.H.D. and Terrill, N.J. and Williams, G.R. and Brown, M.A. and Croucher, P.I. and Brown, S.D.M. and Cox, R.D. and Barber, A.H. and Thakker, R.V. and Gupta, H.S.
Hypophosphatemic rickets.	2012	Current opinion in endocrinology, diabetes, and obesity	Baroncelli GI and Toschi B and Bertelloni S
[Osteocyte and DMP1].	2012	Clinical calcium	Toyosawa S and Oya K and Sato S and Ishida K
The chicken or the egg: PHEX, FGF23 and SIBLINGs unscrambled.	2012	Cell biochemistry and function	Rowe PS
Tooth dentin defects reflect genetic disorders affecting bone mineralization.	2012	Bone	Opsahl Vital S and Gaucher C and Bardet C and Rowe PS and George A and Linglart A and Chaussain C
Regulation of bone-renal mineral and energy metabolism: the PHEX, FGF23, DMP1, MEPE ASARM pathway.	2012	Critical reviews in eukaryotic gene expression	Rowe PS
Mechanism of FGF23 processing in fibrous dysplasia.	2012	Journal of bone and mineral research : the official journal of the American Society for Bone and Mineral Research	Bhattacharyya N and Wiench M and Dumitrescu C and Connolly BM and Bugge TH and Patel HV and Gafni RI and Cherman

			N and Cho M and Hager GL and Collins MT
Hypophosphatemic rickets: A case of recurrent pathological fractures.	2012	Indian journal of endocrinology and metabolism	Baidya A and Chowdhury S and Mukhopadhyay S and Ghosh S
McGill researcher identifies protein involved in rare bone and tooth disease.	2012	Journal (Canadian Dental Association)	
Protective roles of DMP1 in high phosphate homeostasis	2012	PLoS ONE	Rangiani, A. and Cao, Z. and Sun, Y. and Lu, Y. and Gao, T. and Yuan, B. and Rodgers, A. and Qin, C. and Kuro-o, M. and Feng, J.Q.
Nephrology Quiz and questionnaire: Electrolytes	2012	Clinical Journal of the American Society of Nephrology	Palmer, B.F. and Glassock, R.J. and Bleyer, A.J.
Amelogenesis Imperfecta and Other Biomineralization Defects in Fam20a and Fam20c Null Mice	2012	Veterinary Pathology	Vogel, P. and Hansen, G.M. and Read, R.W. and Vance, R.B. and Thiel, M. and Liu, J. and Wronski, T.J. and Smith, D.D. and Jeter-Jones, S. and Brommage, R.
Rickets in Denmark: Prevalence of nutritional and hereditary rickets among children living in Denmark and characteristics of patients with hypophosphatemic rickets	2012	Danish Medical Journal	Beck-Nielsen, S.S.
Mineralizing enthesopathy is a common feature of renal phosphate-wasting disorders attributed to FGF23 and is exacerbated by standard therapy in hyp mice	2012	Endocrinology	Karaplis, A.C. and Bai, X. and Falet, J.-P. and Macica, C.M.
PTHrP(1-34)-mediated repression of the PHEX gene in osteoblastic cells involves the transcriptional repressor E4BP4	2012	Journal of Cellular Physiology	Pellicelli, M. and Taheri, M. and St-Louis, M. and Bériault, V. and Desgroseillers, L. and Boileau, G. and Moreau, A.
Overexpression of the DMP1 C-terminal fragment stimulates FGF23 and exacerbates the hypophosphatemic rickets phenotype in Hyp mice	2012	Molecular Endocrinology	Martin, A. and David, V. and Li, H. and Dai, B. and Feng, J.Q. and Quarles, L.D.
Endodontic management in a patient with vitamin D-resistant rickets	2012	Journal of Endodontics	Beltes, C. and Zachou, E.
FAM20C plays an essential role in the formation of murine teeth	2012	Journal of Biological Chemistry	Wang, X. and Wang, S. and Lu, Y. and Gibson, M.P. and Liu, Y. and Yuan, B. and Feng, J.Q. and Qin, C.
[Spontaneous dental abscesses in familial hypophosphatemic rickets].	2012	Nederlands tijdschrift voor tandheelkunde	van Riet, T.C. and Frank, M.H. and Hoeben, K.A. and de Lange, J.
Inactivation of a novel FGF23 regulator, FAM20C, leads to hypophosphatemic rickets in mice	2012	PLoS Genetics	Wang, X. and Wang, S. and Li, C. and Gao, T. and Liu, Y. and Rangiani, A. and Sun, Y. and Hao, J. and George, A. and Lu, Y. and Groppe, J. and Yuan, B. and Feng, J.Q. and Qin, C.

FGF23 analysis of a Chinese family with autosomal dominant hypophosphatemic rickets	2012	Journal of Bone and Mineral Metabolism	Sun, Y. and Wang, O. and Xia, W. and Jiang, Y. and Li, M. and Xing, X. and Hu, Y. and Liu, H. and Meng, X. and Zhou, X.
Evidence for FGF23 involvement in a bone-kidney axis regulating bone mineralization and systemic phosphate and vitamin D homeostasis	2012	Advances in Experimental Medicine and Biology	Martin, A. and Quarles, L.D.
FGF23 and syndromes of abnormal renal phosphate handling	2012	Advances in Experimental Medicine and Biology	Bergwitz, C. and Jüppner, H.
Rickets-like genetic diseases	2013	Chin. J. Contemp. Pediatr.	Ma, H.-W.
Hypercalciuria	2013	Hipercalciuria	González-Lamuño, D.
Crystal structures of hereditary vitamin D-resistant rickets-associated vitamin D receptor mutants R270l and W282R bound to 1,25-dihydroxyvitamin D 3 and synthetic ligands	2013	J. Med. Chem.	Nakabayashi, M. and Tsukahara, Y. and Iwasaki-Miyamoto, Y. and Mihori-Shimazaki, M. and Yamada, S. and Inaba, S. and Oda, M. and Shimizu, M. and Makishima, M. and Tokiwa, H. and Ikura, T. and Ito, N.
Characterization of the testicular, epididymal and endocrine phenotypes in the Leuven Vdr-deficient mouse model: Targeting estrogen signalling	2013	Mol. Cell. Endocrinol.	Blomberg Jensen, M. and Lieben, L. and Nielsen, J.E. and Willems, A. and Jørgensen, A. and Juul, A. and Toppari, J. and Carmeliet, G. and Rajpert-De Meyts, E.
[Hypophosphatemic rickets. Hereditary disorder of metabolism and dentin dysplasia].	2013	Hypophosphatämische Rachitis. Hereditäre Stoffwechselstörung und Dentinfehlbildung.	Van Waes, H. and Luder, H.U.
Exome sequencing reveals FAM20c mutations associated with fibroblast growth factor 23-related hypophosphatemia, dental anomalies, and ectopic calcification	2013	J. Bone Miner. Res.	Rafaelsen, S.H. and Ræder, H. and Fagerheim, A.K. and Knappskog, P. and Carpenter, T.O. and Johansson, S. and Bjerknes, R.
Tumor localization and biochemical response to cure in tumor-induced osteomalacia	2013	J. Bone Miner. Res.	Chong, W.H. and Andreopoulou, P. and Chen, C.C. and Reynolds, J. and Guthrie, L. and Kelly, M. and Gafni, R.I. and Bhattacharyya, N. and Boyce, A.M. and El-Maouche, D. and Crespo, D.O. and Sherry, R. and Chang, R. and Wodajo, F.M. and Kletter, G.B. and Dwyer, A. and Collins, M.T.
When to suspect a genetic disorder in a patient with renal stones, and why	2013	Nephrol. Dial. Transplant.	Ferraro, P.M. and D'Addessi, A. and Gambaro, G.
Evaluation of targeted next-generation sequencing-based preimplantation genetic diagnosis of monogenic disease	2013	Fertil. Steril.	Treff, N.R. and Fedick, A. and Tao, X. and Devkota, B. and Taylor, D. and Scott Jr., R.T.

Osteopetrosis, osteopetrorickets and hypophosphatemic rickets differentially affect dentin and enamel mineralization	2013	Bone	Koehne, T. and Marshall, R.P. and Jeschke, A. and Kahl-Nieke, B. and Schinke, T. and Amling, M.
Proteolytic processing of osteopontin by PHEX and accumulation of osteopontin fragments in Hyp mouse bone, the murine model of X-linked hypophosphatemia	2013	J. Bone Miner. Res.	Barros, N.M.T. and Hoac, B. and Neves, R.L. and Addison, W.N. and Assis, D.M. and Murshed, M. and Carmona, A.K. and McKee, M.D.
Insights From Genetic Disorders of Phosphate Homeostasis	2013	Semin. Nephrol.	Christov, M. and Jüppner, H.
[Updates on rickets and osteomalacia. dental diseases in rickets].	2013	Clinical calcium	Kubota T
Osteo-renal cross-talk and phosphate metabolism by the FGF23-Klotho system.	2013	Contributions to nephrology	Ohnishi M and Razzaque MS
Hypophosphatemic rickets and its dental significance.	2013	Iranian journal of pediatrics	Ziaee V and Rabbani A
Osteocyte regulation of phosphate homeostasis and bone mineralization underlies the pathophysiology of the heritable disorders of rickets and osteomalacia.	2013	Bone	Feng JQ and Clinkenbeard EL and Yuan B and White KE and Drezner MK
Hypophosphatemic rickets and its dental significance.	2013	Iranian journal of pediatrics	Ramazani N
Oral manifestations of vitamin D resistant rickets in orthopantomogram.	2013	BMJ case reports	Rathore R and Nalawade TM and Pateel D and Mallikarjuna R
Morphological characteristics of frontal sinus and nasal bone focusing on bone resorption and apposition in hypophosphatemic rickets	2013	Orthodontics and Craniofacial Research	Gjørup, H. and Kjaer, I. and Sonnesen, L. and Beck-Nielsen, S.S. and Haubek, D.
Dentin matrix protein 1 and phosphate homeostasis are critical for postnatal pulp, dentin and enamel formation	2013	International Journal of Oral Science	Rangiani, A. and Cao, Z.-G. and Liu, Y. and Voisey Rodgers, A. and Jiang, Y. and Qin, C.-L. and Feng, J.-Q.
MEPE-Derived ASARM Peptide Inhibits Odontogenic Differentiation of Dental Pulp Stem Cells and Impairs Mineralization in Tooth Models of X-Linked Hypophosphatemia	2013	PLoS ONE	Salmon, B. and Bardet, C. and Khaddam, M. and Naji, J. and Coyac, B.R. and Baroukh, B. and Letourneur, F. and Lesieur, J. and Decup, F. and Le Denmat, D. and Nicoletti, A. and Poliard, A. and Rowe, P.S. and Huet, E. and Vital, S.O. and Linglart, A. and McKee, M.D. and Chaussain, C.
Clinical approach in familial hypophosphatemic rickets: Report of three generations	2013	Special Care in Dentistry	Soares, E.C.S. and Costa, F.W.G. and Ribeiro, T.R. and Alves, A.P.N.N. and Fonteles, C.S.R.
Type II vitamin D-dependent rickets with diabetic ketoacidosis	2013	Journal of Pediatric Endocrinology and Metabolism	Sarkar, S. and Mondal, R. and Banerjee, I. and Sabui, T.
Dosage effect of a phex mutation in a murine model of X-linked hypophosphatemia	2013	Calcified Tissue International	Ichikawa, S. and Gray, A.K. and Bikorimana, E. and Econs, M.J.

Hexa-D-arginine treatment increases 7B2•PC2 activity in hyp-mouse osteoblasts and rescues the HYP phenotype	2013	Journal of Bone and Mineral Research	Yuan, B. and Feng, J.Q. and Bowman, S. and Liu, Y. and Blank, R.D. and Lindberg, I. and Drezner, M.K.
Dental manifestations of patient with Vitamin D-resistant rickets	2013	Journal of Applied Oral Science	Souza, A.P. and Kobayashi, T.Y. and Lourenço Neto, N. and Silva, S.M.B. and Machado, M.A.A.M. and Oliveira, T.M.
Pharmacological inhibition of fibroblast growth factor (FGF) receptor signaling ameliorates FGF23-mediated hypophosphatemic rickets	2013	Journal of Bone and Mineral Research	Wöhrle, S. and Henninger, C. and Bonny, O. and Thuery, A. and Beluch, N. and Hynes, N.E. and Guagnano, V. and Sellers, W.R. and Hofmann, F. and Kneissel, M. and Graus Porta, D.
Brånemark system implants and rare disorders: A report of six cases	2013	International Journal of Periodontics and Restorative Dentistry	Friberg, B.
FGF23-induced hypophosphatemia persists in hyp mice deficient in the WNT coreceptor Lrp6	2013	Contributions to Nephrology	Uchihashi, K. and Nakatani, T. and Goetz, R. and Mohammadi, M. and He, X. and Razzaque, M.S.
Nuclear fibroblast growth factor 2 (FGF2) isoforms inhibit bone marrow stromal cell mineralization through FGF23/FGFR/MAPK in vitro	2013	Journal of Bone and Mineral Research	Xiao, L. and Esliger, A. and Hurley, M.M.
Extracellular matrix mineralization in periodontal tissues: Noncollagenous matrix proteins, enzymes, and relationship to hypophosphatasia and X-linked hypophosphatemia	2013	Periodontology 2000	Mckee, M.D. and Hoac, B. and Addison, W.N. and Barros, N.M.T. and Millán, J.L. and Chaussain, C.
Association between compound heterozygous mutations of SLC34A3 and hypercalciuria	2014	Horm. Res. Paediatr.	Abe, Y. and Nagasaki, K. and Watanabe, T. and Abe, T. and Fukami, M.
Serum fibroblast growth factor 23 is a useful marker to distinguish Vitamin D-deficient rickets from hypophosphatemic rickets	2014	Horm. Res. Paediatr.	Kubota, T. and Kitaoka, T. and Miura, K. and Fujiwara, M. and Ohata, Y. and Miyoshi, Y. and Yamamoto, K. and Takeyari, S. and Yamamoto, T. and Namba, N. and Ozono, K.
Therapeutic management of hypophosphatemic rickets from infancy to adulthood.	2014	Endocrine connections	Linglart A and Biosse-Duplan M and Briot K and Chaussain C and Esterle L and Guillaume-Czitrom S and Kamenicky P and Nevoux J and Prié D and Rothenbuhler A and Wicart P and Harvengt P
Abnormal osteopontin and matrix extracellular phosphoglycoprotein localization, and odontoblast differentiation, in X-linked hypophosphatemic teeth.	2014	Connective tissue research	Salmon B and Bardet C and Coyac BR and Baroukh B and Naji J and Rowe PS and Opsahl Vital S and Linglart A and

			Mckee MD and Chaussain C
Constitutive nuclear expression of dentin matrix protein 1 fails to rescue the Dmp1-null phenotype.	2014	The Journal of biological chemistry	Lin S and Zhang Q and Cao Z and Lu Y and Zhang H and Yan K and Liu Y and McKee MD and Qin C and Chen Z and Feng JQ
Exome sequencing reveals a mutation in DMP1 in a family with familial sclerosing bone dysplasia	2014	Bone	Gannagé-Yared, M.-H. and Makrythanasis, P. and Chouery, E. and Sobacchi, C. and Mehawej, C. and Santoni, F.A. and Guipponi, M. and Antonarakis, S.E. and Hamamy, H. and Mégarbané, A.
Osteocyte-specific deletion of Fgfr1 suppresses FGF23	2014	PLoS ONE	Xiao, Z. and Huang, J. and Cao, L. and Liang, Y. and Han, X. and Quarles, L.D.
Oral manifestations of hyperparathyroidism secondary to familial hypophosphatemic rickets	2014	Pediatric Dentistry	Andrews, R.G. and Baumhardt, H. and Martin, B. and Belachew, D. and Reyes-Múgica, M.
Rare Bone Diseases and Their Dental, Oral, and Craniofacial Manifestations	2014	Journal of Dental Research	Foster, B.L. and Ramnitz, M.S. and Gafni, R.I. and Burke, A.B. and Boyce, A.M. and Lee, J.S. and Wright, J.T. and Akintoye, S.O. and Somerman, M.J. and Collins, M.T.
Effects of extracellular phosphate on gene expression in murine osteoblasts	2014	Calcified Tissue International	Rendenbach, C. and Yorgan, T.A. and Heckt, T. and Otto, B. and Baldauf, C. and Jeschke, A. and Streichert, T. and David, J.P. and Amling, M. and Schinke, T.
Dysregulated gene expression in the primary osteoblasts and osteocytes isolated from hypophosphatemic Hyp mice	2014	PLoS ONE	Miyagawa, K. and Yamazaki, M. and Kawai, M. and Nishino, J. and Koshimizu, T. and Ohata, Y. and Tachikawa, K. and Mikuni-Takagaki, Y. and Kogo, M. and Ozono, K. and Michigami, T.
Unusually severe hypophosphatemic rickets caused by a novel and complex re-arrangement of the PHEX gene	2014	American Journal of Medical Genetics, Part A	Pekkarinen, T. and Lorenz-Depiereux, B. and Lohman, M. and Mäkitie, O.
Hypophosphatemic osteomalacia and bone sclerosis caused by a novel homozygous mutation of the FAM20C gene in an elderly man with a mild variant of raine syndrome	2014	Bone	Takeyari, S. and Yamamoto, T. and Kinoshita, Y. and Fukumoto, S. and Glorieux, F.H. and Michigami, T. and Hasegawa, K. and Kitaoka, T. and Kubota, T. and Imanishi, Y. and Shimotsuji, T. and Ozono, K.

Nucleus-targeted Dmp1 transgene fails to rescue dental defects in Dmp1 null mice	2014	International Journal of Oral Science	Lin, S.-X. and Zhang, Q. and Zhang, H. and Yan, K. and Ward, L. and Lu, Y.-B. and Feng, J.-Q.
Vitamin D represses dentin matrix protein 1 in cementoblasts and osteocytes	2014	Journal of Dental Research	Nociti, F.H. and Foster, B.L. and Tran, A.B. and Dunn, D. and Presland, R.B. and Wang, L. and Bhattacharyya, N. and Collins, M.T. and Somerman, M.J.
Osteocyte communication with the kidney via the production of FGF23: Remote control of phosphate homeostasis	2014	Clinical Reviews in Bone and Mineral Metabolism	Ito, N. and Findlay, D.M. and Atkins, G.J.
Treatment of hypophosphataemic rickets in children remains a challenge	2014	Danish Medical Journal	Nielsen, L.H. and Rahbek, E.T. and Beck-Nielsen, S.S. and Christesen, H.T.
Functional analysis of mutant FAM20C in Raine syndrome with FGF23-related hypophosphatemia	2014	Bone	Kinoshita, Y. and Hori, M. and Taguchi, M. and Fukumoto, S.
Diagnostic Exome Sequencing to Elucidate the Genetic Basis of Likely Recessive Disorders in Consanguineous Families	2014	Human Mutation	Makrythanasis, P. and Nelis, M. and Santoni, F.A. and Guipponi, M. and Vannier, A. and Béna, F. and Gimelli, S. and Stathaki, E. and Temtamy, S. and Mégarbané, A. and Masri, A. and Aglan, M.S. and Zaki, M.S. and Bottani, A. and Fokstuen, S. and Gwanmesia, L. and Aliferis, K. and Bustamante Eduardo, M. and Stamoulis, G. and Psoni, S. and Kitsiou-Tzeli, S. and Fryssira, H. and Kanavakis, E. and Al-Allawi, N. and Sefiani, A. and Al Hait, S. and Elalaoui, S.C. and Jalkh, N. and Al-Gazali, L. and Al-Jasmi, F. and Bouhamed, H.C. and Abdalla, E. and Cooper, D.N. and Hamamy, H. and Antonarakis, S.E.
Hearing loss is part of the clinical picture of ENPP1 loss of function mutation	2014	Hormone Research in Paediatrics	Brachet, C. and Mansbach, A.L. and Clerckx, A. and Deltenre, P. and Heinrichs, C.
Upper spine morphology in hypophosphatemic rickets and healthy controls: A radiographic study	2014	European Journal of Orthodontics	Gjørup, H. and Sonnesen, L. and Beck-Nielsen, S.S. and Haubek, D.
Delayed presentation of rickets in a child with labyrinthine aplasia, microtia and microdontia (LAMM) syndrome	2014	Indian Pediatrics	Singh, A. and Tekin, M. and Falcone, M. and Kapoor, S.
PHEX mimetic (SPR4-Peptide) corrects and improves HYP and wild type mice energy-metabolism	2014	PLoS ONE	Zelenchuk, L.V. and Hedge, A.-M. and Rowe, P.S.N.

Medical treatment of children with hypophosphataemic rickets	2014	Ugeskrift for Laeger	Rahbek, E.T. and Nielsen, L.H. and Beck-Nielsen, S.S. and Christesen, H.T.
Identification of two novel mutations in the PHEX gene in Chinese patients with hypophosphatemic rickets/osteomalacia	2014	PLoS ONE	Yue, H. and Yu, J.-B. and He, J.-W. and Zhang, Z. and Fu, W.-Z. and Zhang, H. and Wang, C. and Hu, W.-W. and Gu, J.-M. and Hu, Y.-Q. and Li, M. and Liu, Y.-J. and Zhang, Z.-L.
Vitamin D receptor mutations in patients with hereditary 1,25-dihydroxyvitamin D-resistant rickets	2014	Molecular Genetics and Metabolism	Malloy, P.J. and Tasic, V. and Taha, D. and Tütüncüler, F. and Ying, G.S. and Yin, L.K. and Wang, J. and Feldman, D.
[Genetic approach to nephrolithiasis].	2015	Giornale italiano di nefrologia : organo ufficiale della Societa italiana di nefrologia	Marangella M and Marcuccio C and Vitale C
Mathematical model for bone mineralization.	2015	Frontiers in cell and developmental biology	Komarova SV and Safranek L and Gopalakrishnan J and Ou MJ and McKee MD and Murshed M and Rauch F and Zuhr E
A Novel PHEX Mutation in Japanese Patients with X-Linked Hypophosphatemic Rickets.	2015	Case reports in genetics	Kawahara T and Watanabe H and Omae R and Yamamoto T and Inazu T
[Diseases revealed by the mouth].	2015	Archives de pediatrie : organe officiel de la Societe francaise de pediatrie	Opsahl-Vital S and Gaucher C and Bardet C and Courson F and Linglart A and Chaussain C
Conventional Therapy in Adults With XLH Improves Dental Manifestations, But Not Enthesopathy.	2015	The Journal of clinical endocrinology and metabolism	Econs MJ
Urinary calcium to creatinine ratio: a potential marker of secondary hyperparathyroidism in patients with vitamin D-dependent rickets type 1A.	2015	Endocrine journal	Miyai K and Onishi T and Kashimada K and Hasegawa Y
The in vivo role of DMP-1 and serum phosphate on bone mineral composition	2015	Bone	Maginot, M. and Lin, S. and Liu, Y. and Yuan, B. and Feng, J.Q. and Aswath, P.B.
Phosphate homeostasis and genetic mutations of familial hypophosphatemic rickets	2015	Journal of Pediatric Endocrinology and Metabolism	Razali, N.N. and Hwu, T.T. and Thilakavathy, K.
Novel de novo nonsense mutation of the PHEX gene (p.Lys50Ter) in a Chinese patient with hypophosphatemic rickets	2015	Gene	Huang, Y. and Mei, L. and Pan, Q. and Tan, H. and Quan, Y. and Gui, B. and Chang, J. and Ma, R. and Peng, Y. and Yang, P. and Liang, D. and Wu, L.
Intestinal depletion of NaPi-IIb/Slc34a2 in mice: Renal and hormonal adaptation	2015	Journal of Bone and Mineral Research	Hernando, N. and Myakala, K. and Simona, F. and Knöpfel, T. and Thomas, L. and Murer, H. and Wagner, C.A. and Biber, J.
The bioactive acidic serine- and aspartate-rich motif peptide	2015	Current Protein and Peptide Science	Minamizaki, T. and Yoshiko, Y.
The specific role of FAM20C in dentinogenesis	2015	Journal of Dental Research	Wang, X. and Wang, J. and Liu, Y. and Yuan, B. and

			Ruest, L.B. and Feng, J.Q. and Qin, C.
Dspp-independent effects of transgenic Trps1 overexpression on dentin formation	2015	Journal of Dental Research	Mobley, C.G. and Kuzynski, M. and Zhang, H. and Jani, P. and Qin, C. and Napierala, D.
Glucose metabolic abnormality is associated with defective mineral homeostasis in skeletal disorder mouse model	2015	Science China Life Sciences	Zou, J.H. and Xiong, X.W. and Lai, B.B. and Sun, M. and Tu, X. and Gao, X.
Do ASARM peptides play a role in nephrogenic systemic fibrosis?	2015	American Journal of Physiology - Renal Physiology	Rowe, P.S.N. and Zelenchuk, L.V. and Laurence, J.S. and Lee, P. and Brooks, W.M. and McCarthy, E.T.
Pathogenesis and diagnostic criteria for rickets and osteomalacia—proposal by an expert panel supported by the Ministry of Health, Labour and Welfare, Japan, the Japanese Society for Bone and Mineral Research, and the Japan Endocrine Society	2015	Journal of Bone and Mineral Metabolism	Fukumoto, S. and Ozono, K. and Michigami, T. and Minagawa, M. and Okazaki, R. and Sugimoto, T. and Takeuchi, Y. and Matsumoto, T.
SPR4-peptide alters bone metabolism of normal and HYP mice	2015	Bone	Zelenchuk, L.V. and Hedge, A.-M. and Rowe, P.S.N.
Identification of a novel PHEX mutation in a Chinese family with X-linked hypophosphatemic rickets using exome sequencing	2015	Biological Chemistry	Yuan, L. and Wu, S. and Xu, H. and Xiao, J. and Yang, Z. and Xia, H. and Liu, A. and Hu, P. and Lu, A. and Chen, Y. and Xu, F. and Deng, H.
Age dependent regulation of bone-mass and renal function by the MEPE ASARM-motif	2015	Bone	Zelenchuk, L.V. and Hedge, A.-M. and Rowe, P.S.N.
Enamel and dentin mineralization in familial hypophosphatemic rickets: A micro-CT study	2015	Dentomaxillofacial Radiology	Ribeiro, T.R. and Costa, F.W.G. and Soares, E.C.S. and Williams, J.R. and Fonteles, C.S.R.
Fibroblast Growth Factor 23: A New Dimension to Diseases of Calcium-Phosphorus Metabolism	2015	Veterinary Pathology	Hardcastle, M.R. and Dittmer, K.E.
The pediatric diseases in dental findings	2015	Archives de Pediatrie	Opsahl-Vital, S. and Gaucher, C. and Bardet, C. and Courson, F. and Linglart, A. and Chaussain, C.
Periodontal diseases in patients with vitamin D-resistant rickets	2015	Georgian medical news	Japaridze, N.V. and Margvelashvili, V.V. and Shengelia, M.I. and Chigladze, T.T. and Kalandadze, M.N.
68Ga-DOTATATE for tumor localization in tumor-induced osteomalacia	2016	J. Clin. Endocrinol. Metab.	El-Maouche, D. and Sadowski, S.M. and Papadakis, G.Z. and Guthrie, L. and Cottle-Delisle, C. and Merkel, R. and Millo, C. and Chen, C.C. and Kebebew, E. and Collins, M.T.
Cutaneous skeletal hypophosphatemia syndrome: clinical spectrum, natural history, and treatment.	2016	Osteoporosis international : a journal established as result of cooperation between the	Ovejero D and Lim YH and Boyce AM and Gafni RI and McCarthy E and

		European Foundation for Osteoporosis and the National Osteoporosis Foundation of the USA	Nguyen TA and Eichenfield LF and DeKlotz CM and Guthrie LC and Tosi LL and Thornton PS and Choate KA and Collins MT
Cutaneous skeletal hypophosphatemia syndrome (CSHS) is a multilineage somatic mosaic RASopathy.	2016	Journal of the American Academy of Dermatology	Lim YH and Ovejero D and Derrick KM and Collins MT and Choate KA
FGF2 High Molecular Weight Isoforms Contribute to Osteoarthropathy in Male Mice.	2016	Endocrinology	Meo Burt P and Xiao L and Dealy C and Fisher MC and Hurley MM
Posttranslational processing of FGF23 in osteocytes during the osteoblast to osteocyte transition.	2016	Bone	Yamamoto H and Ramos-Molina B and Lick AN and Prideaux M and Albornoz V and Bonewald L and Lindberg I
1,25-Dihydroxyvitamin D as Monotherapy for XLH: Back to the Future?	2016	Journal of bone and mineral research : the official journal of the American Society for Bone and Mineral Research	Ovejero D and Gafni RI and Collins MT
Clinical, hormonal, biochemical and genetic characteristics of 75 patients with hypophosphatemic rickets	2016	Problemy Endokrinologii	Kulikova, K.S. and Kolodkina, A.A. and Vasiliev, E.V. and Petrov, V.M. and Gorbach, E.N. and Gofman, F.F. and Korkin, A.Y.A. and Petrov, M.A. and Kenis, V.M. and Tiulpakov, A.N.
CRISPR/Cas9-mediated mutation of PHEX in rabbit recapitulates human X-linked hypophosphatemia (XLH)	2016	Human Molecular Genetics	Sui, T. and Yuan, L. and Liu, H. and Chen, M. and Deng, J. and Wang, Y. and Li, Z. and Lai, L.
Hypophosphatemic Rickets in Siblings: A Rare Case Report	2016	Case Reports in Dentistry	Sarat, G. and Priyanka, N. and Prabhat, M.P.V. and Raja Lakshmi, C. and Bhavana, S.M. and Ayesha Thabusum, D.
Phenotype of dent disease in a cohort of Indian children	2016	Indian Pediatrics	Bhardwaj, S. and Thergaonkar, R. and Sinha, A. and Hari, P. and Hi, C. and Bagga, A.
Transgenic expression of Dspp partially rescued the long bone defects of Dmp1-null mice	2016	Matrix Biology	Jani, P.H. and Gibson, M.P. and Liu, C. and Zhang, H. and Wang, X. and Lu, Y. and Qin, C.
Hereditary hypophosphatemia in Norway: A retrospective population-based study of genotypes, phenotypes, and treatment complications	2016	European Journal of Endocrinology	Rafaelsen, S. and Johansson, S. and Ræder, H. and Bjerknes, R.
Family with sequence similarity member 20C is the primary but not the only kinase for the small-integrin-binding ligand N-linked glycoproteins in bone	2016	FASEB Journal	Yang, X. and Yan, W. and Tian, Y. and Ma, P. and Opperman, L.A. and Wang, X.
Hypophosphatemic rickets and pre-eruptive spontaneous dental abscess	2016	Journal of Dentistry for Children	Stinton, N.M. and Uston, K.A. and Davis, C.D.
Sclerostin antibody (Scl-Ab) improves osteomalacia phenotype in dentin matrix protein 1(Dmp1) knockout mice with little	2016	Matrix Biology	Ren, Y. and Han, X. and Jing, Y. and Yuan, B. and Ke, H. and Liu, M. and Feng, J.Q.

impact on serum levels of phosphorus and FGF23			
Expression and inactivation of osteopontin-degrading PHEX enzyme in squamous cell carcinoma	2016	International Journal of Biochemistry and Cell Biology	Neves, R.L. and Chiarantin, G.M.D. and Nascimento, F.D. and Pesquero, J.B. and Nader, H.B. and Tersariol, I.L.S. and McKee, M.D. and Carmona, A.K. and Barros, N.M.T.
A radiological study on intra- and extra-cranial calcifications in adults with X-linked hypophosphatemia and associations with other mineralizing enthesopathies and childhood medical treatment	2016	Orthodontics and Craniofacial Research	Gjørup, H. and Kjaer, I. and Beck-Nielsen, S.S. and Poulsen, M.R. and Haubek, D.
Skeletal muscle, but not cardiovascular function, is altered in a mouse model of autosomal recessive hypophosphatemic rickets	2016	Frontiers in Physiology	Wacker, M.J. and Touchberry, C.D. and Silswal, N. and Brotto, L. and Elmore, C.J. and Bonewald, L.F. and Andresen, J. and Brotto, M.
Conditional Deletion of Murine Fgf23: Interruption of the Normal Skeletal Responses to Phosphate Challenge and Rescue of Genetic Hypophosphatemia	2016	Journal of Bone and Mineral Research	Clinkenbeard, E.L. and Cass, T.A. and Ni, P. and Hum, J.M. and Bellido, T. and Allen, M.R. and White, K.E.
Tumor-induced osteomalacia	2017	Bone Rep.	Florenzano, P. and Gafni, R.I. and Collins, M.T.
Multimodality Image-Guided Cryoablation for Inoperable Tumor-Induced Osteomalacia	2017	J. Bone Miner. Res.	Tella, S.H. and Amalou, H. and Wood, B.J. and Chang, R. and Chen, C.C. and Robinson, C. and Millwood, M. and Guthrie, L.C. and Xu, S. and Levy, E. and Krishnasamy, V. and Gafni, R.I. and Collins, M.T.
Octreotide Is Ineffective in Treating Tumor-Induced Osteomalacia: Results of a Short-Term Therapy	2017	J. Bone Miner. Res.	Ovejero, D. and El-Maouche, D. and Brillante, B.A. and Khosravi, A. and Gafni, R.I. and Collins, M.T.
Tumour-induced osteomalacia	2017	Nat. Rev. Disease Prim.	Minisola, S. and Peacock, M. and Fukumoto, S. and Cipriani, C. and Pepe, J. and Tella, S.H. and Collins, M.T.
Novel screening system for high-affinity ligand of heredity vitamin D-resistant rickets-associated vitamin D receptor mutant R274L using bioluminescent sensor	2017	J. Steroid Biochem. Mol. Biol.	Mano, H. and Nishikawa, M. and Yasuda, K. and Ikushiro, S. and Saito, N. and Sawada, D. and Honzawa, S. and Takano, M. and Kittaka, A. and Sakaki, T.
Tumor-induced osteomalacia in a 3-year-old with unresectable central giant cell lesions	2017	J. Pediatr. Hematol. Oncol.	Crossen, S.S. and Zambrano, E. and Newman, B. and Bernstein, J.A. and Messner, A.H. and

			Bachrach, L.K. and Twist, C.J.
Dental management of patients with X-linked hypophosphatemia.	2017	Restorative dentistry & endodontics	Lee BN and Jung HY and Chang HS and Hwang YC and Oh WM
Renal Fanconi Syndrome and Hypophosphatemic Rickets in the Absence of Xenotropic and Polytropic Retroviral Receptor in the Nephron.	2017	Journal of the American Society of Nephrology : JASN	Ansermet C and Moor MB and Centeno G and Auberson M and Hu DZ and Baron R and Nikolaeva S and Haenzi B and Katanaeva N and Gautschi I and Katanaev V and Rotman S and Koesters R and Schild L and Pradervand S and Bonny O and Firsov D
The Rare Bone Disease Working Group: report from the 2016 American Society for Bone and Mineral Research Annual Meeting.	2017	Bone	Drake MT and Collins MT and Hsiao EC
Hypertension is a characteristic complication of X-linked hypophosphatemia	2017	Endocrine Journal	Nakamura, Y. and Takagi, M. and Takeda, R. and Miyai, K. and Hasegawa, Y.
A mutation in the Dmp1 gene alters phosphate responsiveness in mice	2017	Endocrinology	Ichikawa, S. and Gerard-O'Riley, R.L. and Acton, D. and McQueen, A.K. and Strobel, I.E. and Witcher, P.C. and Feng, J.Q. and Econs, M.J.
Inhibition of fgfr signaling partially rescues hypophosphatemic rickets in hmwfgf2 tg male mice	2017	Endocrinology	Xiao, L. and Du, E. and Homer-Bouthiette, C. and Hurley, M.M.
Tissue-specific mineralization defects in the periodontium of the Hyp mouse model of X-linked hypophosphatemia	2017	Bone	Coyac, B.R. and Falgayrac, G. and Baroukh, B. and Slimani, L. and Sadoine, J. and Penel, G. and Biosse-Duplan, M. and Schinke, T. and Linglart, A. and McKee, M.D. and Chaussain, C. and Bardet, C.
Heritable and acquired disorders of phosphate metabolism: Etiologies involving FGF23 and current therapeutics	2017	Bone	Clinkenbeard, E.L. and White, K.E.
Osteopontin and the dento-osseous pathobiology of X-linked hypophosphatemia	2017	Bone	Boukpessi, T. and Hoac, B. and Coyac, B.R. and Leger, T. and Garcia, C. and Wicart, P. and Whyte, M.P. and Glorieux, F.H. and Linglart, A. and Chaussain, C. and McKee, M.D.
Magnetic Resonance Imaging Features as Surrogate Markers of X-Linked Hypophosphatemic Rickets Activity	2017	Hormone Research in Paediatrics	Lempicki, M. and Rothenbuhler, A. and Merzoug, V. and Franchi-Abella, S. and Chaussain, C. and Adamsbaum, C. and Linglart, A.

Phosphate and Vitamin D Prevent Periodontitis in X-Linked Hypophosphatemia	2017	Journal of Dental Research	Biosse Duplan, M. and Coyac, B.R. and Bardet, C. and Zadikian, C. and Rothenbuhler, A. and Kamenicky, P. and Briot, K. and Linglart, A. and Chaussain, C.
Tumor-induced osteomalacia in association with PTEN-negative Cowden syndrome	2018	Osteoporosis Int.	Berglund, J.A. and Gafni, R.I. and Wodajo, F. and Cowen, E.W. and El-Maouche, D. and Chang, R. and Chen, C.C. and Guthrie, L.C. and Molinolo, A.A. and Collins, M.T.
Soluble Klotho causes hypomineralization in Klotho-deficient mice.	2018	The Journal of endocrinology	Minamizaki T and Konishi Y and Sakurai K and Yoshioka H and Aubin JE and Kozai K and Yoshiko Y
Prevention & control of fluorosis & linked disorders: Developments in the 21(st) Century - Reaching out to patients in the community & hospital settings for recovery.	2018	The Indian journal of medical research	Susheela AK and Toteja GS
Fibroblast growth factor 23 does not directly influence skeletal muscle cell proliferation and differentiation or ex vivo muscle contractility.	2018	American journal of physiology. Endocrinology and metabolism	Avin KG and Vallejo JA and Chen NX and Wang K and Touchberry CD and Brotto M and Dallas SL and Moe SM and Wacker MJ
Eldecalcitol Causes FGF23 Resistance for Pi Reabsorption and Improves Rachitic Bone Phenotypes in the Male Hyp Mouse.	2018	Endocrinology	Kaneko I and Segawa H and Ikuta K and Hanazaki A and Fujii T and Tatsumi S and Kido S and Hasegawa T and Amizuka N and Saito H and Miyamoto KI
Marked alterations in the structure, dynamics and maturation of growth plate likely explain growth retardation and bone deformities of young Hyp mice.	2018	Bone	Fuente R and Gil-Peña H and Claramunt-Taberner D and Hernández-Frías O and Fernández-Iglesias Á and Hermida-Prado F and Anes-González G and Rubio-Aliaga I and Lopez JM and Santos F
Hypophosphatemic rickets: Pathogenesis, diagnosis and treatment	2018	Obesity and Metabolism	Kulikova, K.S. and Tiulpakov, A.N.
Two novel variants of the PHEX gene in patients with X-linked dominant hypophosphatemic rickets and prenatal diagnosis for fetuses in these families	2018	International Journal of Molecular Medicine	Liao, H. and Zhu, H.-M. and Liu, H.-Q. and Li, L.-P. and Liu, S.-L. and Wang, H.
Impaired mineral quality in dentin in X-linked hypophosphatemia	2018	Connective Tissue Research	Coyac, B.R. and Falgayrac, G. and Penel, G. and Schmitt, A. and Schinke, T. and Linglart, A. and McKee, M.D. and Chaussain, C. and Bardet, C.

Qualitative Research to Explore the Patient Experience of X-Linked Hypophosphatemia and Evaluate the Suitability of the BPI-SF and WOMAC® as Clinical Trial End Points	2018	Value in Health	Theodore-Oklota, C. and Bonner, N. and Spencer, H. and Arbuckle, R. and Chen, C.-Y. and Skrinar, A.
Analysis of 2 novel mutations of PHEX gene inducing X-linked dominant hypophosphatemia rickets in 2 families: Two case reports	2018	Medicine (United States)	Gao, Y. and Wang, Z.-M. and Li, X.-L.
Oral symptoms and oral health-related quality of life in people with rare diseases in Germany: A cross-sectional study	2018	International Journal of Environmental Research and Public Health	Wiemann, S. and Baudisch, N.F. and Jordan, R.A. and Kleinheinz, J. and Hanisch, M.
Clinical and genetic characteristics of 15 families with hereditary hypophosphatemia: Novel mutations in PHEX and SLC34A3	2018	PLoS ONE	Acar, S. and BinEssa, H.A. and Demir, K. and Al-Rijjal, R.A. and Zou, M. and Çatli, G. and Anık, A. and Al-Enezi, A.F. and Özışık, S. and Al-Faham, M.S.A. and Abacı, A. and Dündar, B. and Kattan, W.E. and Alsagob, M. and Kavukçu, S. and Tamimi, H.E. and Meyer, B.F. and Böber, E. and Shi, Y.
Burosumab: First Global Approval	2018	Drugs	Lamb, Y.N.
Targeted resequencing of phosphorus metabolism-related genes in 86 patients with hypophosphatemic rickets/osteomalacia	2018	International Journal of Molecular Medicine	Gu, J. and Wang, C. and Zhang, H. and Yue, H. and Hu, W. and He, J. and Fu, W. and Zhang, Z.
A novel de novo mosaic mutation in phex in a korean patient with hypophosphatemic rickets	2018	Annals of Pediatric Endocrinology and Metabolism	Yang, M. and Kim, J. and Yang, A. and Jang, J. and Jeon, T.Y. and Cho, S.Y. and Jin, D.-K.
Defective Mineralization in X-Linked Hypophosphatemia Dental Pulp Cell Cultures	2018	Journal of Dental Research	Coyac, B.R. and Hoac, B. and Chafey, P. and Falgayrac, G. and Slimani, L. and Rowe, P.S. and Penel, G. and Linglart, A. and McKee, M.D. and Chaussain, C. and Bardet, C.
Dentoalveolar Abscesses Not Associated with Caries or Trauma: A Diagnostic Hallmark of Hypophosphatemic Rickets Initially Misdiagnosed as Hypochondroplasia	2018	Head and Neck Pathology	Paredes, S.E.Y. and Segato, R.A.B. and Moreira, L.D. and Moreira, A. and Serrano, K.V.D. and Rodrigues, C.T. and Almeida, L.Y. and León, J.E.
FGF23 Neutralizing Antibody Partially Improves Bone Mineralization Defect of HMWFGF2 Isoforms in Transgenic Female Mice	2018	Journal of Bone and Mineral Research	Xiao, L. and Homer-Bouthiette, C. and Hurley, M.M.
Mutation analysis of four pedigrees affected with hypophosphatemic rickets through targeted next-generation sequencing	2018	Chinese Journal of Medical Genetics	Bai, Y. and Liu, N. and Shao, M. and Qin, G. and Gao, X. and Kong, X.

A Randomized, Double-Blind, Placebo-Controlled, Phase 3 Trial Evaluating the Efficacy of Burosumab, an Anti-FGF23 Antibody, in Adults With X-Linked Hypophosphatemia: Week 24 Primary Analysis	2018	Journal of Bone and Mineral Research	Insogna, K.L. and Briot, K. and Imel, E.A. and Kamenický, P. and Ruppe, M.D. and Portale, A.A. and Weber, T. and Pitukcheewanont, P. and Cheong, H.I. and Jan de Beur, S. and Imanishi, Y. and Ito, N. and Lachmann, R.H. and Tanaka, H. and Perwad, F. and Zhang, L. and Chen, C.-Y. and Theodore-Oklota, C. and Mealiffe, M. and San Martin, J. and Carpenter, T.O.
Burosumab in X-linked hypophosphatemia: a profile of its use in the USA	2018	Drugs and Therapy Perspectives	Lyseng-Williamson, K.A.
Vibrational spectroscopic analysis of hydroxyapatite in HYP mice and individuals with X-linked hypophosphatemia	2018	Therapeutic Advances in Chronic Disease	Amenta, E. and King, H.E. and Petermann, H. and Uskoković, V. and Tommasini, S.M. and Macica, C.M.
Identification of a novel loss-of-function PHEX mutation, Ala720Ser, in a sporadic case of adult-onset hypophosphatemic osteomalacia	2018	Bone	Goljanek-Whysall, K. and Tridimas, A. and McCormick, R. and Russell, N.-J. and Sloman, M. and Sorani, A. and Fraser, W.D. and Hannan, F.M.
Tumor-induced hypophosphatemic osteomalacia caused by a mesenchymal tumor of the mandible managed by a segmental mandibulectomy and microvascular reconstruction with a free fibula flap	2019	Head Neck	Acharya, R.P. and Won, A.M. and Moon, B.S. and Flint, J.H. and Roubaud, M.S. and Williams, M.D. and Hessel, A.C. and Murphy, W.A. and Chambers, M.S. and Gagel, R.F.
Hypophosphatemic osteomalacia associated with adefovir-induced fanconi syndrome initially diagnosed as diabetic kidney disease and vitamin D deficiency	2019	Intern. Med.	Koda, R. and Tsuchida, M. and Iino, N. and Narita, I.
FGF23 and its role in X-linked hypophosphatemia-related morbidity.	2019	Orphanet journal of rare diseases	Beck-Nielsen SS and Mughal Z and Haffner D and Nilsson O and Levtchenko E and Ariceta G and de Lucas Collantes C and Schnabel D and Jandhyala R and Mäkitie O
Hypophosphatemic Rickets.	2019	Pediatric clinics of North America	Bitzan M and Goodyer PR
X-linked hypophosphatemia: Management and treatment prospects.	2019	Joint bone spine	Lambert AS and Zhukouskaya V and Rothenbuhler A and Linglart A
Dent disease: A window into calcium and phosphate transport.	2019	Journal of cellular and molecular medicine	Anglani F and Gianesello L and Beara-Lasic L and Lieske J
Bone and heart health in chronic kidney disease: role of dentin matrix protein 1.	2019	Current opinion in nephrology and hypertension	Martin A

MAPK inhibition and growth hormone: a promising therapy in XLH.	2019	FASEB journal : official publication of the Federation of American Societies for Experimental Biology	Fuente R and Gil-Peña H and Claramunt-Taberner D and Hernández-Frías O and Fernández-Iglesias Á and Alonso-Durán L and Rodríguez-Rubio E and Hermida-Prado F and Anes-González G and Rubio-Aliaga I and Wagner C and Santos F
Familial Vitamin D-dependent rickets Type 2A: A report of two cases with alopecia and oral manifestations.	2019	Journal of oral and maxillofacial pathology : JOMFP	Thakur M
A G protein-coupled, IP3/protein kinase C pathway controlling the synthesis of phosphaturic hormone FGF23.	2019	JCI insight	He Q and Shumate LT and Matthias J and Aydin C and Wein MN and Spatz JM and Goetz R and Mohammadi M and Plagge A and Divieti Pajevic P and Bastepe M
High Incidence of Cranial Synostosis and Chiari I Malformation in Children With X-Linked Hypophosphatemic Rickets (XLHR)	2019	Journal of Bone and Mineral Research	Rothenbuhler, A. and Fadel, N. and Debza, Y. and Bacchetta, J. and Diallo, M.T. and Adamsbaum, C. and Linglart, A. and Di Rocco, F.
Efficacy and safety of burosumab in children aged 1–4 years with X-linked hypophosphataemia: a multicentre, open-label, phase 2 trial	2019	The Lancet Diabetes and Endocrinology	Whyte, M.P. and Carpenter, T.O. and Gottesman, G.S. and Mao, M. and Skrinar, A. and San Martin, J. and Imel, E.A.
Dental findings in fanconi bickel syndrome: Case report	2019	International Journal of Current Research and Review	Nermine, J. and Meriam, B.B. and Emna, B. and Badiaa, J.
C-Terminal Fibroblast Growth Factor-23 Levels in Non-Nutritional Hypophosphatemic Rickets	2019	Indian Journal of Pediatrics	Bharati, J. and Bhatia, D. and Khandelwal, P. and Gupta, N. and Sinha, A. and Khadgawat, R. and Hari, P. and Bagga, A.
Aberrant activation of Wnt signaling pathway altered osteocyte mineralization	2019	Bone	Zhou, Y. and Lin, J. and Shao, J. and Zuo, Q. and Wang, S. and Wolff, A. and Nguyen, D.T. and Rintoul, L. and Du, Z. and Gu, Y. and Peng, Y.Y. and Ramshaw, J.A.M. and Long, X. and Xiao, Y.
Clinical and genetic analysis in a large Chinese cohort of patients with X-linked hypophosphatemia	2019	Bone	Zhang, C. and Zhao, Z. and Sun, Y. and Xu, L. and JiaJue, R. and Cui, L. and Pang, Q. and Jiang, Y. and Li, M. and Wang, O. and He, X. and He, S. and Nie, M. and Xing, X. and Meng, X. and Zhou, X. and Yan, L. and Kaplan, J.M. and Insogna, K.L. and Xia, W.

Spontaneous dental abscesses in hereditary hypophosphataemic rickets: A preventive restorative approach in the primary dentition	2019	Dental Update	Hughes, S.L. and Hingston, E.J.
Burosumab versus conventional therapy in children with X-linked hypophosphataemia: a randomised, active-controlled, open-label, phase 3 trial	2019	The Lancet	Imel, E.A. and Glorieux, F.H. and Whyte, M.P. and Munns, C.F. and Ward, L.M. and Nilsson, O. and Simmons, J.H. and Padidela, R. and Namba, N. and Cheong, H.I. and Pitukcheewanont, P. and Sochett, E. and Högler, W. and Muroya, K. and Tanaka, H. and Gottesman, G.S. and Biggin, A. and Perwad, F. and Mao, M. and Chen, C.-Y. and Skrinar, A. and San Martin, J. and Portale, A.A.
Mutation update and long-term outcome after treatment with active vitamin D3 in Chinese patients with pseudovitamin D-deficiency rickets (PDDR)	2019	Osteoporosis International	Chi, Y. and Sun, J. and Pang, L. and Jiajue, R. and Jiang, Y. and Wang, O. and Li, M. and Xing, X. and Hu, Y. and Zhou, X. and Meng, X. and Xia, W.
Rare diseases with periodontal manifestations	2019	International Journal of Environmental Research and Public Health	Hanisch, M. and Hoffmann, T. and Bohner, L. and Hanisch, L. and Benz, K. and Kleinheinz, J. and Jackowski, J.
Clinical practice recommendations for the diagnosis and management of X-linked hypophosphataemia	2019	Nature Reviews Nephrology	Haffner, D. and Emma, F. and Eastwood, D.M. and Duplan, M.B. and Bacchetta, J. and Schnabel, D. and Wicart, P. and Bockenhauer, D. and Santos, F. and Levtchenko, E. and Harvengt, P. and Kirchhoff, M. and Di Rocco, F. and Chaussain, C. and Brandi, M.L. and Savendahl, L. and Briot, K. and Kamenicky, P. and Rejnmark, L. and Linglart, A.
DMP1 Ablation in the Rabbit Results in Mineralization Defects and Abnormalities in Haversian Canal/Osteon Microarchitecture	2019	Journal of Bone and Mineral Research	Liu, T. and Wang, J. and Xie, X. and Wang, K. and Sui, T. and Liu, D. and Lai, L. and Zhao, H. and Li, Z. and Feng, J.Q.
Oral symptoms and oral health-related quality of life of individuals with x-linked hypophosphatemia	2019	Head and Face Medicine	Hanisch, M. and Bohner, L. and Sabandal, M.M.I. and Kleinheinz, J. and Jung, S.
Co-morbidity with hypertrophic osteoarthropathy: A possible Iron Age Sarmatian case from the Volga steppe of Russia	2019	International Journal of Paleopathology	Loyer, J. and Murphy, E. and Ruppe, M. and Moiseyev, V. and Khartanovich, V. and Zammit, J. and Rottier, S. and Potrakhov, N. and

			Bessonov, V. and Obodovskiy, A.
A rare cause of atraumatic fractures: case series of four patients with tumor-induced osteomalacia	2020	Clin. Diabetes Endocrinol.	Chen, D.W. and Clines, G.A. and Collins, M.T. and Douyon, L. and Choksi, P.U.
Neurofibromatosis type 1 associated with hypophosphatemic osteomalacia due to hypersecretion of fibroblast growth factor 23: A case report	2020	J. Med. Case Rep.	Obo, T. and Koriyama, N. and Tokito, A. and Ogiso, K. and Nishio, Y.
Management of X-linked hypophosphatemia in adults.	2020	Metabolism: clinical and experimental	Lecoq AL and Brandi ML and Linglart A and Kamenický P
Mineralized tissues in hypophosphatemic rickets.	2020	Pediatric nephrology (Berlin, Germany)	Robinson ME and AlQuorain H and Murshed M and Rauch F
X-linked Hypophosphatemia (XLH) Mimicking Rheumatic Disease.	2020	Internal medicine (Tokyo, Japan)	Takase R and Nakano Y and Hasegawa K and Otsuka F
Multidisciplinary patient care in X-linked hypophosphatemic rickets: one challenge, many perspectives.	2020	Wiener medizinische Wochenschrift (1946)	Raimann A and Mindler GT and Kocijan R and Bekes K and Zwerina J and Haeusler G and Ganger R
Hypophosphataemic Rickets: Similar Phenotype of Different Diseases.	2020	Advances in therapy	de la Cerda-Ojeda F and González-Rodríguez JD and Madariaga L and Martínez-Díaz-Guerra G and Matoses-Ruipérez ML
The Osteocyte as the New Discovery of Therapeutic Options in Rare Bone Diseases.	2020	Frontiers in endocrinology	Pathak JL and Bravenboer N and Klein-Nulend J
Physiology of FGF23 and overview of genetic diseases associated with renal phosphate wasting.	2020	Metabolism: clinical and experimental	Bacchetta J and Bardet C and Prié D
X-Linked Hypophosphatemic Rickets Manifesting as Sclerotic Bone Disease and Enthesopathy.	2020	Cureus	Boro H and Singh Naik S and Singh C and Khatiwada S and Khadgawat R
Generalized Arterial Calcification of Infancy: New Insights, Controversies, and Approach to Management.	2020	Current osteoporosis reports	Boyce AM and Gafni RI and Ferreira CR
Giving Credence to the Experience of X-Linked Hypophosphatemia in Adulthood: An Interprofessional Mixed-Methods Study.	2020	Journal of patient-centered research and reviews	Hughes M and Macica C and Meriano C and Doyle M
Active sites of human MEPE-ASARM regulating bone matrix mineralization.	2020	Molecular and cellular endocrinology	Minamizaki T and Sakurai K and Hayashi I and Toshishige M and Yoshioka H and Kozai K and Yoshiko Y
[Biopsychosocial care concept for children with X-chromosomal hypophosphatemia (XLH) : Example of the multiprofessional approach of social pediatric centers].	2020	Bundesgesundheitsblatt, Gesundheitsforschung, Gesundheitsschutz	Schnabel D
FGF23-related hypophosphatemia in patients with low bone mineral density and fragility fractures: challenges in diagnosis and management.	2020	Journal of endocrinological investigation	Indirli R and Guabello G and Longhi M and Niada S and Maruca K and Mora S and Maggioni M and Corbetta S

Role of sodium-dependent Pi transporter/Npt2c on Pi homeostasis in klotho knockout mice different properties between juvenile and adult stages.	2020	Physiological reports	Hanazaki A and Ikuta K and Sasaki S and Sasaki S and Koike M and Tanifuji K and Arima Y and Kaneko I and Shiozaki Y and Tatsumi S and Hasegawa T and Amizuka N and Miyamoto KI and Segawa H
A Novel Presentation of Metaphyseal Chondrodysplasia, Schmid Type with Factor VII Deficiency.	2020	Cureus	Ahmed M and Nasir S and Riaz Hashmi SS and Iqbal Z and Saleem A
Disrupted Protein Expression and Altered Proteolytic Events in Hypophosphatemic Dentin Can Be Rescued by Dentin Matrix Protein 1	2020	Frontiers in Physiology	Guirado, E. and Chen, Y. and Ross, R.D. and Zhang, Y. and Chaussain, C. and George, A.
Hyperphosphatemia with low FGF7 and normal FGF23 and sFRP4 levels in the circulation characterizes pediatric hypophosphatasia	2020	Bone	Whyte, M.P. and Zhang, F. and Wenkert, D. and Mumm, S. and Berndt, T.J. and Kumar, R.
Orthodontic treatment of a nine-year-old patient with hypophosphatemic rickets diagnosed since the age of two: A case report	2020	International Orthodontics	Makrygiannakis, M.A. and Dastoori, M. and Athanasiou, A.E.
Nationwide Turkish cohort study of hypophosphatemic rickets	2020	JCRPE Journal of Clinical Research in Pediatric Endocrinology	Şıklar, Z. and Turan, S. and Bereket, A. and Baş, F. and Güran, T. and Akberzade, A. and Abacı, A. and Demir, K. and Böber, E. and Özbek, M.N. and Kara, C. and Poyrazoğlu, Ş. and Aydın, M. and Kardelen, A. and Tarım, Ö. and Eren, E. and Hatipoğlu, N. and Büyükinan, M. and Akyürek, N. and Çetinkaya, S. and Bayramoğlu, E. and Eklioğlu, B.S. and Uçaktürk, A. and Abalı, S. and Gökşen, D. and Kor, Y. and Ünal, E. and Esen, İ. and Yıldırım, R. and Akın, O. and Çayır, A. and Dilek, E. and Kırel, B. and Anık, A. and Çatlı, G. and Berberoğlu, M.
X-linked hypophosphatemia diagnosed after identification of dental symptoms	2020	Pediatric Dental Journal	Wato, K. and Okawa, R. and Matayoshi, S. and Ogaya, Y. and Nomura, R. and Nakano, K.
Phex gene mutation in a patient with x-linked hypophosphatemic rickets in a developing country	2020	Application of Clinical Genetics	Forero-Delgadillo, J.M. and Cleves, D. and Ochoa, V. and Londoño-Correa, H. and Restrepo, J.M. and Nastasi-Catanese, J.A. and Pachajoa, H.

Development of Enthesopathies and Joint Structural Damage in a Murine Model of X-Linked Hypophosphatemia	2020	Frontiers in Cell and Developmental Biology	Faraji-Bellée, C.-A. and Cauliez, A. and Salmon, B. and Fogel, O. and Zhukouskaya, V. and Benoit, A. and Schinke, T. and Roux, C. and Linglart, A. and Miceli-Richard, C. and Chaussain, C. and Briot, K. and Bardet, C.
Familial hypophosphatemic rickets caused by a PHEX gene mutation accompanied by a NPR2 missense mutation	2020	Journal of Pediatric Endocrinology and Metabolism	Zhao, Y. and Yang, F. and Wang, L. and Che, H.
X-linked hypophosphatemia	2020	Medico e Bambino	Lucchetti, L. and Fintini, D. and Cappa, M. and Emma, F.
Alterations of bone material properties in adult patients with X-linked hypophosphatemia (XLH)	2020	Journal of Structural Biology	Fratzl-Zelman, N. and Gamsjaeger, S. and Blouin, S. and Kocijan, R. and Plasenzotti, P. and Rokidi, S. and Nawrot-Wawrzyniak, K. and Roetzer, K. and Uyanik, G. and Haeusler, G. and Shane, E. and Cohen, A. and Klaushofer, K. and Paschalis, E.P. and Roschger, P. and Fratzl, P. and Zwerina, J. and Zwettler, E.
Validation of a next-generation sequencing (NGS) panel to improve the diagnosis of X-linked hypophosphataemia (XLH) and other genetic disorders of renal phosphate wasting	2020	European Journal of Endocrinology	Thiele, S. and Werner, R. and Stubbe, A. and Hiort, O. and Hoeppner, W.
New Developments in the Treatment of X-Linked Hypophosphataemia: Implications for Clinical Management	2020	Pediatric Drugs	Saraff, V. and Nadar, R. and Högler, W.
Exploring the Burden of X-Linked Hypophosphataemia: An Opportunistic Qualitative Study of Patient Statements Generated During a Technology Appraisal	2020	Advances in Therapy	Ferizović, N. and Marshall, J. and Williams, A.E. and Mughal, M.Z. and Shaw, N. and Mak, C. and Gardiner, O. and Hossain, P. and Upadhyaya, S.
Clinical guidelines for burosumab in the treatment of XLH in children and adolescents: British paediatric and adolescent bone group recommendations	2020	Endocrine Connections	Padidela, R. and Cheung, M.S. and Saraff, V. and Dharmaraj, P.
Identification of a novel variant in the PHEX gene using targeted gene panel sequencing in a 24-month-old boy with hypophosphatemic rickets	2020	Annals of Pediatric Endocrinology and Metabolism	Jo, H.Y. and Shin, J.H. and Kim, H.Y. and Kim, Y.M. and Lee, H. and Bae, M.H. and Park, K.H. and Jang, J.-H. and Kwak, M.J.
Dentoalveolar Defects in the Hyp Mouse Model of X-linked Hypophosphatemia	2020	Journal of Dental Research	Zhang, H. and Chavez, M.B. and Kolli, T.N. and Tan, M.H. and Fong, H. and Chu, E.Y. and Li, Y. and Ren, X. and Watanabe, K. and Kim, D.G. and Foster, B.L.

Long-term dental intervention and laboratory examination in a patient with Vitamin D-dependent rickets type I: A case report	2020	Medicine (United States)	Liu, A.-Q. and Zhang, L.-S. and Guo, H. and Wu, M.-L. and Li, T.-Y. and Xuan, K. and Wei, K.-W.
The international X-linked hypophosphataemia (XLH) registry (NCT03193476): rationale for and description of an international, observational study	2020	Orphanet Journal of Rare Diseases	Padidela, R. and Nilsson, O. and Makitie, O. and Beck-Nielsen, S. and Ariceta, G. and Schnabel, D. and Brandi, M.L. and Boot, A. and Levtchenko, E. and Smyth, M. and Jandhyala, R. and Mughal, Z.
Hypophosphatemic osteosclerosis, hyperostosis, and enthesopathy associated with novel homozygous mutations of DMP1 encoding dentin matrix protein 1 and SPP1 encoding osteopontin: The first digenic SIBLING protein osteopathy?	2020	Bone	Whyte, M.P. and Amalnath, S.D. and McAlister, W.H. and McKee, M.D. and Veis, D.J. and Huskey, M. and Duan, S. and Bijanki, V.N. and Alur, S. and Mumm, S.
Insights into dental mineralization from three heritable mineralization disorders	2020	Journal of Structural Biology	Chavez, M.B. and Kramer, K. and Chu, E.Y. and Thumbigere-Math, V. and Foster, B.L.
Hypophosphatasia in Adults: Clinical Spectrum and Its Association With Genetics and Metabolic Substrates	2020	Journal of Clinical Densitometry	Lefever, E. and Witters, P. and Gielen, E. and Vanclooster, A. and Meersseman, W. and Morava, E. and Cassiman, D. and Laurent, M.R.
Compositional and microhardness findings in tooth affected by X-linked hypophosphatemic rickets	2020	Journal of Clinical and Experimental Dentistry	Carvalho, F.-S.-R. and Feitosa, V.-P. and Fonteles, C.-S.R. and Ribeiro, T.-R. and Araújo, B.-S. and Ayala, A.-P. and Costa, F.-W.-G.
Vitamin D-Resistant Rickets Diagnostics and Treatment Challenges at Muhimbili National Hospital, Tanzania	2020	Case Reports in Endocrinology	Godfrey, E.K. and Mussa, F. and Kazahura, P. and Shoo, A. and Naburi, H. and Manji, K.P.
Biological stenciling of mineralization in the skeleton: Local enzymatic removal of inhibitors in the extracellular matrix	2020	Bone	Reznikov, N. and Hoac, B. and Buss, D.J. and Addison, W.N. and Barros, N.M.T. and McKee, M.D.
Diagnosis, treatment-monitoring and follow-up of children and adolescents with X-linked hypophosphatemia (XLH)	2020	Metabolism: Clinical and Experimental	Rothenbuhler, A. and Schnabel, D. and Högler, W. and Linglart, A.
Hypophosphatemic rickets accelerate chondrogenesis and cell trans-differentiation from TMJ chondrocytes into bone cells via a sharp increase in β-catenin	2020	Bone	Li, H. and Jing, Y. and Zhang, R. and Zhang, Q. and Wang, J. and Martin, A. and Feng, J.Q.
Genetic Ablation of Osteopontin in Osteomalacic Hyp Mice Partially Rescues the Deficient Mineralization Without Correcting Hypophosphatemia	2020	Journal of Bone and Mineral Research	Hoac, B. and Østergaard, M. and Wittig, N.K. and Boukpessi, T. and Buss, D.J. and Chaussain, C. and Birkedal, H. and Murshed, M. and McKee, M.D.
Crossfibrillar mineral tessellation in normal and Hyp mouse bone as revealed by 3D FIB-SEM microscopy	2020	Journal of Structural Biology	Buss, D.J. and Reznikov, N. and McKee, M.D.

Hypophosphatemic Rickets: Diagnostic Approach and current Treatments in Childhood	2020	Osteologie	Höppner, J. and Kornak, U. and Högler, W. and Obermayer-Pietsch, B. and Rutsch, F. and Oheim, R. and Grasemann, C.
High-Phosphate Diet Improved the Skeletal Development of Fam20c -Deficient Mice	2020	Cells Tissues Organs	Zhang, H. and Li, L. and Kesterke, M.J. and Lu, Y. and Qin, C.
Dental manifestations of pseudo-vitamin-D deficiency rickets in a paediatric patient	2021	BMJ Case Rep.	Rahul, M. and Gowthaman, K. and Tewari, N. and Mathur, V.
Atypical presentation of Dent disease in a patient with interstitial Xp11.22 deletion	2021	J. Nephrol.	Drovandi, S. and Servetti, M. and Angeletti, A. and Puliti, A. and Ronchetto, P. and Tassano, E. and Ghiggeri, G.M. and Caridi, G.
Diagnosis and management of X-linked hypophosphatemia in children and adolescent in the Gulf Cooperation Council countries	2021	Arch. Osteoporosis	Al Juraibah, F. and Al Amiri, E. and Al Dubayee, M. and Al Jubeh, J. and Al Kandari, H. and Al Sagheir, A. and Al Shaikh, A. and Beshyah, S.A. and Deeb, A. and Habeb, A. and Mustafa, M. and Zidan, H. and Mughal, M.Z.
Genotype–phenotype analysis, and assessment of the importance of the zinc-binding site in PHEX in Japanese patients with X-linked hypophosphatemic rickets using 3D structure modeling	2021	Bone	Ishihara, Y. and Ohata, Y. and Takeyari, S. and Kitaoka, T. and Fujiwara, M. and Nakano, Y. and Yamamoto, K. and Yamada, C. and Yamamoto, K. and Michigami, T. and Mabe, H. and Yamaguchi, T. and Matsui, K. and Tamada, I. and Namba, N. and Yamamoto, A. and Etoh, J. and Kawaguchi, A. and Kosugi, R. and Ozono, K. and Kubota, T.
Triad signs shown by bone scintigraphy in FGF23-related osteomalacia	2021	QJM	Yamamoto, K. and Honda, H. and Ota, I. and Otsuka, F.
Decreased trabecular bone mass in Col22a1-deficient mice	2021	Cells	Zhao, W. and Wiedemann, P. and Wölfel, E.M. and Neven, M. and Peters, S. and Imhof, T. and Koch, M. and Busse, B. and Amling, M. and Schinke, T. and Yorgan, T.A.
Burosumab treatment for fibrous dysplasia	2021	Bone	Gladding, A. and Szymczuk, V. and Auble, B.A. and Boyce, A.M.
Rickets, elevated fibroblast growth factor-23 and mild anemia: Answers	2021	Pediatr. Nephrol.	VanSickle, J.S. and Srivastava, T. and Monachino, P. and Alon, U.S.

Presentation and non-surgical endodontic treatment of two patients with X-linked hypophosphatemia: a case report	2021	Int Endod J	Bradley, H. and Dutta, A. and Philpott, R.
Effect of high phosphate diet on the formation of dentin in Fam20c-deficient mice	2021	Eur J Oral Sci	Zhang, H. and Xu, Q. and Lu, Y. and Qin, C.
Experience with the targeted next-generation sequencing in the diagnosis of hereditary hypophosphatemic rickets	2021	J. Pediatr. Endocrinol. Metab.	Turan, I. and Erdem, S. and Kotan, L.D. and Dilek, S.O. and Tastan, M. and Gurbuz, F. and Bişgin, A. and Bayazit, A.K. and Topaloglu, A.K. and Yuksel, B.
Impaired 1,25 dihydroxyvitamin D3 action and hypophosphatemia underlie the altered lacuno-canalicular remodeling observed in the Hyp mouse model of XLH	2021	PLoS ONE	Yuan, Y. and Jagga, S. and Martins, J.S. and Rana, R. and Pajevic, P.D. and Liu, E.S.
Ocular Adverse Effects of Infigratinib, a New Fibroblast Growth Factor Receptor Tyrosine Kinase Inhibitor	2021	Ophthalmology	Magone, M.T. and Hartley, I.R. and Fitzgibbon, E. and Bishop, R. and Arango, M. and Moran, S. and Vold, R. and Rivero, J.D. and Pozo, K. and Streit, J. and Roszko, K.L. and Collins, M.T. and Gafni, R.I.
Mineralization Defects in the Primary Dentition Associated With X-Linked Hypophosphatemic Rickets	2021	JBMR Plus	Clayton, D. and Chavez, M.B. and Tan, M.H. and Kolli, T.N. and Giovani, P.A. and Hammersmith, K.J. and Bowden, S.A. and Foster, B.L.
Ca(OH)2 apexification of pulp necroses of the permanent incisors in a case of X-linked hypophosphataemic rickets—the 60-month check-up: A case report	2021	Pediatr. Dent. J.	Lee, J.-S.
Diagnostic performance of 68Ga-DOTATOC PET/CT in tumor-induced osteomalacia	2021	Ann. Nucl. Med.	Kato, A. and Nakamoto, Y. and Ishimori, T. and Hayakawa, N. and Ueda, M. and Temma, T. and Sano, K. and Shimizu, Y. and Saga, T. and Togashi, K.
Maxillomandibular osteotomy healing in a patient with hypophosphatemic rickets	2021	Int. J. Oral Maxillofac. Surg.	Ponto, J. and Farkas, D. and Segal, J.D. and Yusupov, Y.
Periodontal status evaluation in adolescents with hereditary rickets-like diseases	2021	Otsenka sostoyaniya tkanei parodonta u podrostkov s nasledstvennymi rakhitopodobnymi zabolevaniyami	Vislobokova, E.V. and Kiselnikova, L.P. and Lezhnev, D.A. and Murtazaev, S.S. and Sholokhova, N.A.
X-Linked Hypophosphatemic Rickets: Multisystemic Disorder in Children Requiring Multidisciplinary Management.	2021	Frontiers in endocrinology	Baroncelli GI and Mora S
Consensus Recommendations for the Diagnosis and Management of X-Linked Hypophosphatemia in Belgium.	2021	Frontiers in endocrinology	Laurent MR and De Schepper J and Trouet D and Godefroid N and Boros E and Heinrichs C and Bravenboer B and Velkeniers B and Lammens J and Harvengt P and Cavalier E and Kaux JF

			and Lombet J and De Waele K and Verroken C and van Hoeck K and Mortier GR and Levtchenko E and Vande Walle J
Burosumab for Pediatric X-Linked Hypophosphatemia.	2021	Current osteoporosis reports	Imel EA
Genetic basis of hereditary hypophosphataemic rickets and phenotype presentation in children and adults.	2021	Endokrynologia Polska	Tavana N and Thilakavathy K and Kennerson ML and Ting TH
Ectopic Calcification and Hypophosphatemic Rickets: Natural History of ENPP1 and ABCC6 Deficiencies.	2021	Journal of bone and mineral research : the official journal of the American Society for Bone and Mineral Research	Ferreira CR and Kintzinger K and Hackbarth ME and Botschen U and Nitschke Y and Mughal MZ and Baujat G and Schnabel D and Yuen E and Gahl WA and Gafni RI and Liu Q and Huertas P and Khursigara G and Rutsch F
Clinical and molecular characterization of Chilean patients with X-linked hypophosphatemia.	2021	Osteoporosis international : a journal established as result of cooperation between the European Foundation for Osteoporosis and the National Osteoporosis Foundation of the USA	Jiménez M and Ivanovic-Zuvic D and Loureiro C and Carvajal CA and Cavada G and Schneider P and Gallardo E and García C and Gonzalez G and Contreras O and Collins MT and Florenzano P
X-linked hypophosphatemia: The medical expert's challenges and the patient's concerns on their journey with the disease.	2021	Archives de pediatrie : organe officiel de la Societe francaise de pediatrie	Hamdy NAT and Harvengt P and Usardi A
Probing the Scope and Mechanisms of Calcitriol Actions Using Genetically Modified Mouse Models.	2021	JBMR plus	Miao D and Goltzman D
Prospective phenotyping of long-term survivors of generalized arterial calcification of infancy (GACI).	2021	Genetics in medicine : official journal of the American College of Medical Genetics	Ferreira CR and Hackbarth ME and Ziegler SG and Pan KS and Roberts MS and Rosing DR and Whelpley MS and Bryant JC and Macnamara EF and Wang S and Müller K and Hartley IR and Chew EY and Corden TE and Jacobsen CM and Holm IA and Rutsch F and Dikoglu E and Chen MY and Mughal MZ and Levine MA and Gafni RI and Gahl WA
A novel therapeutic strategy for skeletal disorders: Proof of concept of gene therapy for X-linked hypophosphatemia.	2021	Science advances	Zhukouskaya VV and Jauze L and Charles S and Leborgne C and Hilliquin S and Sadoine J and Slimani L and Baroukh B and van Wittenberghe L and Danièle N and Rajas F and Linglart A and Mingozzi F and Chaussain C and Bardet C and Ronzitti G

Burosumab treatment in adults with X-linked hypophosphataemia: 96-week patient-reported outcomes and ambulatory function from a randomised phase 3 trial and open-label extension.	2021	RMD open	Briot K and Portale AA and Brandi ML and Carpenter TO and Cheong HI and Cohen-Solal M and Crowley RK and Eastell R and Imanishi Y and Ing S and Insogna K and Ito N and Jan de Beur S and Javaid MK and Kamenicky P and Keen R and Kubota T and Lachmann RH and Perwad F and Pitukcheewanont P and Ralston SH and Takeuchi Y and Tanaka H and Weber TJ and Yoo HW and Nixon A and Nixon M and Sun W and Williams A and Imel EA
Magnetic resonance imaging is a valuable tool to evaluate the therapeutic efficacy of burosumab in children with X-linked hypophosphatemia.	2021	European journal of endocrinology	Zhukouskaya VV and Mannes I and Chaussain C and Kamenický P and Audrain C and Lambert AS and Nevoux J and Wicart P and Briot K and Di Rocco F and Trabado S and Prié D and Di Somma C and Colao A and Adamsbaum C and Rothenbuhler A and Linglart A
Scx(Lin) cells directly form a subset of chondrocytes in temporomandibular joint that are sharply increased in Dmp1-null mice.	2021	Bone	Ma C and Jing Y and Li H and Wang K and Wang Z and Xu C and Sun X and Kaji D and Han X and Huang A and Feng J
Cementocyte alterations associated with experimentally induced cellular cementum apposition in Hyp mice	2021	Journal of Periodontology	Lira dos Santos, E.J. and Salmon, C.R. and Chavez, M.B. and de Almeida, A.B. and Tan, M.H. and Chu, E.Y. and Sallum, E.A. and Casati, M.Z. and Ruiz, K.G.S. and Kantovitz, K.R. and Foster, B.L. and Nociti Júnior, F.H.
Burosumab for the Treatment of Tumor-Induced Osteomalacia	2021	Journal of Bone and Mineral Research	Jan de Beur, S.M. and Miller, P.D. and Weber, T.J. and Peacock, M. and Insogna, K. and Kumar, R. and Rauch, F. and Luca, D. and Cimms, T. and Roberts, M.S. and San Martin, J. and Carpenter, T.O.
Autosomal recessive hypophosphatemic rickets 1 caused by DMP1 mutation: one pedigree study	2021	Chinese Journal of Endocrinology and Metabolism	Gao, L. and Hu, Y. and Zhang, Z.
X-linked hypophosphatemia and burosumab: Practical clinical points from the French experience	2021	Joint Bone Spine	Bacchetta, J. and Rothenbuhler, A. and Gueorguieva, I. and Kamenicky, P. and Salles,

			J.-P. and Briot, K. and Linglart, A.
A 5-year-old girl with bony deformities and disproportionate short stature: Answers	2021	Pediatric Nephrology	Darshan, R. and Krishnamurthy, S. and Deepthi, B. and Karunakar, P. and Chidambaram, A.C. and Archana, A.
Inherited tubulopathies of the kidney insights from genetics	2021	Clinical Journal of the American Society of Nephrology	Downie, M.L. and Lopez Garcia, S.C. and Kleta, R. and Bockenhauer, D.
Effects of Active Vitamin D or FGF23 Antibody on Hyp Mice Dentoalveolar Tissues	2021	Journal of Dental Research	Lira dos Santos, E.J. and Chavez, M.B. and Tan, M.H. and Mohamed, F.F. and Kolli, T.N. and Foster, B.L. and Liu, E.S.
Outcome of primary tubular tubulopathies diagnosed in pediatric age	2021	Nefrologia	Gómez, C.J.B. and Gil-Peña, H. and Álvarez, F.A.O. and Rodríguez, F.S.
Impact of Early Conventional Treatment on Adult Bone and Joints in a Murine Model of X-Linked Hypophosphatemia	2021	Frontiers in Cell and Developmental Biology	Cauliez, A. and Zhukouskaya, V.V. and Hilliquin, S. and Sadoine, J. and Slimani, L. and Miceli-Richard, C. and Briot, K. and Linglart, A. and Chaussain, C. and Bardet, C.
X-linked hypophosphatemic osteomalacia with PHEX mutation presenting late in Pakistan	2021	Annals of Medicine and Surgery	Zehra, N. and Jafri, L. and Kirmani, S. and Khan, A.H.
Osteocytes but not osteoblasts directly build mineralized bone structures	2021	International Journal of Biological Sciences	Wang, K. and Ren, Y. and Lin, S. and Jing, Y. and Ma, C. and Wang, J. and Yuan, X.B. and Han, X. and Zhao, H. and Wang, Z. and Zheng, M. and Xiao, Y. and Chen, L. and Olsen, B.R. and Feng, J.Q.
X-linked hypophosphatemic rickets: A diagnostic and therapeutic challenge	2021	Iatreia	Guerrero-Tinoco, G.A. and García-Bermejo, R. and Cardona-Orozco, E.J.
Interim Analysis of a Phase 2 Open-Label Trial Assessing Burosumab Efficacy and Safety in Patients With Tumor-Induced Osteomalacia	2021	Journal of Bone and Mineral Research	Imanishi, Y. and Ito, N. and Rhee, Y. and Takeuchi, Y. and Shin, C.S. and Takahashi, Y. and Onuma, H. and Kojima, M. and Kanematsu, M. and Kanda, H. and Seino, Y. and Fukumoto, S.
Oral health-related quality of life in X-linked hypophosphataemia and osteogenesis imperfecta	2021	Journal of Oral Rehabilitation	Gjørup, H. and Beck-Nielsen, S.S. and Hald, J.D. and Haubek, D.
A pathogenic phex variant (C.1483-1g>c) in a Korean patient with x-linked hypophosphatemic rickets	2021	Annals of Pediatric Endocrinology and Metabolism	Jeong, I.H. and Yoo, J.-H. and Kim, N.
A Novel Synonymous Variant of PHEX in a Patient with X-Linked Hypophosphatemia	2022	Calcif. Tissue Int.	Ma, X. and Pang, Q. and Zhang, Q. and Jiang, Y. and Wang, O. and Li, M. and Xing, X. and Xia, W.

Growth pattern in children with X-linked hypophosphatemia treated with burosumab and growth hormone	2022	Orphanet J. Rare Dis.	Ertl, D.-A. and Le Lorier, J. and Gleiss, A. and Trabado, S. and Bensignor, C. and Audrain, C. and Zhukouskaya, V. and Coutant, R. and Berkenou, J. and Rothenbuhler, A. and Haeusler, G. and Linglart, A.
Whole exome sequencing identifies two novel variants in PHEX and DMP1 in Malaysian children with hypophosphatemic rickets	2022	Ital. J. Pediatr.	Tavana, N. and Ting, T.H. and Lai, K. and Kennerson, M.L. and Thilakavathy, K.
Growth hormone treatment improves final height in children with X-linked hypophosphatemia	2022	Orphanet J. Rare Dis.	André, J. and Zhukouskaya, V.V. and Lambert, A.-S. and Salles, J.-P. and Mignot, B. and Bardet, C. and Chaussain, C. and Rothenbuhler, A. and Linglart, A.
Determination of FGF23 Levels for the Diagnosis of FGF23-Mediated Hypophosphatemia	2022	J. Bone Miner. Res.	Hartley, I.R. and Gafni, R.I. and Roszko, K.L. and Brown, S.M. and de Castro, L.F. and Saikali, A. and Ferreira, C.R. and Gahl, W.A. and Pacak, K. and Blau, J.E. and Boyce, A.M. and Salusky, I.B. and Collins, M.T. and Florenzano, P.
Rickets guidance: part II—management	2022	Pediatr. Nephrol.	Haffner, D. and Leifheit-Nestler, M. and Grund, A. and Schnabel, D.
Efficacy of Burosumab in Adults with X-linked Hypophosphatemia (XLH): A Post Hoc Subgroup Analysis of a Randomized Double-Blind Placebo-Controlled Phase 3 Study	2022	Calcif. Tissue Int.	Brandi, M.L. and Jan de Beur, S. and Briot, K. and Carpenter, T. and Cheong, H.I. and Cohen-Solal, M. and Crowley, R.K. and Eastell, R. and Imanishi, Y. and Imel, E.A. and Ing, S.W. and Insogna, K. and Ito, N. and Javaid, K. and Kamenicky, P. and Keen, R. and Kubota, T. and Lachmann, R.H. and Perwad, F. and Pitukcheewanont, P. and Portale, A. and Ralston, S.H. and Tanaka, H. and Weber, T.J. and Yoo, H.-W. and Sun, W. and Williams, A. and Nixon, A. and Takeuchi, Y.
Burosumab Treatment for Autosomal Recessive Hypophosphatemic Rickets Type 1 (ARHR1)	2022	J. Clin. Endocrinol. Metab.	Bai, X. and Levental, M. and Karaplis, A.C.

Catalysis-Independent ENPP1 Protein Signaling Regulates Mammalian Bone Mass	2022	J. Bone Miner. Res.	Zimmerman, K. and Liu, X. and von Kroge, S. and Stabach, P. and Lester, E.R. and Chu, E.Y. and Srivastava, S. and Somerman, M.J. and Tommasini, S.M. and Busse, B. and Schinke, T. and Carpenter, T.O. and Oheim, R. and Braddock, D.T.
A Case of X-Linked Hypophosphatemic Rickets with Dentin Dysplasia in Mandibular Third Molars	2022	Child.	Okawa, R. and Hamada, M. and Takagi, M. and Matayoshi, S. and Nakano, K.
Infigratinib Reduces Fibroblast Growth Factor 23 (FGF23) and Increases Blood Phosphate in Tumor-Induced Osteomalacia	2022	JBMR Plus	Hartley, I.R. and Roszko, K.L. and Li, X. and Pozo, K. and Streit, J. and del Rivero, J. and Magone, M.T. and Smith, M.R. and Vold, R. and Dambkowski, C.L. and Collins, M.T. and Gafni, R.I.
X-linked Hypophosphatemic Rickets: Awareness, Knowledge, and Practice of Pediatric Endocrinologists in Arab Countries	2022	J. Pediatr. Genet.	Deeb, A. and Juraibah, F.A. and Dubayee, M.A. and Habeb, A.
Safety and Efficacy of Burosumab in Pediatric Patients With X-Linked Hypophosphatemia: A Phase 3/4 Open-Label Trial	2022	J. Endocr. Soc.	Namba, N. and Kubota, T. and Muroya, K. and Tanaka, H. and Kanematsu, M. and Kojima, M. and Orihara, S. and Kanda, H. and Seino, Y. and Ozono, K.
Persistent Lower Limb Deformities Despite Amelioration of Rickets in X-Linked Hypophosphatemia (XLH) - A Prospective Observational Study	2022	Front. Endocrinol.	Mindler, G.T. and Stauffer, A. and Kranzl, A. and Penzkofer, S. and Ganger, R. and Radler, C. and Haeusler, G. and Raimann, A.
Oral health-related quality of life in patients with X-linked hypophosphatemia: a qualitative exploration	2022	Endocr. Connect.	Nguyen, C. and Celestin, E. and Chambolle, D. and Linglart, A. and Duplan, M.B. and Chaussain, C. and Friedlander, L.
Prevalence of Enthesopathies in Adults with X-linked Hypophosphatemia: Analysis of Risk Factors	2022	J. Clin. Endocrinol. Metab.	Herrou, J. and Picaud, A.S. and Lassalle, L. and Pacot, L. and Chaussain, C. and Merzoug, V. and Hervé, A. and Gadion, M. and Rothenbuhler, A. and Kamenický, P. and Roux, C. and Linglart, A. and Duplan, M.B. and Briot, K.
Autosomal recessive hypophosphatemic rickets type 2; a novel mutation in the ENPP1 gene	2022	Turk. J. Pediatr.	Bitkin, E.Ç. and Aymelek, H.S.

Interdisciplinary management of FGF23-related phosphate wasting syndromes: a Consensus Statement on the evaluation, diagnosis and care of patients with X-linked hypophosphataemia.	2022	Nature reviews. Endocrinology	Trombetti A and Al-Daghri N and Brandi ML and Cannata-Andía JB and Cavalier E and Chandran M and Chaussain C and Cipullo L and Cooper C and Haffner D and Harvengt P and Harvey NC and Javaid MK and Jiwa F and Kanis JA and Laslop A and Laurent MR and Linglart A and Marques A and Mindler GT and Minisola S and Yerro MCP and Rosa MM and Seefried L and Vlaskovska M and Zanchetta MB and Rizzoli R
X-Linked Kidney Disorders in Women.	2022	Seminars in nephrology	Quinlan C and Rheault MN
Osteocytes and the pathogenesis of hypophosphatemic rickets.	2022	Frontiers in endocrinology	Yamazaki M and Michigami T
Impact of X-Linked Hypophosphatemia on Muscle Symptoms.	2022	Genes	Romagnoli C and Iantomasi T and Brandi ML
Growth hormone therapy in HHRH.	2022	Bone reports	Filler G and Schott C and Salerno FR and Ens A and McIntyre CW and Díaz González de Ferris ME and Stein R
X-linked hypophosphatemic rickets: Orthodontic considerations and management. A case report.	2022	Journal of orthodontics	Gibson C and Mubeen S and Evans R
Dramatic Transformation After Burosumab in a Young Boy With X-linked Hypophosphatemia: A Life-Changing Saga.	2022	Cureus	Baradhi K
Letter to the Editor From Yamamoto et al: "Whole Body, Whole Life, Whole Family: Patients' Perspectives on X-Linked Hypophosphatemia".	2022	Journal of the Endocrine Society	Yamamoto K and Omura D and Soga K and Obika M and Otsuka F
The Contribution of Deleterious Rare Alleles in ENPP1 and Osteomalacia Causative Genes to Atypical Femoral Fracture.	2022	The Journal of clinical endocrinology and metabolism	Furukawa H and Oka S and Kondo N and Nakagawa Y and Shiota N and Kumagai K and Ando K and Takeshita T and Oda T and Takahashi Y and Izawa K and Iwasaki Y and Hasegawa K and Arino H and Minamizaki T and Yoshikawa N and Takata S and Yoshihara Y and Tohma S
Expert consensus on diagnosis, treatment and management on X-linked hypophosphatemic rickets in children	2022	Zhonghua er ke za zhi / Chinese Journal of Pediatrics	Luo, X.
Identification of six novel variants from nine Chinese families with hypophosphatemic rickets	2022	BMC Medical Genomics	Cao, Y. and You, Y. and Wang, Q. and Ren, X. and Li, S. and Li, L. and Xia, W. and Guan, X. and Yang,

			T. and Ikegawa, S. and Wang, Z. and Zhao, X.
Mineral tessellation in bone and the stenciling principle for extracellular matrix mineralization	2022	Journal of Structural Biology	McKee, M.D. and Buss, D.J. and Reznikov, N.
Clinical practice guidelines for paediatric X-linked hypophosphataemia in the era of burosumab	2022	Journal of Paediatrics and Child Health	Sandy, J.L. and Simm, P.J. and Biggin, A. and Rodda, C.P. and Wall, C.-L. and Siafarikas, A. and Munns, C.F.
Prevention of Hypomineralization In Auditory Ossicles of Vitamin D Receptor (Vdr) Deficient Mice	2022	Frontiers in Endocrinology	Delsmann, M.M. and Peichl, J. and Yorgan, T.A. and Beil, F.T. and Amling, M. and Demay, M.B. and Rolvien, T.
An Expert Perspective on Phosphate Dysregulation With a Focus on Chronic Hypophosphatemia	2022	Journal of Bone and Mineral Research	Aljuraibah, F. and Bacchetta, J. and Brandi, M.L. and Florenzano, P. and Javaid, M.K. and Mäkitie, O. and Raimann, A. and Rodriguez, M. and Siggelkow, H. and Tiosano, D. and Vervloet, M. and Wagner, C.A.
Whole-exome sequencing and variant spectrum in children with suspected inherited renal tubular disorder: the East India Tubulopathy Gene Study	2022	Pediatric Nephrology	Sinha, R. and Pradhan, S. and Banerjee, S. and Jahan, A. and Akhtar, S. and Pahari, A. and Raut, S. and Parakh, P. and Basu, S. and Srivastava, P. and Nayak, S. and Thenral, S.G. and Ramprasad, V. and Ashton, E. and Bockenhauer, D. and Mandal, K.
Function of PHEX mutations p.Glu145* and p.Trp749Arg in families with X-linked hypophosphatemic rickets by the negative regulation mechanism on FGF23 promoter transcription	2022	Cell Death and Disease	Gan, Y.-M. and Zhang, Y.-P. and Ruan, D.-D. and Huang, J.-B. and Zhu, Y.-B. and Lin, X.-F. and Xiao, X.-P. and Cheng, Q. and Geng, Z.-B. and Liao, L.-S. and Tang, F.-Q. and Luo, J.-W.
Guideline for the diagnosis and management of hypophosphatemic rickets / osteomalacia	2022	Chinese Journal of Endocrinology and Metabolism	Chao, X. and Weibo, X. and Jiajun, Z.
The efficacy and safety of different doses of calcitriol combined with neutral phosphate in X-linked hypophosphatemia: a prospective study	2022	Osteoporosis International	Jin, C. and Zhang, C. and Ni, X. and Zhao, Z. and Xu, L. and Wu, B. and Chi, Y. and Jiajue, R. and Jiang, Y. and Wang, O. and Li, M. and Xing, X. and Meng, X. and Xia, W.
Bone Matrix Mineralization and Response to Burosumab in Adult Patients With X-Linked Hypophosphatemia: Results From the Phase 3, Single-Arm International Trial	2022	Journal of Bone and Mineral Research	Fratzl-Zelman, N. and Hartmann, M.A. and Gamsjaeger, S. and Rokidi, S. and Paschalis, E.P. and Blouin, S. and Zwerina, J.
Genetic and clinical profile of patients with hypophosphatemic rickets	2022	European Journal of Medical Genetics	Marik, B. and Bagga, A. and Sinha, A. and

			Khandelwal, P. and Hari, P. and Sharma, A.
Diagnostic yield of clinical exome sequencing as a first-tier genetic test for the diagnosis of genetic disorders in pediatric patients: results from a referral center study	2022	Human Genetics	Mergnac, J.-P. and Wiedemann, A. and Chery, C. and Ravel, J.-M. and Namour, F. and Guéant, J.-L. and Feillet, F. and Oussalah, A.
Sustained Efficacy and Safety of Burosumab, a Monoclonal Antibody to FGF23, in Children with X-Linked Hypophosphatemia	2022	Journal of Clinical Endocrinology and Metabolism	Linglart, A. and Imel, E.A. and Whyte, M.P. and Portale, A.A. and Högler, W. and Boot, A.M. and Padidela, R. and Van't Hoff, W. and Gottesman, G.S. and Chen, A. and Skrinar, A. and Scott Roberts, M. and Carpenter, T.O.
Clinical practice recommendations for the diagnosis and treatment of X-linked hypophosphatemia: A consensus based on the ADAPTE method	2022	Medicina Clinica	González-Lamuño, D. and Lorente Rodríguez, A. and Luis Yanes, M.I. and Marín-del Barrio, S. and Martínez Díaz-Guerra, G. and Peris, P.
PHEXL222P Mutation Increases Phex Expression in a New ENU Mouse Model for XLH Disease	2022	Genes	El Hakam, C. and Parenté, A. and Baraige, F. and Magnol, L. and Forestier, L. and Di Meo, F. and Blanquet, V.
Dent-2 disease with a Bartter-like phenotype caused by the Asp631Glu mutation in the OCRL gene	2022	BMC Nephrology	Drosataki, E. and Maragkou, S. and Dermitzaki, K. and Stavrakaki, I. and Lygerou, D. and Latsoudis, H. and Pleros, C. and Petrakis, I. and Zaganas, I. and Stylianou, K.
Dental impact of anti-fibroblast growth factor 23 therapy in X-linked hypophosphatemia	2023	Int J Oral Sci	Lira Dos Santos, E.J. and Nakajima, K. and Po, J. and Hanai, A. and Zhukouskaya, V. and Biosse Duplan, M. and Linglart, A. and Shimada, T. and Chaussain, C. and Bardet, C.
Misdiagnosed metabolic bone abnormality: a case report	2023	J. Med. Case Rep.	Alsabri, M. and Street, H. and Sircy, A. and Labib, B.
Electronic reporting of rare endocrine conditions within a clinical network: results from the EuRRECa project	2023	Endocr. Connect.	Ali, S.R. and Bryce, J. and Priego-Zurita, A.L. and Cherenko, M. and Smythe, C. and de Rooij, T.M. and Cools, M. and Danne, T. and Katugampola, H. and Dekkers, O.M. and Hiort, O. and Linglart, A. and Netchine, I. and Nordenstrom, A. and Attila, P. and Persani, L. and Reisch, N. and Smyth, A. and Sumnik, Z. and

			Taruscio, D. and Visser, W.E. and Pereira, A.M. and Appelman-Dijkstra, N.M. and Ahmed, S.F.
Contributions of increased osteopontin and hypophosphatemia to dentoalveolar defects in osteomalacic Hyp mice	2023	Bone	Mohamed, F.F. and Hoac, B. and Phanrungsuwan, A. and Tan, M.H. and Giovani, P.A. and Ghiba, S. and Murshed, M. and Foster, B.L. and McKee, M.D.
Sclerostin antibody improves alveolar bone quality in the Hyp mouse model of X-linked hypophosphatemia (XLH)	2023	Int J Oral Sci	Carpenter, K.A. and Alkhatib, D.O. and Dulion, B.A. and Guirado, E. and Patel, S. and Chen, Y. and George, A. and Ross, R.D.
Adult height improved over decades in patients with X-linked hypophosphatemia: a cohort study	2023	Eur. J. Endocrinol.	Boros, E. and Ertl, D.-A. and Berkenou, J. and Audrain, C. and Lecoq, A.L. and Kamenicky, P. and Briot, K. and Amouroux, C. and Zhukouskaya, V. and Gueorguieva, I. and Mignot, B. and Girerd, B. and Porquet Bordes, V. and Salles, J.P. and Edouard, T. and Coutant, R. and Bacchetta, J. and Linglart, A. and Rothenbuhler, A.
Sex differences of burosumab in children with X-linked hypophosphataemic rickets	2023	Pediatr. Nephrol.	Filler, G. and Tremblay, O. and Chen, E. and Huang, S.S.H. and Stein, R.
Non-invasive prenatal testing (NIPT): Combination of copy number variant and gene analyses using an “in-house” target enrichment next generation sequencing—Solution for non-centralized NIPT laboratory?	2023	Prenat. Diagn.	Faldynová, L. and Walczysková, S. and Černá, D. and Kudrejová, M. and Hilscherová, Š. and Kaniová, R. and Širůčková, S.
Mineral tessellation in mouse enthesis fibrocartilage, Achilles tendon, and Hyp calcifying enthesopathy: A shared 3D mineralization pattern	2023	Bone	Buss, D.J. and Rechav, K. and Reznikov, N. and McKee, M.D.
Clinical, genetic, and structural characterization of a novel TUBB4B tubulinopathy	2023	Mol. Genet. Metab. Rep.	McFadden, J.R. and Tolete, C.D.P. and Huang, Y. and Macnamara, E. and Sept, D. and Nesterova, G. and Gahl, W.A. and Sackett, D.L. and Malicdan, M.C.V.
Fibroblast growth factor 23 levels in cord and peripheral blood during early neonatal period as possible predictors of affected offspring of X-linked hypophosphatemic rickets: Report of three female cases from two pedigrees	2023	J. Pediatr. Endocrinol. Metab.	Nabeshima, Y. and Sato, T. and Zukeran, H. and Komatsu, R. and Nakano, S. and Ichihashi, Y. and Tominaga, T. and Miwa, M. and Amano, N. and Ishii, T. and Hasegawa, T.

Uncovering genetic causes of hypophosphatemia	2023	J. Intern. Med. (GBR)	Puente-Ruiz, N. and Docio, P. and Unzueta, M.T.G. and Lavín, B.A. and Maiztegi, A. and Vega, A.I. and Piedra, M. and Riancho-Zarrabeitia, L. and Mateos, F. and Gonzalez-Lamuño, D. and Valero, C. and Riancho, J.A.
First report in Argentina of a pathogenic DMP1 variant associated with autosomal recessive hypophosphatemic rickets	2023	Arch. Argent. Pediatr.	Bastida, G. and Ramírez, F. and Exeni, G. and Costa, M. and Ávilaa, S.A.
The First Compound Heterozygous Mutations of DMP1 Causing Rare Autosomal Recessive Hypophosphatemic Rickets Type 1	2023	J. Clin. Endocrinol. Metab.	Ni, X. and Gong, Y. and Jiang, Y. and Li, X. and Pang, Q. and Liu, W. and Chi, Y. and Jiajue, R. and Wang, O. and Li, M. and Xing, X. and Xia, W.
Utility of Multimodality Approach Including Systemic FGF23 Venous Sampling in Localizing Phosphaturic Mesenchymal Tumors	2023	J. Endocr. Soc.	Kato, H. and Koga, M. and Kinoshita, Y. and Hidaka, N. and Hoshino, Y. and Takashi, Y. and Arai, M. and Kobayashi, H. and Katsura, M. and Nakamoto, Y. and Makise, N. and Ushiku, T. and Hoshi, K. and Nangaku, M. and Makita, N. and Fukumoto, S. and Ito, N.
Rare PHEX intron variant causes complete and severe phenotype in a family with hypophosphatemic rickets: A case report	2023	J. Pediatr. Endocrinol. Metab.	Aiello, F. and Pasquali, D. and Baronio, F. and Cassio, A. and Rossi, C. and Di Fraia, R. and Carotenuto, R. and Digitale, L. and Festa, A. and Luongo, C. and Maltoni, G. and Schiano Di Cola, R. and Del Giudice, E.M. and Grandone, A.
CLINICAL FEATURES, DIAGNOSTICS AND TREATMENT OF FGF23 SECRETING TUMORS: SERIES OF 40 CLINICAL CASES	2023	КЛИНИЧЕСКИЕ ПРОЯВЛЕНИЯ, ПРИНЦИПЫ ДИАГНОСТИКИ И ЛЕЧЕНИЯ ФОСФАТУРИЧЕСКИХ МЕЗЕНХИМАЛЬНЫХ ОПУХОЛЕЙ, СЕКРЕТИРУЮЩИХ ФАКТОР РОСТА ФИБРОБЛАСТОВ 23: РЕЗУЛЬТАТЫ НАБЛЮДЕНИЯ 40 СЛУЧАЕВ	Gronskaia, S.A. and Belaya, Z.E. and Rozhinskaya, L.Y. and Melnichenko, G.A. and Dubovitskaya, T.A. and Mamedova, E.O. and Rodionova, S.S. and Buklemishev, Y.V. and Pigarova, E.A. and Degtyarev, M.V. and Babaeva, D.M. and Vladimirova, V.P. and Tarbaeva, N.V. and Serzhenko, S.S. and Grigoriev, A.Y. and Dzeranova, L.K. and Karpenko, V.Y. and Karasev, A.L. and Fedotov, R.N. and Uliyanova, I.N. and Toroptsova, N.V. and Lesnyak, O.M. and

			Mokrysheva, N.G. and Dedov, I.I.
FGF23 tumor induced osteomalacia with localization of neoplasm in the tympanic cavity	2023	FRF23-indutsirovannaya osteomalyatsiya opukholevogo geneza s lokalizatsiei novoobrazovaniya v barabannoi polosti	Ovchinnikov, A.Y. and Khon, E.M. and Bakotina, A.V. and Miroshnichenko, N.A. and Gronskaia, S.A. and Belaya, Z.E.
Asia-Pacific Consensus Recommendations on X-Linked Hypophosphatemia: Diagnosis, Multidisciplinary Management, and Transition From Pediatric to Adult Care.	2023	JBMR plus	Munns CF and Yoo HW and Jalaludin MY and Vasanwala R and Chandran M and Rhee Y and But WM and Kong AP and Su PH and Numbenjapon N and Namba N and Imanishi Y and Clifton-Bligh RJ and Luo X and Xia W
Hereditary dentin defects with systemic diseases.	2023	Oral diseases	Su T and Zhu Y and Wang X and Zhu Q and Duan X
X-Linked Hypophosphatemia: Does Targeted Therapy Modify Dental Impairment?	2023	Journal of clinical medicine	Abdullah A and Wuersching SN and Kollmuss M and Poxleitner P and Dewenter I and Brandenburg LS and Steybe D and Fegg FN and Smolka W and Otto S and Obermeier KT
X-linked hypophosphatemia, fibroblast growth factor 23 signaling, and craniosynostosis.	2023	Experimental biology and medicine (Maywood, N.J.)	Grimbly C and Graf D and Ward LM and Alexander RT
Benefit of burosumab in adults with X-linked hypophosphataemia (XLH) is maintained with long-term treatment.	2023	RMD open	Kamenicky P and Briot K and Brandi ML and Cohen-Solal M and Crowley RK and Keen R and Kolta S and Lachmann RH and Lecoq AL and Ralston SH and Walsh JS and Rylands AJ and Williams A and Sun W and Nixon A and Nixon M and Javaid MK
The odontoblastic differentiation of dental mesenchymal stem cells: molecular regulation mechanism and related genetic syndromes.	2023	Frontiers in cell and developmental biology	Pan H and Yang Y and Xu H and Jin A and Huang X and Gao X and Sun S and Liu Y and Liu J and Lu T and Wang X and Zhu Y and Jiang L
Research progress on renal calculus associate with inborn error of metabolism.	2023	Zhejiang da xue xue bao. Yi xue ban = Journal of Zhejiang University. Medical sciences	Song Y and Zhao C and Li D
Ossification of the Posterior Longitudinal Ligament Caused by X-linked Hypophosphatemia.	2023	JMA journal	Yamamoto K and Hasegawa K and Yasuhara T and Otsuka F

Patient-reported outcomes measures of X-linked hypophosphataemia participants: findings from a prospective cohort study in the UK	2023	Orphanet Journal of Rare Diseases	Cole, S. and Sanchez-Santos, M.T. and Kolovos, S. and Javaid, M.K. and Pinedo-Villanueva, R.
Self-Administration of Burosumab in Children and Adults with X-Linked Hypophosphataemia in Two Open-Label, Single-Arm Clinical Studies	2023	Advances in Therapy	Kubota, T. and Namba, N. and Tanaka, H. and Muroya, K. and Imanishi, Y. and Takeuchi, Y. and Kanematsu, M. and Sun, W. and Seino, Y. and Ozono, K.
The contribution of a novel PHEX gene mutation to X-linked hypophosphatemic rickets: a case report and an analysis of the gene mutation dosage effect in a rat model	2023	Frontiers in Endocrinology	Chen, X. and Cai, C. and Lun, S. and Ye, Q. and Pan, W. and Chen, Y. and Wu, Y. and Feng, T. and Su, F. and Ma, C. and Luo, J. and Liu, M. and Ma, G.
Genotype-phenotype Description of Vitamin D-dependent Rickets 1A: CYP27B1 p.(Ala129Thr) Variant Induces a Milder Disease	2023	Journal of Clinical Endocrinology and Metabolism	Meaux, M.-N. and Harambat, J. and Rothenbuhler, A. and Leger, J. and Kamenicky, P. and Soskin, S. and Boyer, O. and Boros, E. and D'anella, P. and Mignot, B. and Gebhart, M. and Vic, P. and Richard, N. and Thivichon-Prince, B. and Francou, B. and Linglart, A. and Bacchetta, J. and Molin, A.
Anticipated effects of burosumab treatment on long-term clinical sequelae in XLH: expert perspectives	2023	Frontiers in Endocrinology	Seefried, L. and Duplan, M.B. and Briot, K. and Collins, M.T. and Evans, R. and Florenzano, P. and Hawkins, N. and Javaid, M.K. and Lachmann, R. and Ward, L.M.
Nosology of genetic skeletal disorders: 2023 revision	2023	American Journal of Medical Genetics, Part A	Unger, S. and Ferreira, C.R. and Mortier, G.R. and Ali, H. and Bertola, D.R. and Calder, A. and Cohn, D.H. and Cormier-Daire, V. and Girisha, K.M. and Hall, C. and Krakow, D. and Makitie, O. and Mundlos, S. and Nishimura, G. and Robertson, S.P. and Savarirayan, R. and Sillence, D. and Simon, M. and Sutton, V.R. and Warman, M.L. and Superti-Furga, A.
FGF23 directly inhibits osteoprogenitor differentiation in Dmp1knockout mice	2023	JCI Insight	Courbon, G. and Kentrup, D. and Thomas, J.J. and Wang, X. and Tsai, H.-H. and Spindler, J. and Drasek, J.V. and Ndjonko, L.M. and Martinez-Calle, M. and Lynch, S. and

			Hivert, L. and Wang, X. and Chang, W. and Feng, J.Q. and David, V. and Martin, A.
Blocking FGF23 signaling improves the growth plate of mice with X-linked hypophosphatemia	2023	Journal of Endocrinology	Fuente, R. and Pastor, E.-M. and Gehring, N. and Carbajosa, P.O. and Alonso-Durán, L. and Zderic, I. and Tapia-Dean, J. and Hamid, A.K. and Bettoni, C. and Santos, F. and Wagner, C.A. and Rubio-Aliaga, I.
Presentation and Diagnosis of Pediatric X-Linked Hypophosphatemia	2023	Endocrines	Ikegawa, K. and Hasegawa, Y.
Intracranial calcification in Fam20c-deficient mice recapitulates human Raine syndrome	2023	Neuroscience Letters	Zhang, H. and Lu, Y. and Kramer, P.R. and Benson, M.D. and Cheng, Y.-S.L. and Qin, C.
Combined gait analysis and radiologic examination in children with X-linked hypophosphatemia	2023	Clinical Biomechanics	Bonnet-Lebrun, A. and Linglart, A. and De Tienda, M. and Nguyen Khac, V. and Ouchrif, Y. and Berkenou, J. and Pillet, H. and Assi, A. and Wicart, P. and Skalli, W.
Wnt pathway inhibitors are upregulated in XLH dental pulp cells in response to odontogenic differentiation	2023	International Journal of Oral Science	Guirado, E. and Villani, C. and Petho, A. and Chen, Y. and Maienschein-Cline, M. and Lei, Z. and Los, N. and George, A.
Young XLH Patients-Reported Experience with a Supportive Care Program	2023	Patient Preference and Adherence	Rothenbuhler, A. and Gueorguieva, I. and Lichtenberger-Geslin, L. and Audrain, C. and Soskin, S. and Bensignor, C. and Rossignol, S. and Bertholet-Thomas, A. and Naudeau, L. and Bacchetta, J. and Linglart, A.
Health-related quality of life of children with X-linked hypophosphatemia in Germany	2024	Pediatr. Nephrol.	Klein, M. and Obermaier, M. and Mutze, H. and Wilden, S.M. and Rehberg, M. and Schlingmann, K.P. and Schmidt, D. and Metzing, O. and Hübner, A. and Richter-Unruh, A. and Kemper, M.J. and Weitz, M. and Wühl, E. and Jorch, N. and Patzer, L. and Freiberg, C. and Heger, S. and Ziviknjak, M. and Schnabel, D. and Haffner, D.
Association between work productivity and characteristics of adults with X-linked hypophosphatemia: an analysis of the XLH disease monitoring program	2024	JBMR Plus	Khan, A. and Johnson, B. and Nixon, A. and Dent, J.E. and Li, Z. and Yang, E. and Williams, A.

Orthodontic treatment in children and adolescent patients with X-linked hypophosphatemia: A case-control study	2024	Orthod Craniofac Res	Janssens, Y. and Duplan, M.B. and Linglart, A. and Rothenbuhler, A. and Chaussain, C. and Le Norcy, E.
Epidemiological analysis to identify predictors of X-linked hypophosphatemia (XLH) diagnosis in an Italian pediatric population: the EPIX project	2024	Endocrine	Crisafulli, S. and Ingrasciotta, Y. and Vitturi, G. and Fontana, A. and L'Abbate, L. and Alessi, Y. and Ferraù, F. and Cantarutti, L. and Lazzerini, D. and Cannavò, S. and Trifirò, G.
NFATc1 Is Required for Vitamin D- and Phosphate-Mediated Regulation of Osteocyte Lacuno-Canalicular Remodeling	2024	Endocrinology	Jagga, S. and Hughes, A. and Arash, N.M. and Sorsby, M. and Brooks, D.J. and Pajevic, P.D. and Liu, E.S.
The role of GDF5 in regulating enthesopathy development in the Hyp mouse model of XLH	2024	J. Bone Miner. Res.	Sorsby, M. and Almardini, S. and Alayyat, A. and Hughes, A. and Venkat, S. and Rahman, M. and Baker, J. and Rana, R. and Rosen, V. and Liu, E.S.
X-linked hypophosphatemia: The value of feedback focus groups to assess patient and caregiver needs	2024	Eur. J. Med. Genet.	Wagner, E. and Bertholet-Thomas, A. and Romier, M. and Loin, L. and Lemoine, S. and Vignot, E. and Flammier, S. and Garnier, C. and De-Mul, A. and Feutrier, C. and Juillard, S. and Thivichon-Prince, B. and Lienhart, G. and Bacchetta, J.
Complex intrinsic abnormalities in osteoblast lineage cells of X-linked hypophosphatemia: Analysis of human iPS cell models generated by CRISPR/Cas9-mediated gene ablation	2024	Bone	Nakanishi, T. and Yamazaki, M. and Tachikawa, K. and Ueta, A. and Kawai, M. and Ozono, K. and Michigami, T.
Oral Health-Related Quality of Life in Italian Children and Adolescents Living with Bone Dysplasia: A Cross-Sectional Study	2024	Int. J. Environ. Res. Public Health	Defabianis, P. and Ninivaggi, R. and Tessaris, D. and Bocca, N. and Romano, F.
Oral health and oral-health-related quality of life in people with X-linked hypophosphatemia	2024	BMC Oral Health	Steur, J. and Bohner, L. and Jackowski, J. and Hanisch, M. and Oelerich, O.
A PAI-1 antagonist ameliorates hypophosphatemia in the Hyp vitamin D-resistant rickets model mouse	2024	FEBS Open Bio.	Qian, C. and Ito, N. and Tsuji, K. and Sato, S. and Kikuchi, K. and Yoshii, T. and Miyata, T. and Asou, Y.
FGF23-related hypophosphatemic rickets preceding the onset of systemic lupus erythematosus: A juvenile case	2024	Clin. Case Rep.	tabei, Y. and Ohtsu, Y. and Shimada, M. and Wada, A. and Hamajima, E. and Osawa, Y. and Takizawa, T.

The sacroiliac joint: An original and highly sensitive tool to highlight altered bone phenotype in murine models of skeletal disorders	2024	Bone	Hilliquin, S. and Zhukouskaya, V. and Fogel, O. and Cherifi, C. and Ibrahim, K. and Slimani, L. and Cornelis, F.M.F. and Storms, L. and Hens, A. and Briot, K. and Lories, R. and Chaussain, C. and Miceli-Richard, C. and Bardet, C.
X-linked hypophosphatemic rickets and nephrocalcinosis: clinical characteristics of a single-center pediatric cohort in North America before and after burosumab	2024	Front. Pediatr.	Paloian, N.J. and Boyke-Lohmann, L.R. and Steiner, R.D.
Genetics, X-Linked Inheritance.	2024		Basta M and Pandya AM
Rare diseases: a challenge in paediatric dentistry.	2024	European journal of paediatric dentistry	Giuca MR
Rare Causes of Musculoskeletal Pain: Thinking beyond Common Rheumatologic Diseases.	2024	Case reports in rheumatology	Charles JF and Malabanan AO and Krolczyk S and Dahir KM
Inherited phosphate and pyrophosphate disorders: New insights and novel therapies changing the oral health landscape.	2024	Journal of the American Dental Association (1939)	Foster BL and Boyce AM and Millán JL and Kramer K and Ferreira CR and Somerman MJ and Wright JT
Alopecia with Vitamin D-Dependent Rickets Type 2 A: A Case Report.	2024	Clinical, cosmetic and investigational dermatology	Bin Rubaian NF and Al-Awam BS and Aljohani SM and Almuhaidib SR
Editorial: New mechanistic insights into mineral regulatory hormones (FGF23/Klotho, PTH, and vitamin D) including genetic and epigenetic pathways.	2024	Frontiers in endocrinology	Ide N and Courbebaisse M and Mencke R and Hanai JI
Effect of Mutation Type on Ectopic Ossification Among Adult Patients With X-Linked Hypophosphatemia.	2024	Journal of the Endocrine Society	Kato H and Ishihara Y and Ohata Y and Irie K and Watanabe S and Kimura S and Hoshino Y and Hidaka N and Kinoshita Y and Taniguchi Y and Kobayashi H and Braddock DT and Kubota T and Ozono K and Nangaku M and Makita N and Ito N
Real-world non-interventional post-authorization safety study of long-term use of burosumab in children and adolescents with X-linked hypophosphatemia: first interim analysis	2024	Therapeutic Advances in Chronic Disease	M. Boot, A. and Ariceta, G. and Beck-Nielsen, S.S. and Brandi, M.L. and Briot, K. and Collantes, C.D.L. and Giannini, S. and Haffner, D. and Keen, R. and Levtchenko, E. and Mughal, M.Z. and Makitie, O. and Nilsson, O. and Schnabel, D. and Tripto-Shkolnik, L. and Zillikens, M.C. and Liu, J. and Tudor, A. and Emma, F.
X-Linked Hypophosphatemia: A case report	2024	Revista Ciencias de la Salud	Tascón Arcila, J.A. and Baquero Rodríguez, M.C. and Serrano Gayubo, A.K. and Baquero Rodríguez, R.

Lessons learned from the real-world diagnosis and management of hereditary hypophosphatemic rickets	2024	Bone Reports	Chaturvedi, D. and Mehasi, T.E. and Benbrahim, A. and ElDeeb, L. and Deeb, A.
Latin-American consensus on the transition into adult life of patients with X-linked hypophosphatemia	2024	Endocrine	Kastelic, M.S. and Roman-González, A. and De Paula Colares Neto, G. and De Paula, F.J.A. and Reza-Albarrán, A.A. and Morales, L.R. and Tormo, S. and Meza-Martínez, A.I.
CYP4A22 loss-of-function causes a new type of vitamin D–dependent rickets (VDDR1C)	2024	Journal of Bone and Mineral Research	Duan, X. and Zhang, Y. and Xu, T.
Tubular phosphate transport: a comparison between different methods of urine sample collection in FGF23-dependent hypophosphatemic syndromes	2024	Clinical Chemistry and Laboratory Medicine	Arcidiacono, G.P. and Camozzi, V. and Zaninotto, M. and Tripepi, G. and Fusaro, M. and Torres, M.O. and Zanchetta, F. and Cannito, M. and Cecchinato, A. and Diogo, M. and Falb, M.P. and Plebani, M. and Simioni, P. and Sella, S. and Giannini, S.
Human genetic diseases of phosphate and pyrophosphate metabolism	2024	Archives de Pediatrie	Molin, A.
Craniofacial and dental characteristics of patients with vitamin-D-dependent rickets type 1A compared to controls and patients with X-linked hypophosphatemia	2018	Clinical Oral Investigations	Gjørup, H. and Beck-Nielsen, S.S. and Haubek, D.
Tooth development associated with mutations in hereditary vitamin D–resistant rickets	2018	JDR Clinical and Translational Research	Hanna, A.E. and Sanjad, S. and Andary, R. and Nemer, G. and Ghafari, J.G.
Dental alterations associated with X-linked hypophosphatemic rickets	2004	Journal of Endodontics	Pereira, C.M. and De Andrade, C.R. and Vargas, P.A. and Della Coletta, R. and De Almeida, O.P. and Lopes, M.A.
The association of dental abscesses with vitamin D resistant rickets	1983	British Dental Journal	Tulloch, E.N. and Andrews, F.F.
[Familial persistent phosphatic diabetes with D vitamin resistant rachitis; findings in two deciduous teeth].	1953	Helvetica paediatrica acta	SCHMUZIGER P
Gross tooth hypocalcification in vitamin D-resistant rickets	1959	Australian Dental Journal	Hall, R.K.
Dental sequelæ in deciduous dentition in vitamin D resistant rickets: Case Report	1960	Australian Dental Journal	Harris, R. and Sullivan, H.R.
VITAMIN D-RESISTANT RICKETS. ANALYSIS OF TWENTY-FOUR PEDIGREES WITH HEREDITARY AND SPORADIC CASES.	1964	The American journal of medicine	BURNETT CH and DENT CE and HARPER C and WARLAND BJ
Dental and cephalometric findings in vitamin D resistant rickets.	1965	Journal of dentistry for children	Marks, S.C. and Lindahl, R.L. and Bawden, J.W.
Hereditary hypophosphatemia (vitamin D-resistant rickets) presenting primary dental manifestations	1966	Oral Surgery, Oral Medicine, Oral Pathology	Archard, H.O. and Witkop Jr., C.J.
Microradiographic and polarized-light study of dental tissues in vitamin D-resistant rickets	1967	Oral Surgery, Oral Medicine, Oral Pathology	Soni, N.N. and Marks, S.C.

On three clinical cases of vitamin D resistant rickets	1967	Nihon Kyosei Shika Gakkai zasshi = The journal of Japan Orthodontic Society	Ishiki, Y. and Takahashi, I. and Maruyama, H.
Dentofacial development in children with vitamin D resistant rickets.	1968	Journal of the American Dental Association (1939)	Tracy, W.E. and Campbell, R.A.
Hereditary hypophosphatemia.	1969	ASDC journal of dentistry for children	Gardner DE and Davis WB and Prescott GH
Abnormal dentition in vitamin D resistant rickets. A case report.	1970	ASDC journal of dentistry for children	Wihr, N.L.
Tooth changes in genuine vitamin-D-resistant rickets	1970	SSO. Schweizerische Monatsschrift fur Zahnheilkunde. Revue mensuelle suisse d''odonto-stomatologia. Rivista mensile svizzera di odontologia e stomatologia	Schneider, H.R.
The dental defects of vitamin D-resistant rickets.	1971	Birth defects original article series	Archard, H.O.
Analysis of dentine pathogenesis in vitamin D-resistant rickets	1971	Oral Surgery, Oral Medicine, Oral Pathology	Tracy, W.E. and Steen, J.C. and Steiner, J.E. and Buist, N.R.M.
Vitamin D-resistant rickets with dental abnormalities.	1971	Birth defects original article series	Smith, W.K. and Steinhauser, R.A.
Electron optic analysis of human dentin in hypophosphatemic vitamin D-resistant rickets (Report of a kindred with consanguinity)	1973	Journal of Oral Pathology & Medicine	Sauk, J.J. and Witkop, C.J.
Oral manifestations of cystinosis	1973	Oral Surgery, Oral Medicine, Oral Pathology	Nazif, M. and Osman, M.
Osteomalacia and altered magnesium metabolism in the X-linked hypophosphatemic mouse	1979	CALCIF. TISSUE INT.	Meyer Jr., R.A. and Jowsey, J. and Meyer, M.H.
Distribution of the perilacunar hypomineralized areas in cortical bone from patients with familial hypophosphatemic (vitamin D-resistant) rickets	1979	Calcified Tissue International	Choufoer, J.H. and Steendijk, R.
Spontaneous dental abscesses in vitamin-D-resistant rickets: report of case.	1979	ASDC journal of dentistry for children	Gallo, L.G. and Merle, S.G.
Etiology of Enamel Hypoplasia and Interglobular Dentin: The Roles of Hypocalcemia and Hypophosphatemia	1979	Metabolic Bone Disease and Related Research	Nikiforuk, G. and Fraser, D.
