# Supplementary figures and images for: Dental Manifestations in Children Affected by Hypophosphatemic Rickets: A Systematic Review and Meta-Analysis
Source: Children (Basel). 2025 Jan 27;12(2):144. doi: 10.3390/children12020144 (PMC11854695; doi:10.3390/children12020144)

Figure S1: Forest plot of analysis based on risk of bias

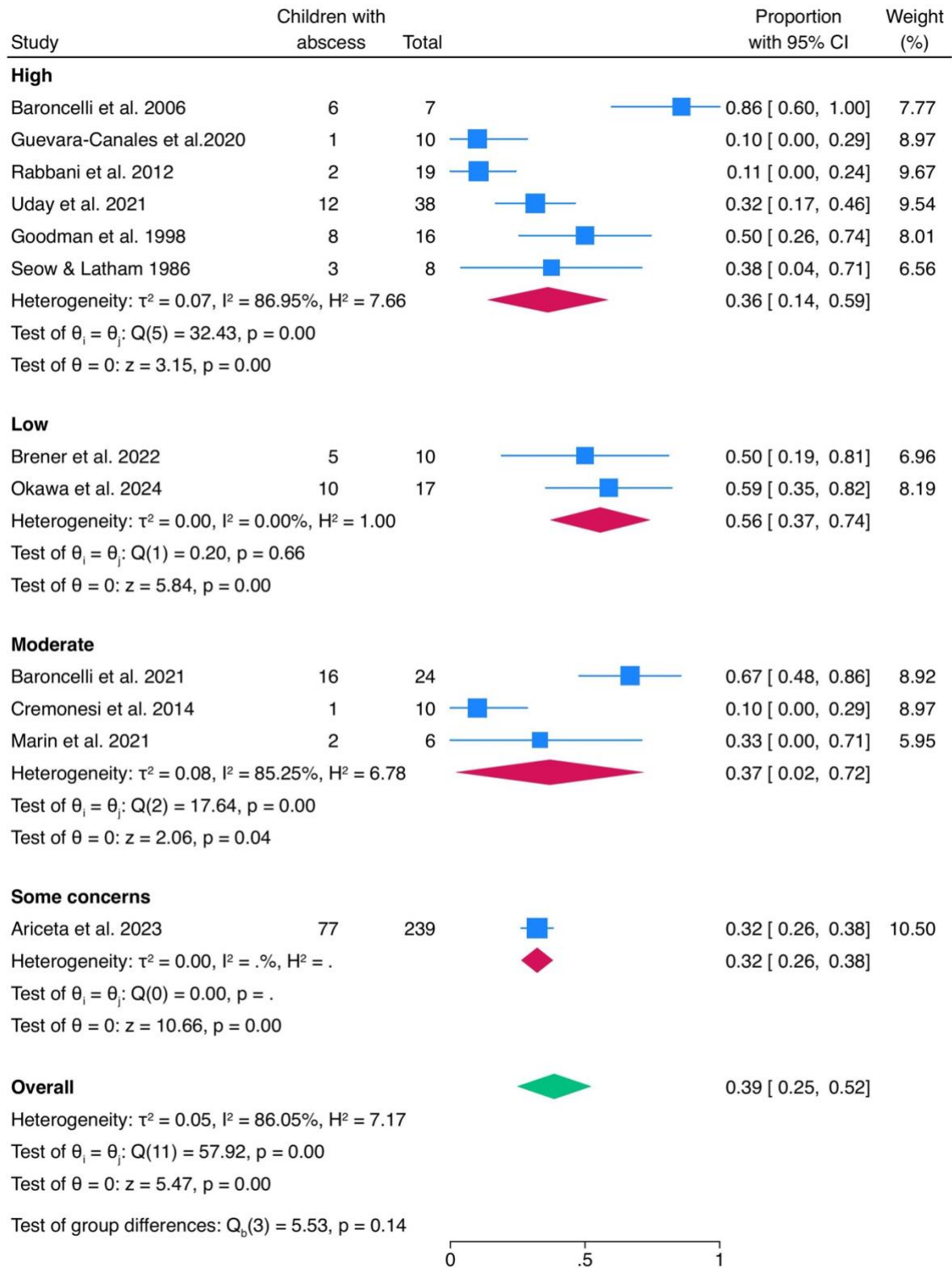

Random-effects REML model

Supplement: Supplementary file 1 [file children-12-00144-s001.zip › Figure S1.pdf]

Figure S2: Forest plot of analysis based on type of rickets

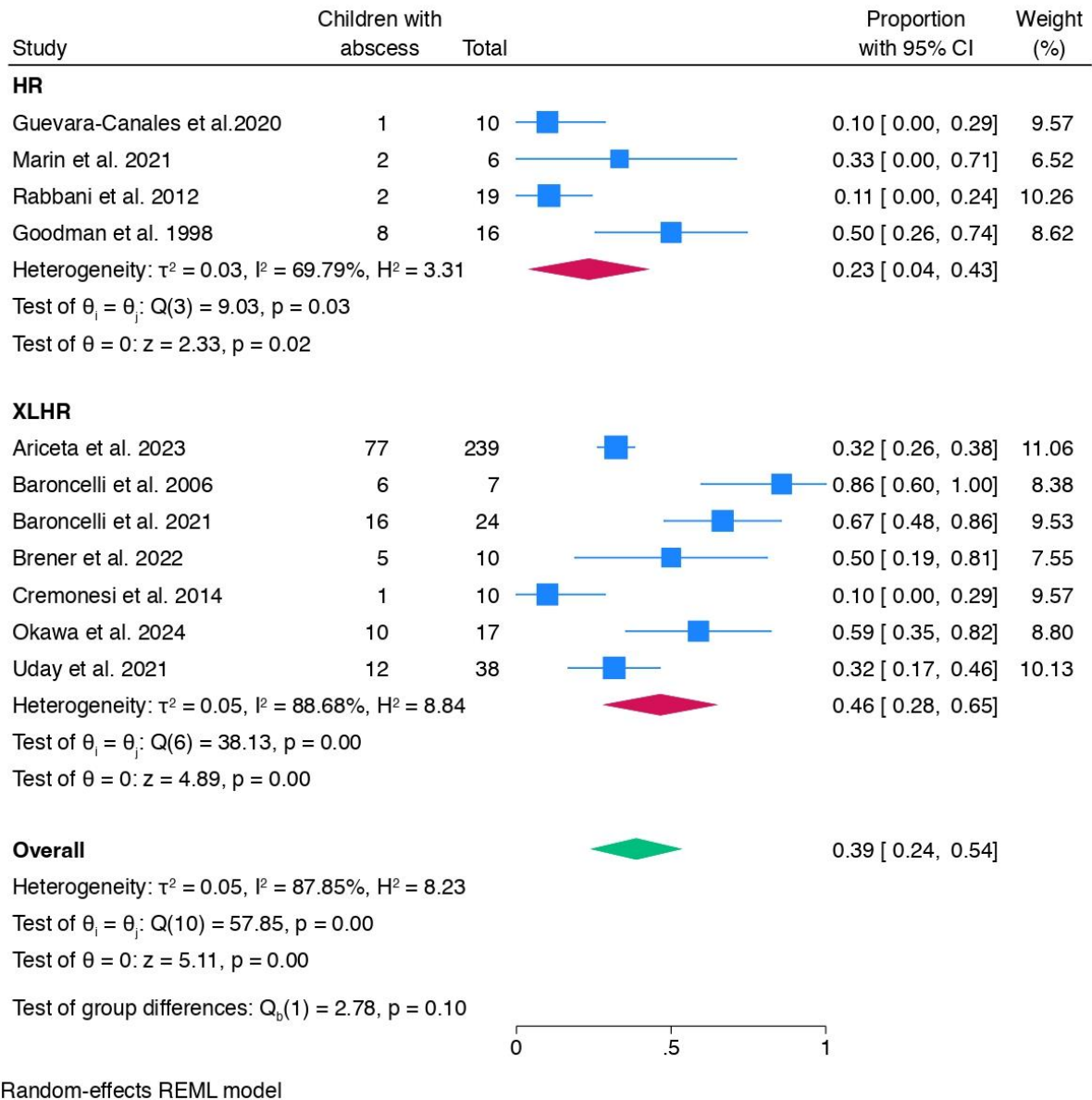

Supplement: Supplementary file 1 [file children-12-00144-s001.zip › Figure S2.pdf]
